# Supplementary material for: The role of stress and health behaviour in linking weight discrimination and health: a secondary data analysis in England
Source: BMJ Open. 2023 Sep 14;13(9):e072043. doi: 10.1136/bmjopen-2023-072043 (PMC10503332; doi:10.1136/bmjopen-2023-072043)
Supplement: Supplementary data [file bmjopen-2023-072043supp001.pdf]

Supplementary materials: Questionnaire measures

Everyday discrimination

In your day-to-day life, how often have any of the following things happened to you:

	Never (0)	Less than once a year (1)	Few times a year (2)	Few times a month (3)	At least once a week(4)	Almost everyday (5)
You are treated with less respect or courtesy						
You receive poorer service than other people in restaurants and stores						
People act as if they think you are not clever						
You are threatened or harassed						
You receive poorer service or treatment than other people from doctors or hospitals						

If you answered yes to any of the above, what do you attribute this experience to

Gender
Race
Age
Weight
Physical disability
Physical appearance
Sexual orientation
Financial status
Other reason

Self-rated health

"Would you say your health is… poor/fair/good/very good/excellent?"

Limiting long-standing illness

1) "Do you have any long-standing illness, disability, or infirmity? By long-standing I mean anything that has troubled you over a period of time or that is likely to affect you over a period of time."
2) If yes, "Does this illness or disability limit your activities in any way?"

Satisfaction With Life Scale

(Diener et al., The satisfaction with life scale. J Assess. 1985;49(1):71–5).

How much of the time during the past week...
1) In most ways my life is close to my ideal.
2) The conditions of my life are excellent.
3) I am satisfied with my life.
4) So far, I have gotten the important things I want in life.
5) If I could live my life over, I would change almost nothing.
Answers range from 0 (Strongly Disagree) to 6 (Strongly agree)

Center for Epidemiologic Studies Depression (CESD) scale

(Radloff LS. The CES-D scale. Appl Psychol Meas. 1977;1(3):385–401).

In the past month	Yes	No
1) ... did you feel depressed?		
2) ... did you feel everything you did was an effort?		
3) ... was your sleep restless?		
4) ... were you happy?		
5) ... did you feel lonely?		
6) ... did you enjoy life?		
7) ... did you feel sad?		
8) ... were you unable to get going?		

CASP-19

(Hyde M., et al., A measure of quality of life in early old age: the theory, development and properties of a needs satisfaction model (CASP-19). Aging Ment Health. 2003;7(3):186–94).

	Often (0)	Sometimes (1)	Not often (2)	Never (3)
**1** My age prevents me from doing the things I would like to				
**2** I feel that what happens to me is out of my control				
**3** I feel free to plan for the future				
**4** I feel left out of things				
**5** I can do the things I want to do				
**6** Family responsibilities prevent me from doing what I want to do				
**7** I feel that I can please myself what I do				
**8** My health stops me from doing things I want to				
**9** Shortage of money stops me from doing the things I want to do				
**10** I look forward to each day				
**11** I feel that my life has meaning				
**12** I enjoy the things that I do				
**13** I enjoy being in the company of others				
**14** On balance, I look back on my life with a sense of happiness				
**15** I feel full of energy these days				
**16** I choose to do things that I have never done before				
**17** I am satisfied with the way my life has turned out				
**18** I feel that life is full of opportunities				
**19** I feel that the future looks good for me				
