# Supplementary material for: The role of stress and health behaviour in linking weight discrimination and health: a secondary data analysis in England
Source: BMJ Open. 2023 Sep 14;13(9):e072043. doi: 10.1136/bmjopen-2023-072043 (PMC10503332; doi:10.1136/bmjopen-2023-072043)
Supplement: Supplementary data [file bmjopen-2023-072043supp002.pdf]

Sensitivity analyses - Tables

Supplementary Table 1 Sample characteristics at baseline (Wave 5) **all weight categories**

	Whole sample (*n*=5965)	Perceived weight discrimination (*n*=219)	No perceived weight discrimination (*n*=5746)	*p*
Age (years), mean (SD)	66.56 (8.13)	62.16 (6.74)	66.72 (8.13)	<0.001
Sex, % (*n*)				
Men	44.5 (2655)	35.2 (77)	44.9 (2578)	=0.005
Women	55.5 (3310)	64.8 (142)	55.1 (3168)	-
Ethnicity, % (*n*)				
White	97.9 (5841)	97.7 (214)	97.9 (5627)	0.829
Other	2.1 (124)	2.3 (5)	2.1 (199)	-
Wealth quintile, % (*n*)				
1 (poorest)	14.6 (868)	26.0 (57)	14.1 (811)	<0.001
2	19.8 (1183)	30.6 (67)	19.4 (1116)	-
3	19.9 (1190)	18.3 (40)	20.0 (1150)	-
4	21.9 (1306)	15.5 (34)	22.1 (1272)	-
5 (richest)	23.8 (1418)	9.6 (21)	24.3 (1397)	-
BMI (kg/m^2), mean (SD)	28.27 (5.22)	36.05 (6.69)	27.98 (4.92)	<0.001

BMI, body mass index.

Supplementary Table 2 Cross-sectional and prospective associations of perceived weight discrimination with health and wellbeing outcomes across **all weight categories**

	**Cross-sectional (Wave 5)**		**Prospective (Wave 7)**	
	**No perceived weight discrimination**	**Perceived weight discrimination**	**No perceived weight discrimination**	**Perceived weight discrimination**
Fair/poor self-rated health				
n included in analysis	5754	217	5122	197
% (*n*) reporting outcome	20.8 (1197)	48.4 (105)	24.4 (1251)	52.8 (104)
Adjusted OR [95% CI]<br>*p*	1.00 (ref)	2.32 [1.71; 3.16]<br><0.001	1.00 (ref)	1.66 [1.15; 2.42]<br>0.008
Limiting long-standing illness				
n included in analysis	5743	218	5172	199
% (*n*) reporting outcome	30.3 (1741)	55.0 (120)	33.9 (1755)	56.3 (112)
Adjusted OR [95% CI]<br>*p*	1.00 (ref)	1.91 [1.42; 2.58]<br><0.001	1.00 (ref)	1.32 [0.92; 1.88]<br>0.130
Depressive symptoms				
n included in analysis	5695	219	5027	197
% (*n*) reporting outcome	11.7 (668)	27.9 (61)	10.4 (525)	30.5 (60)
Adjusted OR [95% CI]<br>*p*	1.00 (ref)	2.05 [1.46; 2.89]<br><0.001	1.00 (ref)	2.74 [1.84; 4.09]<br><0.001
Quality of life (range 0-57)				
n included in analysis	5497	212	4318	162
Mean (SD) score	41.96 (8.34)	34.26 (9.55)	42.54 (8.38)	36.60 (9.40)
Adjusted *B* [95% CI]<br>*p*	Ref	-6.03 [-7.18; -4.88]<br><0.001	Ref	-0.21 [-1.12; 0.70]<br>0.651
Life satisfaction (range 0-30)				
n included in analysis	5577	207	4490	167
Mean (SD) score	21.07 (6.08)	17.49 (7.60)	21.28 (6.01)	18.32 (7.32)
Adjusted *B* [95% CI]<br>*p*	Ref	-2.50 [-3.37; -1.63]<br><0.001	Ref	-0.45 [-1.16; 0.26]<br>0.213

OR, odds ratio. CI, confidence interval. SD, standard deviation.
ORs, *B*s and 95% CIs are adjusted for age, sex, ethnicity, wealth, and body mass index. Prospective results are additionally adjusted for status/score on the outcome variable at baseline (Wave 5).
Ref = reference category.

Supplementary Table 3 Sample characteristics at baseline (Wave 5) **in obese participants**

		**Whole sample (*n*=1853)**	**Perceived weight discrimination (*n*=184)**	**No perceived weight discrimination (*n*=1669)**	***p***
Age (years), mean (SD)		65.81 (7.76)	62.03 (6.66)	66.23 (7.76)	<0.001
Sex, % (*n*)					
	Men	40.5 (751)	33.2 (61)	41.3 (690)	=0.032
	Women	59.5 (1102)	66.8 (123)	58.7 (979)	-
Ethnicity, % (*n*)					
	White	97.8 (1812)	97.3 (179)	97.8 (1633)	0.624
	Other	2.2 (41)	2.7 (5)	2.2 (36)	-
Wealth quintile, % (*n*)					
	1 (poorest)	19.9 (368)	27.7 (51)	19.0 (317)	<0.001
	2	22.9 (424)	29.3 (54)	22.2 (370)	-
	3	21.3 (395)	19.6 (36)	21.5 (359)	-
	4	19.3 (358)	15.8 (29)	19.7 (329)	-
	5 (richest)	16.6 (308)	7.6 (14)	17.6 (294)	-
BMI (kg/m^2), mean (SD)		34.31 (4.13)	37.80 (5.68)	33.92 (3.73)	<0.001

BMI, body mass index.

Supplementary Table 4 Cross-sectional and prospective associations of perceived weight discrimination with health and wellbeing outcomes across **in obese participants**

	Cross-sectional (Wave 5)		Prospective (Wave 7)	
	**No perceived weight discrimination**	**Perceived weight discrimination**	**No perceived weight discrimination**	**Perceived weight discrimination**
Fair/poor self-rated health				
n included in analysis	1669	182	1478	167
% (*n*) reporting outcome	28.4 (474)	47.3 (86)	32.3 (478)	53.9 (90)
Adjusted OR [95% CI]<br>*p*	1.00 (ref)	1.74 [1.23; 2.46]<br>=0.002	1.00 (ref)	1.56 [1.02; 2.39]<br>0.040
Limiting long-standing illness				
n included in analysis	1668	183	1493	169
% (*n*) reporting outcome	39 (651)	53.6 (98)	43.4 (648)	56.2 (95)
Adjusted OR [95% CI]<br>*p*	1.00 (ref)	1.47 [1.05; 2.01]<br>=0.027	1.00 (ref)	1.01 [0.70; 1.56]<br>0.832
Depressive symptoms				
n included in analysis	1654	184	1451	167
% (*n*) reporting outcome	14.1 (234)	28.8 (53)	11.9 (172)	29.9 (50)
Adjusted OR [95% CI]<br>*p*	1.00 (ref)	1.97 [1.34; 2.88]<br><0.001	1.00 (ref)	2.15 [1.36; 3.41]<br><0.001
Quality of life (range 0-57)				
n included in analysis	1581	178	1225	137
Mean (SD) score	40.85 (8.44)	34.44 (9.50)	41.22 (8.61)	36.61 (9.16)
Adjusted *B* [95% CI]<br>*p*	Ref	-5.24 [-6.57; -3.92]<br><0.001	Ref	0.08 [-1.01; 1.17]<br>0.888
Life satisfaction (range 0-30)				
n included in analysis	1604	173	1273	139
Mean (SD) score	20.57 (6.28)	17.51 (7.67)	20.66 (6.23)	18.60 (7.17)
Adjusted *B* [95% CI]<br>*p*	Ref	-2.00 [-3.03; -0.98]<br><0.001	Ref	0.09 [-0.75; 0.94]<br>0.820

OR, odds ratio. CI, confidence interval. SD, standard deviation.
ORs, *B*s and 95% CIs are adjusted for age, sex, ethnicity, wealth, and body mass index. Prospective results are additionally adjusted for status/score on the outcome variable at baseline (Wave 5).
Ref = reference category.

Supplementary Table 5 Cross-sectional and prospective associations of perceived weight discrimination in **any domain (ever vs never)** with health and wellbeing outcomes **in overweight and obese participants**

	Cross-sectional (Wave 5)		Prospective (Wave 7)	
	**No perceived weight discrimination**	**Perceived weight discrimination**	**No perceived weight discrimination**	**Perceived weight discrimination**
Fair/poor self-rated health				
n included in analysis	4090	248	3670	223
% (*n*) reporting outcome	22.5 (919)	44.0 (109)	26.2 (960)	51.1 (114)
Adjusted OR [95% CI]<br>*p*	1.00 (ref)	1.74 [1.29; 2.35]<br><0.001	1.00 (ref)	1.68 [1.17; 2.41]<br>0.005
Limiting long-standing illness				
n included in analysis	4089	249	3705	225
% (*n*) reporting outcome	32.0 (1309)	54.6 (136)	36.0 (1333)	57.3 (129)
Adjusted OR [95% CI]<br>*p*	1.00 (ref)	1.79 [1.34; 2.39]<br><0.001	1.00 (ref)	1.33 [0.94; 1.89]<br>0.105
Depressive symptoms				
n included in analysis	4058	250	3609	222
% (*n*) reporting outcome	11.9 (483)	25.6 (64)	10.7 (385)	28.4 (63)
Adjusted OR [95% CI]<br>*p*	1.00 (ref)	1.75 [1.24; 2.45]<br><0.001	1.00 (ref)	2.54 [1.71; 3.77]<br><0.001
Quality of life (range 0-57)				
n included in analysis	3902	243	3080	180
Mean (SD) score	41.76 (8.35)	34.85 (9.50)	42.20 (8.47)	37.17 (9.39)
Adjusted *B* [95% CI]<br>*p*	Ref	-5.53 [-6.64; -4.42]<br><0.001	Ref	0.24 [-0.67; 1.15]<br>0.608
Life satisfaction (range 0-30)				
n included in analysis	3968	239	3210	190
Mean (SD) score	21.05 (6.02)	18.05 (7.41)	21.22 (6.04)	18.89 (7.09)
Adjusted *B* [95% CI]<br>*p*	Ref	-1.87 [-2.70; -1.04]<br><0.001	Ref	-0.07 [-0.76; 0.62]<br>0.844

OR, odds ratio. CI, confidence interval. SD, standard deviation.
ORs, *B*s and 95% CIs are adjusted for age, sex, ethnicity, wealth, and body mass index. Prospective results are additionally adjusted for status/score on the outcome variable at baseline (Wave 5).
Ref = reference category.
