# Supplementary material for: The role of stress and health behaviour in linking weight discrimination and health: a secondary data analysis in England
Source: BMJ Open. 2023 Sep 14;13(9):e072043. doi: 10.1136/bmjopen-2023-072043 (PMC10503332; doi:10.1136/bmjopen-2023-072043)
Supplement: Author's manuscript [file bmjopen-2023-072043.draft_revisions.pdf]

BMJ Open is committed to open peer review. As part of this commitment we make the peer review history of every article we publish publicly available.

When an article is published we post the peer reviewers' comments and the authors' responses online. We also post the versions of the paper that were used during peer review. These are the versions that the peer review comments apply to.

The versions of the paper that follow are the versions that were submitted during the peer review process. They are not the versions of record or the final published versions. They should not be cited or distributed as the published version of this manuscript.

BMJ Open is an open access journal and the full, final, typeset and author-corrected version of record of the manuscript is available on our site with no access controls, subscription charges or pay-per-view fees (http://bmjopen.bmj.com).

If you have any questions on BMJ Open's open peer review process please email info.bmjopen@bmj.com

BMJ Open

Weight discrimination and health: Assessing the mediating effects of stress and health-risk behaviours in a prospective cohort study

Journal:	*BMJ Open*
Manuscript ID	bmjopen-2023-072043
Article Type:	Original research
Date Submitted by the Author:	23-Jan-2023

Complete List of Authors:	Hackett, Ruth; King’s College London, Department of Psychology; University College London, Department of Behavioural Science and Health<br>Jackson, Sarah ; University College London, Epidemiology and Public Health<br>Corker, Elizabeth; University College London, Clinical; University of Sheffield, Department of Psychology<br>Steptoe, Andrew; University College London, Department of Behavioural Science and Health

Keywords:	EPIDEMIOLOGY, PUBLIC HEALTH, Depression & mood disorders < PSYCHIATRY

SCHOLARONE™
Manuscripts

I, the Submitting Author has the right to grant and does grant on behalf of all authors of the Work (as defined in the below author licence), an exclusive licence and/or a non-exclusive licence for contributions from authors who are: i) UK Crown employees; ii) where BMJ has agreed a CC-BY licence shall apply, and/or iii) in accordance with the terms applicable for US Federal Government officers or employees acting as part of their official duties; on a worldwide, perpetual, irrevocable, royalty-free basis to BMJ Publishing Group Ltd ("BMJ") its licensees and where the relevant Journal is co-owned by BMJ to the co-owners of the Journal, to publish the Work in this journal and any other BMJ products and to exploit all rights, as set out in our licence.

The Submitting Author accepts and understands that any supply made under these terms is made by BMJ to the Submitting Author unless you are acting as an employee on behalf of your employer or a postgraduate student of an affiliated institution which is paying any applicable article publishing charge ("APC") for Open Access articles. Where the Submitting Author wishes to make the Work available on an Open Access basis (and intends to pay the relevant APC), the terms of reuse of such Open Access shall be governed by a Creative Commons licence – details of these licences and which Creative Commons licence will apply to this Work are set out in our licence referred to above.

Other than as permitted in any relevant BMJ Author's Self Archiving Policies, I confirm this Work has not been accepted for publication elsewhere, is not being considered for publication elsewhere and does not duplicate material already published. I confirm all authors consent to publication of this Work and authorise the granting of this licence.

For peer review only

Weight discrimination and health: Assessing the mediating effects of stress and health-risk behaviours in a prospective cohort study

Ruth A. Hackett[1,2], Sarah E. Jackson[2], Elizabeth Corker[3,4], Andrew Steptoe[2]

[1] Health Psychology Section, Department of Psychology, Institute of Psychiatry, Psychology and Neuroscience, King's College London, London, UK

[2] Department of Behavioural Science and Health, University College London, London, UK

[3] Clinical, Educational and Health Psychology, University College London, London, UK

[4] Clinical and Applied Psychology Unit, Department of Psychology, University of Sheffield, Sheffield, UK

Running head: Weight discrimination, health and wellbeing

Corresponding author: Dr Ruth A Hackett, Health Psychology Section, Department of Psychology, Institute of Psychiatry, Psychology and Neuroscience, King's College London, Great Maze Pond, London SE1 9RT, UK. ruth.hackett@kcl.ac.uk +44 (0) 207 188 5410

Competing interests: None

Word count: 3,461

For peer review only

For peer review only - http://bmjopen.bmj.com/site/about/guidelines.xhtml

Abstract

Objective: To examine the role of stress and health-risk behaviours in relationships between weight discrimination and health and wellbeing.

Design: Prospective observational cohort study

Setting: The English Longitudinal Study of Ageing

Participants: Data were from 4,341 adults (≥50y) with overweight/obesity.

Primary Outcome Measures: We tested associations between perceived weight discrimination at baseline (2010/11) and self-rated health, limiting long-standing illness, depressive symptoms, quality of life, and life satisfaction over four-year follow-up (2010/11; 2014/15). Potential mediation by stress exposure (hair cortisol) and health-risk behaviours (smoking, physical inactivity, alcohol consumption) was assessed.

Results: Cross-sectionally, perceived weight discrimination was associated with higher odds of fair/poor self-rated health (OR=2.05[95%CI 1.49;2.82]), limiting long-standing illness (OR=1.76[1.29;2.41]) and depressive symptoms (OR=2.01[1.41;2.85]), and lower quality of life (*B*=-5.82[95%CI -7.01;-4.62]) and life satisfaction (*B*=-2.36[-3.25;-1.47]). Prospectively, weight discrimination was associated with higher odds of fair/poor self-rated health (OR=1.63[1.10;2.40]) and depressive symptoms (OR=2.37[95%CI 1.57;3.60]) adjusting for baseline status. Those who reported discrimination had higher hair cortisol concentrations (*B*=0.14[0.03;0.25]) and higher odds of physical inactivity (OR=1.90[1.18;3.05]). These variables did not significantly mediate associations between discrimination and health outcomes.

Conclusions: Weight discrimination is associated with poor health and wellbeing. While this discrimination is associated with stress exposure and physical inactivity, these variables explain little of the association between discrimination and poorer outcomes.

Key words: weight discrimination; stigma; obesity; physical activity; smoking; health; wellbeing

For peer review only - http://bmjopen.bmj.com/site/about/guidelines.xhtml

Strengths and limitations

- Large prospective observational study
- Assessment of multiple health and wellbeing outcomes
- The prevalence of weight discrimination in the sample was low
- Measures of health behaviour were self-reported
- The sample was compromised of mostly white middle age and older adults

For peer review only

For peer review only - http://bmjopen.bmj.com/site/about/guidelines.xhtml

Introduction

The average person in the United Kingdom (UK) carries excess weight (1,2). Recent decades have seen an increase in the proportion of adults with a body mass index (BMI) in the overweight (25-29.9kg/m^2) or obesity (≥30kg/m^2) range, and a shift in the distribution of cases such that a greater proportion have 'morbid' obesity (BMI ≥40kg/m^2) (3). Despite the increasingly normative nature of obesity, it is widely stigmatised with those with obesity characterised as lazy, unintelligent, and lacking in self-discipline (4). These stereotypes translate into weight discrimination across contexts and interactions, including the workplace, education, healthcare settings, and interpersonal relationships (4).

Mounting evidence suggests that weight discrimination can impact health and wellbeing (5,6). Studies have linked perceived weight discrimination to adverse outcomes, including poorer self-rated health, greater disease burden, functional disability, psychiatric comorbidity, psychosocial stress, loneliness, poorer life satisfaction, and increased risk of premature death (7–13). Evidence is suggestive of a prospective relationship (i.e. discrimination preceding a decline in health) which persists after adjustment for BMI (7,9). It is particularly important to analyse these issues in older people, among whom disease and disability risk is elevated.

Several mechanisms may underpin the relationship between perceived weight discrimination and poorer health and wellbeing. Prominent theories focus on the role of (i) stress responses to discrimination and (ii) health-risk behaviours as a means of coping with or avoiding discrimination (14). Experiencing weight discrimination can be stressful, both physiologically, in the form of cortisol responses (15,16), and psychologically, in the form of increased negative emotion (8). If an individual perceives discrimination regularly, as is common with weight stigma (17), stress responses will be activated frequently, potentially leading to chronically elevated cortisol and a negative emotional state, which may in turn lead to ill health under allostatic load theory (18). Indeed, observational studies have associated elevated cortisol levels and weight discrimination (19,20).

Health-risk behaviours may also link weight discrimination to poorer health and wellbeing. These may emerge as a biological response to discrimination, for example, hypothalamic-pituitary-adrenal (HPA) axis activation upregulating appetite and the drive to consume energy-dense 'comfort' foods (21–23). In this way, weight stigma may contribute to a vicious cycle of stress, weight gain and further discrimination (14). Unhealthy behaviours may also provide a coping mechanism: food, nicotine, and alcohol activate

For peer review only - http://bmjopen.bmj.com/site/about/guidelines.xhtml

dopaminergic reward pathways in the brain (24) and may help to alleviate the negative psychological impact of discrimination. Avoiding activities for fear of discrimination (e.g. exercising in public places) may also act as a barrier to a healthy lifestyle (25). To our knowledge, no studies have investigated the extent to which these variables mediate associations between weight-based discrimination and health and wellbeing outcomes.

The present study used data from English Longitudinal Study of Ageing (ELSA) to examine (i) cross-sectional and prospective associations between perceived weight discrimination and health and wellbeing (self-rated health, limiting long-standing illness, depressive symptoms, quality of life, and life satisfaction) and (ii) whether these associations were mediated by stress exposure and health-risk behaviours (smoking, physical inactivity, and alcohol consumption).

Methods

Study population

ELSA is a representative longitudinal study of adults aged ≥50 living in England (27). Established in 2002, ELSA collects data via computer-assisted personal interviews and self-completion questionnaires biennially, with nurse visits in alternate waves to collect objective health measures, including BMI.

We used data on perceived weight discrimination from Wave 5 (2010/11; the only wave in which discrimination was assessed); hair cortisol, health behaviours, and BMI from Wave 6 (2012/13), and health and wellbeing from Waves 5 and 7 (2014/15). We restricted our sample to those with a BMI in the overweight or obese range (≥25kg/m^2).

Ethical approval for ELSA was obtained from the London Multicentre Research and Ethics Committee (MREC/01/02/91). All participants provided informed consent.

Patient and public involvement

No patient involved

For peer review only - http://bmjopen.bmj.com/site/about/guidelines.xhtml

Measures

Exposure: perceived weight discrimination

Participants were asked how often they encounter five discriminatory situations: *"In your day-to-day life, how often have any of the following things happened to you: 1) you are treated with less respect or courtesy; 2) you receive poorer service than other people in restaurants and stores; 3) people act as if they think you are not clever; 4) you are threatened or harassed; and 5) you receive poorer service or treatment than other people from doctors or hospitals."*. Response options were on a 5-point scale ranging from "*never*" to "*almost every day*." Because data were skewed, with most participants reporting no discrimination, we dichotomised responses to indicate whether or not participants had experienced discrimination in the past year (a few times or more a year vs. less than once a year or never), with the exception of the fifth item which was dichotomised to indicate whether or not respondents had ever experienced discrimination from doctors or hospitals (never vs. all other options) (28).

A follow-up question asked participants who reported discrimination to indicate the reason(s) they attributed to their experience (e.g., weight, age, sex, race, etc.,) Those who attributed any experience of discrimination to their weight are treated as cases of perceived weight discrimination as in other studies (19,29–33).

Outcomes: health and wellbeing

Self-rated health was assessed using a single item: *"Would you say your health is… poor/fair/good/very good/excellent?"* We analysed the proportion of individuals rating their health as fair/poor, as in other investigations (28,34). As a sensitivity check, analyses were also conducted using the continuous range of scores.

Limiting long-standing illness was assessed with two questions: 1) "*Do you have any long-standing illness, disability, or infirmity? By long-standing I mean anything that has troubled you over a period of time or that is likely to affect you over a period of time*." Those who responded yes were asked: 2) "*Does this illness or disability limit your activities in any way?*" Those who responded yes to both items were classed as having a limiting long-standing illness.

Depressive symptoms were assessed with the eight-item Center for Epidemiologic Studies Depression Scale (35). Respondents were asked if they had experienced depressive symptoms (e.g. restless sleep and

For peer review only - http://bmjopen.bmj.com/site/about/guidelines.xhtml

being unhappy) over the past month using a binary (yes/no) response. Total scores ranged from 0 to 8. Data were dichotomised using an established cut-off (scores ≥4) (36). As a sensitivity check, analyses were also conducted using the continuous range of scores.

Quality of life was assessed with the CASP-19 (37). Items covered four domains of quality of life; control (e.g., '*I feel that what happens to me is out of my control*'), autonomy (e.g., '*My health stops me from doing things I want to do*'), self-realisation (e.g., '*I feel that life is full of opportunities*'), and pleasure (e.g., '*I enjoy being in the company of others*'). Respondents were asked how often each statement applies to them (often=0, sometimes=1, not often=2, never=3). Positively-worded items were reverse scored so that a higher total score indicated higher quality of life (range: 0–57).

Life satisfaction was assessed with the 5-item Satisfaction With Life Scale (38), which asks the extent to which participants agree with statements including '*In most ways my life is close to my ideal*'; 'and '*I am satisfied with my life*';. Responses ranged from 0 (strongly disagree) to 6 (strongly agree) and were summed to produce a total score of 0-30.

Mediators: stress exposure and health-risk behaviours

The hair cortisol assessment procedure has been described in detail elsewhere (39). Briefly, a scalp-proximal hair sample at least 2cm long and weighing at least 10mg was taken from the posterior vertex, cut as close to the scalp as possible. The wash procedure and steroid extraction were undertaken using high performance liquid chromatography–mass spectrometry. Assuming an average hair growth of 1cm per month the 2cm hair segment represents average cortisol accumulated two months prior to sampling.

Smoking status was assessed with the question "*Do you smoke cigarettes at all nowadays?*" (yes/no).

Physical activity was self-reported in response to three questions on the frequency of participation in light, moderate and vigorous activities (more than once a week/once a week/one to three times a month/hardly ever or never). We compared participants who were physically inactive (defined as no activity on a weekly basis) with those who reported engaging in light, moderate, or vigorous activity at least once a week.

Alcohol consumption was assessed with the question "*On how many days out of the last seven did you have an alcoholic drink?*". Those who responded 'five', 'six' or 'seven' days were classified as frequent alcohol drinkers. We compared frequent alcohol drinkers with all other categories combined.

For peer review only - http://bmjopen.bmj.com/site/about/guidelines.xhtml

Covariates

Participants self-reported their age, sex, and ethnicity (white, other). Socioeconomic status was indexed with a measure of household non-pension wealth (27)), divided into quintiles across the entire Wave 5 sample. BMI (m^2/kg) was based on objective height and weight measurements.

Statistical analysis

Associations between perceived weight discrimination and covariates were examined using one-way independent analysis of variance (ANOVA) for continuous variables and χ^2 tests for categorical variables.

For our primary analyses, we used multivariable regression models (logistic for categorical outcomes, linear for continuous outcomes) to explore cross-sectional and prospective associations between perceived weight discrimination and health and wellbeing. All models were adjusted for age, sex, ethnicity, wealth, and BMI. Prospective models were additionally adjusted for baseline status/score on the outcome variable.

Where perceived weight discrimination was significantly associated with an outcome in prospective analyses, we tested for mediation by stress exposure and health-risk behaviours (Figure 1). Hair cortisol data was log transformed to correct skewness. Establishing mediation requires the mediator to be associated with the exposure (path *a*) and the outcome (path *b*), so we first tested associations of (i) perceived weight discrimination with (a) stress exposure (hair cortisol concentration) using linear regression and (b) health-risk behaviours (smoking, physical inactivity, and alcohol consumption) using logistic regression; and (ii) stress exposure and health-risk behaviours with health and wellbeing outcomes using logistic or linear regression, as appropriate. Where associations were significant, we used the *sgmediation* command in STATA, which calculates total (path *c*), direct (path *c'*), and indirect (path $a \times b$) effects, and tests the significance of the indirect effect using the Sobel test. We used bootstrapping with 5,000 sampling replications to estimate the 95% confidence interval and calculated effect ratios reflecting the proportion of the total effect of the independent variable on the dependent variable that is explained by the mediator. Mediation models were adjusted for age, sex, ethnicity, wealth, BMI, and status/score on the outcome variable at baseline.

We conducted two sensitivity analyses. In the first we expanded our analytic sample to include participants who provided data on perceived weight discrimination but did not have a BMI ≥25m^2/kg. We assessed whether the pattern of associations between perceived weight discrimination and health and wellbeing

For peer review only - http://bmjopen.bmj.com/site/about/guidelines.xhtml

outcomes was similar when including these participants. For our second sensitivity analysis we changed the frequency weight discrimination was encountered from *'in the past year vs less than once a year/never'* (main analysis) to whether the participant *ever* experienced discrimination (*'never' vs 'almost every day; at least once a week; a few times a month; a few times a year; less than once a year'*). We assessed whether our results changed when using this different definition of weight discrimination. Analyses were performed on complete cases using SPSS v.25, with the exception of the mediation models which were run in STATA v.13.

Results

In Wave 5, 9,090 participants (77.5% of those eligible) took part in the face-to-face interview, of whom 8,107 (93%) returned the self-completion questionnaire. We excluded 2,142 participants with missing data on weight discrimination or covariates (including BMI) and 1,624 with BMI <25kg/m^2. Our final sample for cross-sectional analysis comprised of 4,341 people, aged 52-89 years with a BMI 25.0-59.4kg/m^2.

Perceived weight discrimination was reported by 210 (4.8%) respondents, who were on average younger, heavier, more socioeconomically disadvantaged and more likely to be female than those who did not report weight discrimination (Table 1).

Table 1 Sample characteristics at baseline (Wave 5)

	**Whole sample (*n*=4341)**	**Perceived weight discrimination (*n*=210)**	**No perceived weight discrimination (*n*=4131)**	***p***
Age (years), mean (SD)	66.62 (7.96)	62.04 (6.58)	66.85 (7.95)	<0.001
Sex, % (*n*)				
Men	47.2 (2051)	34.8 (73)	47.9 (1978)	<0.001
Women	52.8 (2290)	65.2 (137)	52.1 (2153)	-
Ethnicity, % (*n*)				
White	97.9 (4250)	97.6 (205)	97.9 (4045)	0.768
Other	2.1 (91)	2.4 (5)	2.1 (86)	-
Wealth quintile, % (*n*)				
1 (poorest)	15.5 (672)	26.7 (56)	14.9 (616)	<0.001
2	20.6 (896)	29.0 (61)	20.2 (835)	-
3	21.0 (913)	18.6 (39)	21.2 (874)	-
4	21.8 (946)	16.2 (34)	22.1 (912)	-
5 (richest)	21.1 (914)	9.5 (20)	21.6 (894)	-
BMI (kg/m^2), mean (SD)	30.37 (4.47)	36.63 (6.17)	30.05 (4.12)	<0.001

BMI, body mass index.

For peer review only - http://bmjopen.bmj.com/site/about/guidelines.xhtml

Cross-sectionally, perceived weight discrimination was associated with significantly higher odds of fair/poor self-rated health, limiting long-standing illness, and depressive symptoms, and lower mean ratings of quality of life and life satisfaction, after adjustment for age, sex, ethnicity, wealth, and BMI (Table 2). Prospectively, perceived weight discrimination was associated with significantly higher odds of future fair/poor self-rated health and depressive symptoms, but differences in limiting long-standing illness, quality of life, and life satisfaction were not statistically significant after adjustment for covariates including baseline status/score on the outcome variable (Table 2). Analysing depression and self-rated health as continuous variables did not change the pattern of results (Table 2).

For peer review only

For peer review only - http://bmjopen.bmj.com/site/about/guidelines.xhtml

Table 2 Cross-sectional and prospective associations of perceived weight discrimination with health and wellbeing outcomes (path *c* on Figure 1)

		Cross-sectional (Wave 5)		Prospective (Wave 7)	
		**No perceived weight discrimination**	**Perceived weight discrimination**	**No perceived weight discrimination**	**Perceived weight discrimination**
Fair/poor self-rated health					
	n included in analysis	4130	208	3705	188
	% (*n*) reporting outcome	22.5 (928)	48.1 (100)	26.3 (973)	53.7 (101)
	Adjusted OR [95% CI]	1.00 (ref)	2.05 [1.49; 2.82]	1.00 (ref)	1.63 [1.10; 2.40]
	p		<0.001		0.014
Self-rated health (range 1-5)					
	n included in analysis	4130	208	3705	188
	Mean (SD) score	2.73 (1.04)	3.47 (1.06)	2.85 (1.05)	3.56 (0.99)
	Adjusted *B* [95% CI]	Ref	0.44 [0.29; 0.58]	Ref	0.14 [0.02; 0.27]
	p		<0.001		0.026
Limiting long-standing illness					
	n included in analysis	4129	209	3740	190
	% (*n*) reporting outcome	32.2 (1330)	55.0 (115)	36.2 (1354)	56.8 (108)
	Adjusted OR [95% CI]	1.00 (ref)	1.76 [1.29; 2.41]	1.00 (ref)	1.24 [0.85; 1.79]
	p		<0.001		0.268
Depressive symptoms					
	n included in analysis	4098	210	3643	188
	% (*n*) reporting outcome	11.9 (487)	28.6 (60)	10.8 (392)	29.8 (56)
	Adjusted OR [95% CI]	1.00 (ref)	2.01 [1.41; 2.85]	1.00 (ref)	2.37 [1.57; 3.60]
	p		<0.001		<0.001
Depressive symptoms (range 0-8)					
	n included in analysis	4098	210	3643	188
	Mean (SD) score	1.30 (1.78)	2.60 (2.42)	1.24 (1.73)	2.31 (2.12)
	Adjusted *B* [95% CI]	Ref	0.94 [0.68; 1.20]	Ref	0.38 [0.15; 0.62]
	P		<0.001		<0.001
Quality of life (range 0-57)					
	n included in analysis	3942	203	3106	154
	Mean (SD) score	41.72 (8.36)	34.24 (9.57)	42.19 (8.47)	36.56 (9.34)
	Adjusted *B* [95% CI]	Ref	-5.82 [-7.01; -4.62]	Ref	0.01 [-0.96; 0.97]
	p		<0.001		0.992
Life satisfaction (range 0-30)					
	n included in analysis	4008	199	3240	160
	Mean (SD) score	21.05 (6.02)	17.45 (7.62)	21.22 (6.03)	18.46 (7.31)
	Adjusted *B* [95% CI]	Ref	-2.36 [-3.25; -1.47]	Ref	-0.15 [-0.89; 0.59]
	p		<0.001		0.691

OR, odds ratio. CI, confidence interval. SD, standard deviation.
ORs, *B*s and 95% CIs are adjusted for age, sex, ethnicity, wealth, and body mass index. Prospective results are additionally adjusted for status/score on the outcome variable at baseline (Wave 5).
Ref = reference category.

For peer review only

For peer review only - http://bmjopen.bmj.com/site/about/guidelines.xhtml

We assessed whether prospective associations observed between perceived weight discrimination and self-rated health and depressive symptoms were mediated by stress exposure and health-risk behaviours (Figure 1; as limiting long-standing illness, quality of life, and life satisfaction were not prospectively associated with discrimination these were not investigated). After adjustment for covariates, participants who reported weight discrimination in Wave 5 had significantly higher hair cortisol concentrations (indicative of stress exposure) and higher odds of physically inactivity in Wave 6 than those who did not (Table 3; path *a* Figure 1). No association with smoking or alcohol consumption was observed (Table 3). Physical inactivity in Wave 6 was significantly associated with higher odds of fair/poor self-rated health and depressive symptoms in Wave 7 (Table 4; path *b* Figure 1). While hair cortisol concentrations in Wave 6 were not significantly associated with depressive symptoms in Wave 7 (Table 4; path *b* Figure 1), each one-unit increase in hair cortisol concentration (log pg/mg) was associated with 17% higher odds of subsequently reporting fair/poor self-rated health, which while not quite statistically significant (p=0.066) warranted further investigation as a potential mediator.

Table 3 Associations of perceived weight discrimination at Wave 5 with health behaviours and stress exposure at Wave 6 (path *a* on Figure 1)

		**No perceived weight discrimination**	**Perceived weight discrimination**
Smoking status			
	n included in analysis	4131	210
	% (*n*) current smoker	8.4 (347)	14.3 (30)
	Adjusted OR [95% CI]<br>*p*	1.00 (ref)	1.43 [0.91-2.25]<br>0.120
Physical activity			
	n included in analysis	4131	210
	% (*n*) physically inactive	5.9 (242)	13.8 (29)
	Adjusted OR [95% CI]<br>*p*	1.00 (ref)	1.90 [1.18; 3.05]<br>0.008
Alcohol consumption			
	n included in analysis	3890	186
	% (*n*) frequent alcohol consumption	20.6 (801)	12.9 (24)
	Adjusted OR [95% CI]<br>*p*	1.00 (ref)	0.93 [0.59; 1.49]<br>0.774
Hair cortisol concentration			
	n included in analysis	2456	128
	Mean (SD) log pg/mg	0.90 (0.57)	1.09 (0.65)
	Adjusted *B* [95% CI]<br>*p*	Ref	0.14 [0.03; 0.25]<br>0.012

OR, odds ratio. CI, confidence interval.
ORs and 95% CIs are adjusted for age, sex, ethnicity, wealth, and body mass index.

For peer review only - http://bmjopen.bmj.com/site/about/guidelines.xhtml

**Table 4** Associations of level of physical activity and stress exposure at Wave 6 with health and wellbeing outcomes (prospectively associated with perceived weight discrimination) at Wave 7 (path *b* on Figure 1)				
	**Fair/poor self-rated health**		**Depressive symptoms**	
	**No**	**Yes**	**No**	**Yes**
Physical activity				
n included in analysis	2821	1075	3404	456
% (*n*) physically inactive	3.4 (96)	12.4 (133)	5.1 (173)	11.4 (52)
Adjusted OR [95% CI]<br>*p*	1.00 (ref)	2.57 [1.92; 3.45]<br><0.001	1.00 (ref)	1.71 [1.21; 2.42]<br>0.002
Hair cortisol concentration				
n included in analysis	1699	623	2008	292
Mean (SD) log pg/mg	0.89 (0.57)	0.97 (0.58)	0.91 (0.57)	0.91 (0.57)
Adjusted OR [95% CI]<br>*p*	1.00 (ref)	1.17 [0.99; 1.38]<br>0.066	1.00 (ref)	0.93 [0.74; 1.17]<br>0.538

OR, odds ratio. CI, confidence interval.
ORs and 95% CIs are adjusted for age, sex, ethnicity, wealth, and body mass index.
Ref = reference category.

Results of mediation analyses are summarised in Table 5 (path *c*, path *c'*, and indirect effects Figure 1). There was no evidence of mediation, with effect ratios indicating that differences in physical inactivity explained approximately 4.2% of the association between perceived weight discrimination and self-rated health and 1.5% of the association between perceived weight discrimination and depressive symptoms, and differences in hair cortisol concentrations explained 1.5% of the association between perceived weight discrimination and self-rated health. None of these indirect effects were statistically significant.

For peer review only - http://bmjopen.bmj.com/site/about/guidelines.xhtml

Table 5 Models testing mediation of the associations between perceived weight discrimination and fair/poor self-rated health and depressive symptoms by level of physical activity and stress exposure (see Figure 1)

	Mediation by physical inactivity						Mediation by hair cortisol concentration				
	**Coeff.**	**SE**	***p****	**Bootstrap 95% CI**	**Effect ratio**		**Coeff.**	**SE**	***p****	**Bootstrap 95% CI**	**Effect ratio**
**Fair/poor self-rated health**											
Total effect (path *c*)	0.081	0.030	0.007	-	-		0.080	0.038	0.034	-	-
Direct effect (path *c'*)	0.077	0.030	0.009	-	-		0.079	0.038	0.037	-	-
Indirect effect (via mediator)	0.003	0.002	0.062	-0.0002; 0.009	0.042		0.001	0.878	0.380	-0.001; 0.005	0.015
**Depressive symptoms**											
Total effect (path *c*)	0.113	0.023	<0.001	-	-		-	-	-	-	-
Direct effect (path *c'*)	0.111	0.023	<0.001	-	-		-	-	-	-	-
Indirect effect (via mediator)	0.002	0.001	0.169	-0.001; 0.005	0.015		-	-	-	-	-

Analyses are adjusted for age, sex, ethnicity, wealth, body mass index, and status on the outcome variable at baseline (Wave 5).
Coeff. = coefficient; SE = standard error; CI = confidence interval; BMI = body mass index.
**p* values shown for indirect effects are derived from the Sobel test for consistency with total and direct effects, however bootstrap 95% confidence intervals provide a more robust indication of significant mediation (see Method for more details).

For peer review only - http://bmjopen.bmj.com/site/about/guidelines.xhtml

Sensitivity analyses

In the first sensitivity analysis, we expanded our sample to include participants who provided data on perceived weight discrimination but did not have a BMI in the overweight/obesity range (see Supplementary Table 1 for sample characteristics). We assessed whether the pattern of associations between weight discrimination and health and wellbeing outcomes was similar when including these participants (Supplementary Table 2). The results remained unchanged when including participants who did not have overweight/obesity.

For our second sensitivity analysis we changed the frequency weight discrimination was encountered from *at least once a year* (main analysis) to whether the participant *ever* experienced discrimination (*'never' vs all other options*). Ever encountering weight discrimination had a similar impact on cross-sectional and prospective health and wellbeing outcomes (Supplementary Table 3).

Discussion

In this large cohort of older adults, cross-sectionally, perceived weight discrimination was associated with higher odds of fair/poor self-rated health, limiting long-standing illness, and depressive symptoms, and lower quality of life and life satisfaction. Prospectively, perceived weight discrimination was associated with higher odds of fair/poor self-rated health and depressive symptoms after adjustment for baseline status. All associations were independent of age, sex, ethnicity, wealth, and BMI. However, while those who reported weight discrimination had higher odds of physically inactivity and higher hair cortisol concentrations than those who did not, there was no evidence that these variables mediated associations between perceived weight discrimination and poor health and wellbeing.

Our finding that perceived weight discrimination is associated with poorer health and wellbeing is consistent with previous studies (7–11). While our results provide prospective evidence linking perceived weight discrimination with self-rated health and depressive symptoms, as has been observed in other samples (7,8,11), we failed to replicate a prospective association with life satisfaction (7) or detect any prospective association with limiting long-standing illness or quality of life. It is unclear whether this is the result of differences in measurement (e.g., assessing limiting long-standing illness vs total disease burden), sample (e.g., age range), setting (i.e., England versus the US), or covariates used. Nonetheless, taking our

For peer review only - http://bmjopen.bmj.com/site/about/guidelines.xhtml

findings in the context of the literature, it is evident that weight stigma is a social determinant of health and a driver of health inequalities (40). As such, it is not only a social justice issue, but also a public health issue.

A key study aim was to explore the role of stress and health-risk behaviours as pathways underpinning associations between weight discrimination and health and wellbeing (26). In line with previous findings, perceived weight discrimination was associated with increased stress (higher hair cortisol concentrations) and physical inactivity (19,20), but these variables explained only a very small and non-significant proportion of the total associations of perceived weight discrimination with self-rated health and depressive symptoms. Associations with smoking and alcohol consumption were not statistically significant, so these variables were not considered plausible mediators. There are two potential explanations for these findings. First, the variables we assessed make only small contributions to the impact of weight discrimination on health and wellbeing, and there are other variables more important to this relationship that we were unable to account for in our modelling. We had no information on dietary factors so were unable to explore these as potential mediators. Given evidence that people who experience weight discrimination are at increased risk of overeating and other unhealthy eating behaviours (41,42), this may present an important unexamined pathway from weight discrimination to poor health. Another unmeasured variable was psychological distress, which could link perceived weight discrimination with health outcomes independently from the stress biomarker included in our analysis. A second explanation is that the variables we analysed as potential mediators were not adequately captured by the available measures. For example, hair cortisol was examined in 2cm long hair samples, and thus reflected approximately two months' exposure to circulating cortisol. While there are advantages in using hair cortisol over other more transient cortisol measures (e.g., in blood or saliva) two months is still a relatively short interval given that these data were collected on average two years after discrimination was assessed. Further it is possible that other stress-related biological pathways (such as chronic inflammation) could play a role in the weight discrimination-health relationship. Physical activity was self-reported and comparisons of self-reported versus objective measures of physical activity have shown that there are often substantial differences between what people say and what they actually do (43). Further research should aim to replicate our analyses with different measures of the constructs we have examined (stress, physical inactivity, smoking, and alcohol consumption) to establish whether the null results of our mediation analyses are the result of limitations in our measures (such as self-reported health risk behaviours) or an accurate representation of the causal pathways linking perceived weight discrimination with poorer health and wellbeing outcomes.

For peer review only - http://bmjopen.bmj.com/site/about/guidelines.xhtml

Strengths of the study include the large sample, prospective study design, and assessment of multiple mediators and outcomes. However, there were several limitations. Weight was not measured in the same data collection wave as discrimination, and participants may have changed weight status since experiencing discrimination. The prevalence of weight discrimination in the sample was low. We did not have complete data on weight, and it is possible that people most troubled by their weight were more likely to decline to be weighed. It is also possible that individuals most vulnerable to negative effects of weight discrimination opted not to complete the item on weight discrimination. The discrimination questions asked about five broad discriminatory situations and was not tailored for weight discrimination. Other measures with more specific items on experiences weight discrimination, for example having to pay more on public transport for occupying two passenger seats or being viewed unfavourably as a potential romantic partner, may have garnered different results. Finally, while the data were drawn from a representative sample, there was missing data and loss to follow-up, meaning the results may not be representative of the target population. In addition, as the sample comprised predominantly white middle-aged and older adults. The results may not generalise to other samples, in particular, younger samples who may be more likely to report weight discrimination (44).

Overall, these results provide further prospective evidence linking weight-based discrimination with poorer health and wellbeing. However, while perceived weight discrimination was associated with greater stress exposure and physical inactivity, these variables explained only a small part of the association between weight discrimination and poorer health and wellbeing. This leaves unanswered questions about the pathways through which weight discrimination adversely impacts health and wellbeing. Further research into mechanisms of action is required if policies and interventions to mitigate the health burden of weight discrimination are to be developed. Beyond this, the literature underscores the need to tackle weight discrimination on a societal level, if not for moral reasons, then as a strategy to improve population health.

For peer review only

Declarations

Contributor statement: RAH conducted statistical analyses, wrote, edited and reviewed the manuscript. SEJ conducted statistical analyses, wrote, edited and reviewed the manuscript. EC edited and reviewed the manuscript. AS, edited and reviewed the manuscript.

Competing interests: None

Funding: This research was supported by the Economic and Social Research Council (https://esrc.ukri.org/), grant number ES/R005990/1(awarded to AS and SEJ), and the Academy of Medical Sciences/the Wellcome Trust/the Government Department of Business, Energy and Industrial Strategy/the British Heart Foundation/Diabetes UK Springboard Award (SBF006\1036) (awarded to RH). The funders had no role in study design, data collection and analysis, decision to publish, or preparation of the manuscript.

Data sharing: Data from the English Longitudinal Study of Ageing are available to download from g2aging.org.

Ethics approval statement: Ethical approval for ELSA was obtained from the London Multicentre Research and Ethics Committee (MREC/01/02/91). All participants provided informed consent.

For peer review only

For peer review only - http://bmjopen.bmj.com/site/about/guidelines.xhtml

References

1. Adults' average BMI [Internet]. Health Survey for England. 2019 [cited 2020 Mar 19]. Available from: http://healthsurvey.hscic.gov.uk/data-visualisation/data-visualisation/explore-the-trends/weight/adult/bmi.aspx

2. NHS Digital. Statistics on Obesity, Physical Activity and Diet, England, 2019 [Internet]. 2019 [cited 2020 Mar 19]. Available from: https://digital.nhs.uk/data-and-information/publications/statistical/statistics-on-obesity-physical-activity-and-diet/statistics-on-obesity-physical-activity-and-diet-england-2019/part-3-adult-obesity

3. Wardle J, Boniface D. Changes in the distributions of body mass index and waist circumference in English adults, 1993/1994 to 2002/2003. Int J Obes. 2008;32(3):527–32.

4. Puhl RM, Heuer CA. The Stigma of Obesity: A Review and Update. Obesity. 2009 May 1;17(5):941–64.

5. Emmer C, Bosnjak M, Mata J. The association between weight stigma and mental health: A meta-analysis. Obes Rev. 2020;21(1):e12935.

6. Puhl R, Suh Y. Health Consequences of Weight Stigma: Implications for Obesity Prevention and Treatment. Curr Obes Rep. 2015 Apr 1;4(2):182–90.

7. Sutin AR, Stephan Y, Carretta H, Terracciano A. Perceived discrimination and physical, cognitive, and emotional health in older adulthood. Am J Geriatr Psychiatry Off J Am Assoc Geriatr Psychiatry. 2015 Feb;23(2):171–9.

8. Hatzenbuehler ML, Keyes KM, Hasin DS. Associations between perceived weight discrimination and the prevalence of psychiatric disorders in the general population. Obes Silver Spring Md. 2009 Nov;17(11):2033–9.

9. Hackman J, Maupin J, Brewis AA. Weight-related stigma is a significant psychosocial stressor in developing countries: Evidence from Guatemala. Soc Sci Med 1982. 2016;161:55–60.

10. Sutin AR, Stephan Y, Grzywacz JG, Robinson E, Daly M, Terracciano A. Perceived weight discrimination, changes in health, and daily stressors. Obesity. 2016 Oct 1;24(10):2202–9.

11. Schafer MH, Ferraro KF. The stigma of obesity: does perceived weight discrimination affect identity and physical health? Soc Psychol Q. 2011;74(1):76–97.

12. Sutin AR, Terracciano A. Sources of weight discrimination and health. Stigma Health. 2017;2(1):23–7.

13. Sutin AR, Stephan Y, Terracciano A. Weight Discrimination and Risk of Mortality. Psychol Sci. 2015 Nov 1;26(11):1803–11.

14. Tomiyama AJ. Weight stigma is stressful. A review of evidence for the Cyclic Obesity/Weight-Based Stigma model. Appetite. 2014 Nov;82:8–15.

For peer review only - http://bmjopen.bmj.com/site/about/guidelines.xhtml

15. Schvey NA, Puhl RM, Brownell KD. The stress of stigma: exploring the effect of weight stigma on cortisol reactivity. Psychosom Med. 2014 Feb;76(2):156–62.

16. Himmelstein MS, Incollingo Belsky AC, Tomiyama AJ. The weight of stigma: cortisol reactivity to manipulated weight stigma. Obes Silver Spring Md. 2015 Feb;23(2):368–74.

17. Puhl RM, Brownell KD. Confronting and coping with weight stigma: an investigation of overweight and obese adults. Obes Silver Spring Md. 2006 Oct;14(10):1802–15.

18. McEwen BS, Gianaros PJ. Central role of the brain in stress and adaptation: links to socioeconomic status, health, and disease. Ann N Y Acad Sci. 2010 Feb;1186:190–222.

19. Jackson SE, Kirschbaum C, Steptoe A. Perceived weight discrimination and chronic biochemical stress: A population-based study using cortisol in scalp hair. Obes Silver Spring Md. 2016 Dec;24(12):2515–21.

20. Tomiyama AJ, Epel ES, McClatchey TM, Poelke G, Kemeny ME, McCoy SK, et al. Associations of weight stigma with cortisol and oxidative stress independent of adiposity. Health Psychol Off J Div Health Psychol Am Psychol Assoc. 2014 Aug;33(8):862–7.

21. Born JM, Lemmens SGT, Rutters F, Nieuwenhuizen AG, Formisano E, Goebel R, et al. Acute stress and food-related reward activation in the brain during food choice during eating in the absence of hunger. Int J Obes. 2010;34(1):172–81.

22. Groesz LM, McCoy S, Carl J, Saslow L, Stewart J, Adler N, et al. What is eating you? Stress and the drive to eat. Appetite. 2012 Apr;58(2):717–21.

23. Kandiah J, Yake M, Jones J, Meyer M. Stress influences appetite and comfort food preferences in college women. Nutr Res. 2006;26(3):118–23.

24. Vucetic Z, Reyes TM. Central dopaminergic circuitry controlling food intake and reward: implications for the regulation of obesity. Wiley Interdiscip Rev Syst Biol Med. 2010 Sep 1;2(5):577–93.

25. Bethancourt HJ, Rosenberg DE, Beatty T, Arterburn DE. Barriers to and Facilitators of Physical Activity Program Use Among Older Adults. Clin Med Res. 2014 Jan 10;cmr.2013.1171.

26. Pascoe EA, Smart Richman L. Perceived discrimination and health: A meta-analytic review. Psychol Bull. 2009;135(4):531–54.

27. Steptoe A, Breeze E, Banks J, Nazroo J. Cohort profile: the English Longitudinal Study of Ageing. Int J Epidemiol. 2013 Dec;42(6):1640–8.

28. Jackson SE, Hackett RA, Steptoe A. Associations between age discrimination and health and wellbeing: cross-sectional and prospective analysis of the English Longitudinal Study of Ageing. Lancet Public Health. 2019 Apr 1;4(4):e200–8.

29. Jackson SE, Beeken RJ, Wardle J. Obesity, perceived weight discrimination, and psychological well-being in older adults in England. Obesity. 2015 May 1;23(5):1105–11.

For peer review only - http://bmjopen.bmj.com/site/about/guidelines.xhtml

30. Jackson SE, Steptoe A, Beeken RJ, Croker H, Wardle J. Perceived weight discrimination in England: a population-based study of adults aged ⩾50 years. Int J Obes. 2015 May;39(5):858–64.

31. Jackson SE, Beeken RJ, Wardle J. Perceived weight discrimination and changes in weight, waist circumference, and weight status. Obesity. 2014 Dec 1;22(12):2485–8.

32. Jackson SE, Steptoe A. Association between perceived weight discrimination and physical activity: a population-based study among English middle-aged and older adults. BMJ Open. 2017 Mar 1;7(3):e014592.

33. Jackson SE, Steptoe A. Obesity, perceived weight discrimination, and hair cortisol: a population-based study. Psychoneuroendocrinology. 2018 Dec 1;98:67–73.

34. Steptoe A, Jackson SE. The Life Skills of Older Americans: Association with Economic, Psychological, Social, and Health Outcomes. Sci Rep. 2018 Jul 5;8(1):9669.

35. Radloff LS. The CES-D scale. Appl Psychol Meas. 1977;1(3):385–401.

36. Steffick DE. Documentation of affective functioning measures in the Health and Retirement Study. HRS Documentation Report DR-005; 2000.

37. Hyde M, Wiggins RD, Higgs P, Blane DB. A measure of quality of life in early old age: the theory, development and properties of a needs satisfaction model (CASP-19). Aging Ment Health. 2003;7(3):186–94.

38. Diener E, Emmons RA, Larsen RJ, Griffin S. The satisfaction with life scale. J Assess. 1985;49(1):71–5.

39. Jackson SE, Kirschbaum C, Steptoe A. Hair cortisol and adiposity in a population-based sample of 2,527 men and women aged 54 to 87 years. Obesity. 2017;25(3):539–44.

40. Hatzenbuehler ML, Phelan JC, Link BG. Stigma as a Fundamental Cause of Population Health Inequalities. Am J Public Health. 2013 May;103(5):813–21.

41. Puhl RM, Moss-Racusin CA, Schwartz MB. Internalization of weight bias: Implications for binge eating and emotional well-being. Obes Silver Spring Md. 2007 Jan;15(1):19–23.

42. Sutin A, Robinson E, Daly M, Terracciano A. Weight discrimination and unhealthy eating-related behaviors. Appetite. 2016 01;102:83–9.

43. Kapteyn A, Banks J, Hamer M, Smith JP, Steptoe A, Soest A van, et al. What they say and what they do: comparing physical activity across the USA, England and the Netherlands. J Epidemiol Community Health. 2018 Jun 1;72(6):471–6.

44. Puhl RM, Andreyeva T, Brownell KD. Perceptions of weight discrimination: prevalence and comparison to race and gender discrimination in America. Int J Obes. 2008 Jun;32(6):992–1000.

For peer review only

For peer review only - http://bmjopen.bmj.com/site/about/guidelines.xhtml

Figures legends

Figure 1. Mediation model of associations between perceived weight discrimination and health and wellbeing outcomes via health behaviours and stress exposure

For peer review only

For peer review only - http://bmjopen.bmj.com/site/about/guidelines.xhtml

Figures

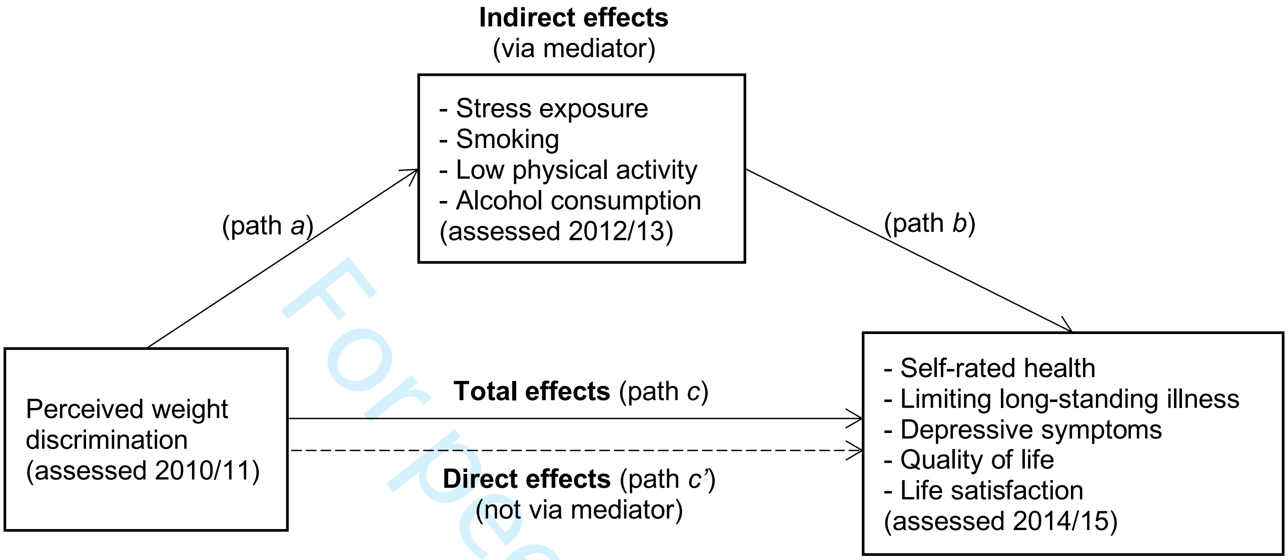

Figure 1. Mediation model of associations between perceived weight discrimination and health and wellbeing outcomes via health behaviours and stress exposure

For peer review only

Sensitivity analyses - Tables

**Supplementary Table 1** Sample characteristics at baseline (Wave 5) **all weight categories**					
		**Whole sample (*n*=5965)**	**Perceived weight discrimination (*n*=219)**	**No perceived weight discrimination (*n*=5746)**	***p***
Age (years), mean (SD)		66.56 (8.13)	62.16 (6.74)	66.72 (8.13)	<0.001
Sex, % (*n*)					
	Men	44.5 (2655)	35.2 (77)	44.9 (2578)	=0.005
	Women	55.5 (3310)	64.8 (142)	55.1 (3168)	-
Ethnicity, % (*n*)					
	White	97.9 (5841)	97.7 (214)	97.9 (5627)	0.829
	Other	2.1 (124)	2.3 (5)	2.1 (199)	-
Wealth quintile, % (*n*)					
	1 (poorest)	14.6 (868)	26.0 (57)	14.1 (811)	<0.001
	2	19.8 (1183)	30.6 (67)	19.4 (1116)	-
	3	19.9 (1190)	18.3 (40)	20.0 (1150)	-
	4	21.9 (1306)	15.5 (34)	22.1 (1272)	-
	5 (richest)	23.8 (1418)	9.6 (21)	24.3 (1397)	-
BMI (kg/m^2), mean (SD)		28.27 (5.22)	36.05 (6.69)	27.98 (4.92)	<0.001
BMI, body mass index.					

For peer review only

Supplementary Table 2 Cross-sectional and prospective associations of perceived weight discrimination with health and wellbeing outcomes across **all weight categories**

	Cross-sectional (Wave 5)		Prospective (Wave 7)	
	**No perceived weight discrimination**	**Perceived weight discrimination**	**No perceived weight discrimination**	**Perceived weight discrimination**
Fair/poor self-rated health				
n included in analysis	5754	217	5122	197
% (*n*) reporting outcome	20.8 (1197)	48.4 (105)	24.4 (1251)	52.8 (104)
Adjusted OR [95% CI]<br>*p*	1.00 (ref)	2.32 [1.71; 3.16]<br><0.001	1.00 (ref)	1.66 [1.15; 2.42]<br>0.008
Limiting long-standing illness				
n included in analysis	5743	218	5172	199
% (*n*) reporting outcome	30.3 (1741)	55.0 (120)	33.9 (1755)	56.3 (112)
Adjusted OR [95% CI]<br>*p*	1.00 (ref)	1.91 [1.42; 2.58]<br><0.001	1.00 (ref)	1.32 [0.92; 1.88]<br>0.130
Depressive symptoms				
n included in analysis	5695	219	5027	197
% (*n*) reporting outcome	11.7 (668)	27.9 (61)	10.4 (525)	30.5 (60)
Adjusted OR [95% CI]<br>*p*	1.00 (ref)	2.05 [1.46; 2.89]<br><0.001	1.00 (ref)	2.74 [1.84; 4.09]<br><0.001
Quality of life (range 0-57)				
n included in analysis	5497	212	4318	162
Mean (SD) score	41.96 (8.34)	34.26 (9.55)	42.54 (8.38)	36.60 (9.40)
Adjusted *B* [95% CI]<br>*p*	Ref	-6.03 [-7.18; -4.88]<br><0.001	Ref	-0.21 [-1.12; 0.70]<br>0.651
Life satisfaction (range 0-30)				
n included in analysis	5577	207	4490	167
Mean (SD) score	21.07 (6.08)	17.49 (7.60)	21.28 (6.01)	18.32 (7.32)
Adjusted *B* [95% CI]<br>*p*	Ref	-2.50 [-3.37; -1.63]<br><0.001	Ref	-0.45 [-1.16; 0.26]<br>0.213

OR, odds ratio. CI, confidence interval. SD, standard deviation.
ORs, !s and 95% CIs are adjusted for age, sex, ethnicity, wealth, and body mass index. Prospective results are additionally adjusted for status/score on the outcome variable at baseline (Wave 5).
Ref = reference category.

For peer review only

Supplementary Table 3 Cross-sectional and prospective associations of perceived weight discrimination in **any domain (ever vs never)** with health and wellbeing outcomes **in overweight and obese participants**

	**Cross-sectional (Wave 5)**		**Prospective (Wave 7)**	
	**No perceived weight discrimination**	**Perceived weight discrimination**	**No perceived weight discrimination**	**Perceived weight discrimination**
Fair/poor self-rated health				
n included in analysis	4090	248	3670	223
% (*n*) reporting outcome	22.5 (919)	44.0 (109)	26.2 (960)	51.1 (114)
Adjusted OR [95% CI]	1.00 (ref)	1.74 [1.29; 2.35]	1.00 (ref)	1.68 [1.17; 2.41]
p		<0.001		0.005
Limiting long-standing illness				
n included in analysis	4089	249	3705	225
% (*n*) reporting outcome	32.0 (1309)	54.6 (136)	36.0 (1333)	57.3 (129)
Adjusted OR [95% CI]	1.00 (ref)	1.79 [1.34; 2.39]	1.00 (ref)	1.33 [0.94; 1.89]
p		<0.001		0.105
Depressive symptoms				
n included in analysis	4058	250	3609	222
% (*n*) reporting outcome	11.9 (483)	25.6 (64)	10.7 (385)	28.4 (63)
Adjusted OR [95% CI]	1.00 (ref)	1.75 [1.24; 2.45]	1.00 (ref)	2.54 [1.71; 3.77]
p		<0.001		<0.001
Quality of life (range 0-57)				
n included in analysis	3902	243	3080	180
Mean (SD) score	41.76 (8.35)	34.85 (9.50)	42.20 (8.47)	37.17 (9.39)
Adjusted *B* [95% CI]	Ref	-5.53 [-6.64; -4.42]	Ref	0.24 [-0.67; 1.15]
p		<0.001		0.608
Life satisfaction (range 0-30)				
n included in analysis	3968	239	3210	190
Mean (SD) score	21.05 (6.02)	18.05 (7.41)	21.22 (6.04)	18.89 (7.09)
Adjusted *B* [95% CI]	Ref	-1.87 [-2.70; -1.04]	Ref	-0.07 [-0.76; 0.62]
p		<0.001		0.844

OR, odds ratio. CI, confidence interval. SD, standard deviation.
ORs, !s and 95% CIs are adjusted for age, sex, ethnicity, wealth, and body mass index. Prospective results are additionally adjusted for status/score on the outcome variable at baseline (Wave 5).
Ref = reference category.

For peer review only

STROBE 2007 (v4) Statement—Checklist of items that should be included in reports of *cohort studies*

Section/Topic	Item #	Recommendation	Reported on page #
**Title and abstract**	1	(*a*) Indicate the study's design with a commonly used term in the title or the abstract	1,2
		(*b*) Provide in the abstract an informative and balanced summary of what was done and what was found	2
**Introduction**			
Background/rationale	2	Explain the scientific background and rationale for the investigation being reported	3-4
Objectives	3	State specific objectives, including any prespecified hypotheses	5
**Methods**			
Study design	4	Present key elements of study design early in the paper	5
Setting	5	Describe the setting, locations, and relevant dates, including periods of recruitment, exposure, follow-up, and data collection	5
Participants	6	(*a*) Give the eligibility criteria, and the sources and methods of selection of participants. Describe methods of follow-up	5
		(*b*) For matched studies, give matching criteria and number of exposed and unexposed	
Variables	7	Clearly define all outcomes, exposures, predictors, potential confounders, and effect modifiers. Give diagnostic criteria, if applicable	5-8
Data sources/ measurement	8*	For each variable of interest, give sources of data and details of methods of assessment (measurement). Describe comparability of assessment methods if there is more than one group	5-8
Bias	9	Describe any efforts to address potential sources of bias	
Study size	10	Explain how the study size was arrived at	5 & 9
Quantitative variables	11	Explain how quantitative variables were handled in the analyses. If applicable, describe which groupings were chosen and why	8
Statistical methods	12	(*a*) Describe all statistical methods, including those used to control for confounding	8
		(*b*) Describe any methods used to examine subgroups and interactions	
		(*c*) Explain how missing data were addressed	9
		(*d*) If applicable, explain how loss to follow-up was addressed	
		(*e*) Describe any sensitivity analyses	8-9
**Results**			

For peer review only

Participants	13*	(a) Report numbers of individuals at each stage of study—eg numbers potentially eligible, examined for eligibility, confirmed eligible, included in the study, completing follow-up, and analysed	9
		(b) Give reasons for non-participation at each stage	9
		(c) Consider use of a flow diagram	
Descriptive data	14*	(a) Give characteristics of study participants (eg demographic, clinical, social) and information on exposures and potential confounders	9
		(b) Indicate number of participants with missing data for each variable of interest	9
		(c) Summarise follow-up time (eg, average and total amount)	
Outcome data	15*	Report numbers of outcome events or summary measures over time	10 and Table 1
Main results	16	(*a*) Give unadjusted estimates and, if applicable, confounder-adjusted estimates and their precision (eg, 95% confidence interval). Make clear which confounders were adjusted for and why they were included	10 and Tables 2-5
		(*b*) Report category boundaries when continuous variables were categorized	6-7
		(*c*) If relevant, consider translating estimates of relative risk into absolute risk for a meaningful time period	
Other analyses	17	Report other analyses done—eg analyses of subgroups and interactions, and sensitivity analyses	10
**Discussion**			
Key results	18	Summarise key results with reference to study objectives	10
**Limitations**			
Interpretation	20	Give a cautious overall interpretation of results considering objectives, limitations, multiplicity of analyses, results from similar studies, and other relevant evidence	11-12
Generalisability	21	Discuss the generalisability (external validity) of the study results	12
**Other information**			
Funding	22	Give the source of funding and the role of the funders for the present study and, if applicable, for the original study on which the present article is based	17

*Give information separately for cases and controls in case-control studies and, if applicable, for exposed and unexposed groups in cohort and cross-sectional studies.

Note: An Explanation and Elaboration article discusses each checklist item and gives methodological background and published examples of transparent reporting. The STROBE checklist is best used in conjunction with this article (freely available on the Web sites of PLoS Medicine at http://www.plosmedicine.org/, Annals of Internal Medicine at http://www.annals.org/, and Epidemiology at http://www.epidem.com/). Information on the STROBE Initiative is available at www.strobe-statement.org.

For peer review only

The role of stress and health behaviour in linking weight discrimination and health: a secondary data analysis in England

Journal:	*BMJ Open*
Manuscript ID	bmjopen-2023-072043.R1
Article Type:	Original research
Date Submitted by the Author:	19-May-2023

Complete List of Authors:	Hackett, Ruth; King’s College London, Department of Psychology; University College London, Department of Behavioural Science and Health<br>Jackson, Sarah ; University College London, Epidemiology and Public Health<br>Corker, Elizabeth; University College London, Clinical; University of Sheffield, Department of Psychology<br>Steptoe, Andrew; University College London, Department of Behavioural Science and Health

**Primary Subject Heading**:	Epidemiology
Secondary Subject Heading:	Public health
Keywords:	EPIDEMIOLOGY, PUBLIC HEALTH, Depression & mood disorders < PSYCHIATRY

SCHOLARONE™
Manuscripts

I, the Submitting Author has the right to grant and does grant on behalf of all authors of the Work (as defined in the below author licence), an exclusive licence and/or a non-exclusive licence for contributions from authors who are: i) UK Crown employees; ii) where BMJ has agreed a CC-BY licence shall apply, and/or iii) in accordance with the terms applicable for US Federal Government officers or employees acting as part of their official duties; on a worldwide, perpetual, irrevocable, royalty-free basis to BMJ Publishing Group Ltd ("BMJ") its licensees and where the relevant Journal is co-owned by BMJ to the co-owners of the Journal, to publish the Work in this journal and any other BMJ products and to exploit all rights, as set out in our licence.

The Submitting Author accepts and understands that any supply made under these terms is made by BMJ to the Submitting Author unless you are acting as an employee on behalf of your employer or a postgraduate student of an affiliated institution which is paying any applicable article publishing charge ("APC") for Open Access articles. Where the Submitting Author wishes to make the Work available on an Open Access basis (and intends to pay the relevant APC), the terms of reuse of such Open Access shall be governed by a Creative Commons licence – details of these licences and which Creative Commons licence will apply to this Work are set out in our licence referred to above.

Other than as permitted in any relevant BMJ Author's Self Archiving Policies, I confirm this Work has not been accepted for publication elsewhere, is not being considered for publication elsewhere and does not duplicate material already published. I confirm all authors consent to publication of this Work and authorise the granting of this licence.

For peer review only

The role of stress and health behaviour in linking weight discrimination and health: a secondary data analysis in England

Ruth A. Hackett[1,2], Sarah E. Jackson[2], Elizabeth Corker[3,4], Andrew Steptoe[2]

[1] Health Psychology Section, Department of Psychology, Institute of Psychiatry, Psychology and Neuroscience, King's College London, London, UK

[2] Department of Behavioural Science and Health, University College London, London, UK

[3] Clinical, Educational and Health Psychology, University College London, London, UK

[4] Clinical and Applied Psychology Unit, Department of Psychology, University of Sheffield, Sheffield, UK

Running head: Weight discrimination, health and wellbeing

Corresponding author: Dr Ruth A Hackett, Health Psychology Section, Department of Psychology, Institute of Psychiatry, Psychology and Neuroscience, King's College London, Great Maze Pond, London SE1 9RT, UK. ruth.hackett@kcl.ac.uk +44 (0) 207 188 5410

Competing interests: None

Word count: 4,396

For peer review only - http://bmjopen.bmj.com/site/about/guidelines.xhtml

Abstract

Objective: To examine the role of stress and health-risk behaviours in relationships between weight discrimination and health and wellbeing.

Design: Secondary data analysis of an observational cohort study

Setting: The English Longitudinal Study of Ageing

Participants: Data were from 4,341 adults (≥50y) with overweight/obesity.

Primary Outcome Measures: We tested associations between perceived weight discrimination at baseline (2010/11) and self-rated health, limiting long-standing illness, depressive symptoms, quality of life, and life satisfaction over four-year follow-up (2010/11; 2014/15). Potential mediation by stress exposure (hair cortisol) and health-risk behaviours (smoking, physical inactivity, alcohol consumption) was assessed.

Results: Cross-sectionally, perceived weight discrimination was associated with higher odds of fair/poor self-rated health (OR=2.05[95%CI 1.49;2.82]), limiting long-standing illness (OR=1.76[1.29;2.41]) and depressive symptoms (OR=2.01[1.41;2.85]), and lower quality of life (*B*=-5.82[95%CI -7.01;-4.62]) and life satisfaction (*B*=-2.36[-3.25;-1.47]). Prospectively, weight discrimination was associated with higher odds of fair/poor self-rated health (OR=1.63[1.10;2.40]) and depressive symptoms (OR=2.37[95%CI 1.57;3.60]) adjusting for baseline status. Those who reported discrimination had higher hair cortisol concentrations (*B*=0.14[0.03;0.25]) and higher odds of physical inactivity (OR=1.90[1.18;3.05]). These variables did not significantly mediate associations between discrimination and health outcomes.

Conclusions: Weight discrimination is associated with poor health and wellbeing. While this discrimination is associated with stress exposure and physical inactivity, these variables explain little of the association between discrimination and poorer outcomes.

Key words: weight discrimination; stigma; obesity; physical activity; smoking; health; wellbeing

For peer review only - http://bmjopen.bmj.com/site/about/guidelines.xhtml

Strengths and limitations

- Secondary data analysis of a large prospective observational study.
- Assessment of multiple health and wellbeing outcomes.
- The prevalence of weight discrimination in the sample was low (which limits generalisability) and it was only assessed on one occasion increasing the risk of misclassification bias.
- Measures of health behaviour were self-reported increasing the risk of measurement error and there was no data on diet (pattens or quality) or eating behaviour (e.g., dieting, disordered eating)
- The sample was compromised of mostly white middle aged and older adults which limits generalisability.

For peer review only

Introduction

The average person in the United Kingdom (UK) carries excess weight (1,2). Recent decades have seen an increase in the proportion of adults with a body mass index (BMI) in the overweight (25-29.9kg/m^2) or obesity (≥30kg/m^2) range, and a shift in the distribution of cases such that a greater proportion have 'morbid' obesity (BMI ≥40kg/m^2) (3). Despite the increasingly normative nature of obesity, it is widely stigmatised with those with obesity characterised as lazy, unintelligent, and lacking in self-discipline (4). These stereotypes translate into weight discrimination across contexts and interactions, including the workplace, education, healthcare settings, and interpersonal relationships (4).

Mounting evidence suggests that weight discrimination can impact health and wellbeing (5,6). Studies have linked perceived weight discrimination to adverse outcomes, including poorer self-rated health, greater disease burden, functional disability, psychiatric comorbidity, psychosocial stress, loneliness, poorer life satisfaction, and increased risk of premature death (7–13). Evidence is suggestive of a prospective relationship (i.e. discrimination preceding a decline in health) which persists after adjustment for BMI (7,9). It is of interest to analyse these issues in middle-aged and older people, among whom disease and disability risk is elevated.

Several mechanisms may underpin the relationship between perceived weight discrimination and poorer health and wellbeing. Prominent theories focus on the role of (i) stress responses to discrimination and (ii) health-risk behaviours as a means of coping with or avoiding discrimination (14). Experiencing weight discrimination can be stressful, both physiologically, in the form of cortisol responses (15,16), and psychologically, in the form of increased negative emotion (e.g. psychological distress, depressive symptoms, and fear, among others) (8). If an individual perceives discrimination regularly, as is common with weight stigma (17), stress responses will be activated frequently, potentially leading to chronically elevated cortisol and a negative emotional state (e.g., depression, anxiety, ongoing feelings of stress etc.) . This in turn lead to ill health under allostatic load theory (18). Indeed, observational studies have associated elevated cortisol levels and weight discrimination (19,20).

Health-risk behaviours may also link weight discrimination to poorer health and wellbeing. These may emerge as a biological response to discrimination, for example, hypothalamic-pituitary-adrenal (HPA) axis activation upregulating appetite and the drive to consume energy-dense 'comfort' foods (21–23). Indeed, disordered eating and binge eating disorder (eating large amounts accompanied by feelings of distress) is common at higher weight ranges (24) and has been associated with weight discrimination (25). In this way, weight stigma

For peer review only - http://bmjopen.bmj.com/site/about/guidelines.xhtml

may contribute to a vicious cycle of stress, weight gain and further discrimination (14). Unhealthy behaviours may also provide a coping mechanism: food, nicotine, and alcohol activate dopaminergic reward pathways in the brain (26) and may help to alleviate the negative psychological impact of discrimination. Avoiding activities for fear of discrimination (e.g. exercising in public places) may also act as a barrier to a healthy lifestyle (27). To our knowledge, no studies have investigated the extent to which these variables mediate associations between weight-based discrimination and health and wellbeing outcomes.

The present study used data from English Longitudinal Study of Ageing (ELSA) to examine (i) cross-sectional and prospective associations between perceived weight discrimination and health and wellbeing (self-rated health, limiting long-standing illness, depressive symptoms, quality of life, and life satisfaction) and (ii) whether these associations were mediated by stress exposure and health-risk behaviours (smoking, physical inactivity, and alcohol consumption).

Methods

Study population

ELSA is a representative longitudinal study of adults aged ≥50 living in England (28). Established in 2002, ELSA collects data via computer-assisted personal interviews and self-completion questionnaires biennially, with nurse visits in alternate waves to collect objective health measures, including BMI.

We used data on perceived weight discrimination from Wave 5 (2010/11; the only wave in which discrimination was assessed); hair cortisol, health behaviours, and BMI from Wave 6 (2012/13), and health and wellbeing from Waves 5 and 7 (2014/15). We restricted our sample to those with a BMI in the overweight or obese range (≥25kg/m^2).

Ethical approval for ELSA was obtained from the London Multicentre Research and Ethics Committee (MREC/01/02/91). All participants provided informed consent.

Patient and public involvement

No patient involved.

For peer review only - http://bmjopen.bmj.com/site/about/guidelines.xhtml

Measures

All questionnaire measures used in the study can be found in Supplementary material.

Exposure: perceived weight discrimination

Our measure of perceived discrimination was based on items developed and used in other prospective cohort studies (e.g. Health and Retirement Study; Midlife Development in the United States) (29). Participants were asked how often they encounter five discriminatory situations: *"In your day-to-day life, how often have any of the following things happened to you: 1) you are treated with less respect or courtesy; 2) you receive poorer service than other people in restaurants and stores; 3) people act as if they think you are not clever; 4) you are threatened or harassed; and 5) you receive poorer service or treatment than other people from doctors or hospitals."*. Response options were on a 5-point scale ranging from "*never*" to "*almost every day*." Because data were skewed, with most participants reporting no discrimination, we dichotomised responses to indicate whether or not participants had experienced discrimination in the past year (a few times or more a year vs. less than once a year or never), with the exception of the fifth item which was dichotomised to indicate whether or not respondents had ever experienced discrimination from doctors or hospitals (never vs. all other options) as most participants never reported discrimination in this setting (30–33).

A follow-up question asked participants who reported discrimination to indicate the reason(s) they attributed to their experience (e.g., weight, age, sex, race, sexual orientation, disability etc.,) (30–33). Those who attributed any experience of discrimination to their weight are treated as cases of perceived weight discrimination as in other studies (19,34–38).

Outcomes: health and wellbeing

Self-rated health was assessed using a single item: *"Would you say your health is… poor/fair/good/very good/excellent?"* We analysed the proportion of individuals rating their health as fair/poor, as in other investigations (30,39). As a sensitivity check, analyses were also conducted using the continuous range of scores.

Limiting long-standing illness was assessed with two questions: 1) "*Do you have any long-standing illness, disability, or infirmity? By long-standing I mean anything that has troubled you over a period of time or that is likely to affect you over a period of time*." Those who responded yes were asked: 2) "*Does this illness or*

For peer review only - http://bmjopen.bmj.com/site/about/guidelines.xhtml

disability limit your activities in any way?" Those who responded yes to both items were classed as having a limiting long-standing illness.

Depressive symptoms were assessed with the eight-item Center for Epidemiologic Studies Depression Scale (40). Respondents were asked if they had experienced depressive symptoms (e.g. restless sleep and being unhappy) over the past month using a binary (yes/no) response. Total scores ranged from 0 to 8. Data were dichotomised using an established cut-off (scores ≥4) (41). As a sensitivity check, analyses were also conducted using the continuous range of scores.

Quality of life was assessed with the CASP-19 (42). Items covered four domains of quality of life; control (e.g., '*I feel that what happens to me is out of my control*'), autonomy (e.g., '*My health stops me from doing things I want to do*'), self-realisation (e.g., '*I feel that life is full of opportunities*'), and pleasure (e.g., '*I enjoy being in the company of others*'). Respondents were asked how often each statement applies to them (often=0, sometimes=1, not often=2, never=3). Positively-worded items were reverse scored so that a higher total score indicated higher quality of life (range: 0–57).

Life satisfaction was assessed with the 5-item Satisfaction With Life Scale (43), which asks the extent to which participants agree with statements including '*In most ways my life is close to my ideal*'; 'and '*I am satisfied with my life*';. Responses ranged from 0 (strongly disagree) to 6 (strongly agree) and were summed to produce a total score of 0-30.

Mediators: stress exposure and health-risk behaviours

The hair cortisol assessment procedure has been described in detail elsewhere (44). Briefly, a scalp-proximal hair sample at least 2cm long and weighing at least 10mg was taken from the posterior vertex, cut as close to the scalp as possible. The wash procedure and steroid extraction were undertaken using high performance liquid chromatography–mass spectrometry. Assuming an average hair growth of 1cm per month the 2cm hair segment represents average cortisol accumulated two months prior to sampling.

Smoking status was assessed with the question "*Do you smoke cigarettes at all nowadays?*" (yes/no).

Physical activity was self-reported in response to three questions on the frequency of participation in light, moderate and vigorous activities (more than once a week/once a week/one to three times a month/hardly ever or never). We compared participants who were physically inactive (defined as no activity on a weekly basis) with those who reported engaging in light, moderate, or vigorous activity at least once a week.

For peer review only - http://bmjopen.bmj.com/site/about/guidelines.xhtml

Alcohol consumption was assessed with the question "*On how many days out of the last seven did you have an alcoholic drink?*". Those who responded 'five', 'six' or 'seven' days were classified as frequent alcohol drinkers. We compared frequent alcohol drinkers with all other categories combined.

Covariates

Participants self-reported their age, sex, and ethnicity (white, other). Socioeconomic status was indexed with a measure of household non-pension wealth (28)), divided into quintiles across the entire Wave 5 sample. BMI (m^2/kg) was based on objective height and weight measurements.

Statistical analysis

Associations between perceived weight discrimination and covariates were examined using one-way independent analysis of variance (ANOVA) for continuous variables and χ^2 tests for categorical variables.

For our primary analyses, we used multivariable regression models (logistic for categorical outcomes, linear for continuous outcomes) to explore cross-sectional and prospective associations between perceived weight discrimination and health and wellbeing. All models were adjusted for age, sex, ethnicity, wealth, and BMI. Prospective models were additionally adjusted for baseline status/score on the outcome variable.

Where perceived weight discrimination was significantly associated with an outcome in prospective analyses, we tested for mediation by stress exposure and health-risk behaviours (Figure 1). Hair cortisol data was log transformed to correct skewness. Establishing mediation requires the mediator to be associated with the exposure (path *a*) and the outcome (path *b*), so we first tested associations of (i) perceived weight discrimination with (a) stress exposure (hair cortisol concentration) using linear regression and (b) health-risk behaviours (smoking, physical inactivity, and alcohol consumption) using logistic regression; and (ii) stress exposure and health-risk behaviours with health and wellbeing outcomes using logistic or linear regression, as appropriate. Where associations were significant, we used the *sgmediation* command in STATA, which calculates total (path *c*), direct (path *c*'), and indirect (path $a \times b$) effects, and tests the significance of the indirect effect using the Sobel test. We used bootstrapping with 5,000 sampling replications to estimate the 95% confidence interval and calculated effect ratios reflecting the proportion of the total effect of the independent variable on the dependent variable that is explained by the mediator. Mediation models were adjusted for age, sex, ethnicity, wealth, BMI, and status/score on the outcome variable at baseline.

For peer review only - http://bmjopen.bmj.com/site/about/guidelines.xhtml

We conducted three sensitivity analyses. In the first we expanded our analytic sample to include participants who provided data on perceived weight discrimination but did not have a BMI ≥25m²/kg. We assessed whether the pattern of associations between perceived weight discrimination and health and wellbeing outcomes was similar when including these participants. For our second sensitivity analysis we restricted our analytic sample to participants with BMI ≥30m²/kg and assessed whether the associations between perceived weight discrimination and health and wellbeing outcomes remained for those participants in the higher BMI ranges. For the final sensitivity analysis, we changed the frequency weight discrimination was encountered from *'in the past year vs less than once a year/never'* (main analysis) to whether the participant *ever* experienced discrimination (*'never' vs 'almost every day; at least once a week; a few times a month; a few times a year; less than once a year'*). We assessed whether our results changed when using this different definition of weight discrimination. Analyses were performed on complete cases using SPSS v.25, with the exception of the mediation models which were run in STATA v.13.

Results

In Wave 5, 9,090 participants (77.5% of those eligible) took part in the face-to-face interview, of whom 8,107 (93%) returned the self-completion questionnaire. We excluded 2,142 participants with missing data on weight discrimination or covariates (including BMI) and 1,624 with BMI <25kg/m². Our final sample for cross-sectional analysis comprised of 4,341 people, aged 52-89 years with a BMI 25.0-59.4kg/m².

Perceived weight discrimination was reported by 210 (4.8%) respondents, who were on average younger, heavier, more socioeconomically disadvantaged and more likely to be female than those who did not report weight discrimination (Table 1). Of the participants who perceived weight discrimination, 44 (21%) also reported sex discrimination, 40 (19%) reported disability discrimination, 11 (5.2%) reported race discrimination and 6 (2.9%) reported sexuality discrimination.

For peer review only - http://bmjopen.bmj.com/site/about/guidelines.xhtml

**Table 1** Sample characteristics at baseline (Wave 5)				
	**Whole sample (*n*=4341)**	**Perceived weight discrimination (*n*=210)**	**No perceived weight discrimination (*n*=4131)**	***p***
Age (years), mean (SD)	66.62 (7.96)	62.04 (6.58)	66.85 (7.95)	<0.001
Sex, % (*n*)				
Men	47.2 (2051)	34.8 (73)	47.9 (1978)	<0.001
Women	52.8 (2290)	65.2 (137)	52.1 (2153)	-
Ethnicity, % (*n*)				
White	97.9 (4250)	97.6 (205)	97.9 (4045)	0.768
Other	2.1 (91)	2.4 (5)	2.1 (86)	-
Wealth quintile, % (*n*)				
1 (poorest)	15.5 (672)	26.7 (56)	14.9 (616)	<0.001
2	20.6 (896)	29.0 (61)	20.2 (835)	-
3	21.0 (913)	18.6 (39)	21.2 (874)	-
4	21.8 (946)	16.2 (34)	22.1 (912)	-
5 (richest)	21.1 (914)	9.5 (20)	21.6 (894)	-
BMI (kg/m^2), mean (SD)	30.37 (4.47)	36.63 (6.17)	30.05 (4.12)	<0.001
BMI, body mass index.				

Cross-sectionally, perceived weight discrimination was associated with significantly higher odds of fair/poor self-rated health, limiting long-standing illness, and depressive symptoms, and lower mean ratings of quality of life and life satisfaction, after adjustment for age, sex, ethnicity, wealth, and BMI (Table 2). Prospectively, perceived weight discrimination was associated with significantly higher odds of future fair/poor self-rated health and depressive symptoms, but differences in limiting long-standing illness, quality of life, and life satisfaction were not statistically significant after adjustment for covariates including baseline status/score on the outcome variable (Table 2). Analysing depression and self-rated health as continuous variables did not change the pattern of results (Table 2).

For peer review only

Table 2 Cross-sectional and prospective associations of perceived weight discrimination with health and wellbeing outcomes (path *c* on Figure 1)

	Cross-sectional (Wave 5)		Prospective (Wave 7)	
	**No perceived weight discrimination**	**Perceived weight discrimination**	**No perceived weight discrimination**	**Perceived weight discrimination**
Fair/poor self-rated health				
n included in analysis	4130	208	3705	188
% (*n*) reporting outcome	22.5 (928)	48.1 (100)	26.3 (973)	53.7 (101)
Adjusted OR [95% CI]<br>*p*	1.00 (ref)	2.05 [1.49; 2.82]<br><0.001	1.00 (ref)	1.63 [1.10; 2.40]<br>0.014
Self-rated health (range 1-5)				
n included in analysis	4130	208	3705	188
Mean (SD) score	2.73 (1.04)	3.47 (1.06)	2.85 (1.05)	3.56 (0.99)
Adjusted *B* [95% CI]<br>*p*	Ref	0.44 [0.29; 0.58]<br><0.001	Ref	0.14 [0.02; 0.27]<br>0.026
Limiting long-standing illness				
n included in analysis	4129	209	3740	190
% (*n*) reporting outcome	32.2 (1330)	55.0 (115)	36.2 (1354)	56.8 (108)
Adjusted OR [95% CI]<br>*p*	1.00 (ref)	1.76 [1.29; 2.41]<br><0.001	1.00 (ref)	1.24 [0.85; 1.79]<br>0.268
Depressive symptoms				
n included in analysis	4098	210	3643	188
% (*n*) reporting outcome	11.9 (487)	28.6 (60)	10.8 (392)	29.8 (56)
Adjusted OR [95% CI]<br>*p*	1.00 (ref)	2.01 [1.41; 2.85]<br><0.001	1.00 (ref)	2.37 [1.57; 3.60]<br><0.001
Depressive symptoms (range 0-8)				
n included in analysis	4098	210	3643	188
Mean (SD) score	1.30 (1.78)	2.60 (2.42)	1.24 (1.73)	2.31 (2.12)
Adjusted *B* [95% CI]<br>*P*	Ref	0.94 [0.68; 1.20]<br><0.001	Ref	0.38 [0.15; 0.62]<br><0.001
Quality of life (range 0-57)				
n included in analysis	3942	203	3106	154
Mean (SD) score	41.72 (8.36)	34.24 (9.57)	42.19 (8.47)	36.56 (9.34)
Adjusted *B* [95% CI]<br>*p*	Ref	-5.82 [-7.01; -4.62]<br><0.001	Ref	0.01 [-0.96; 0.97]<br>0.992
Life satisfaction (range 0-30)				
n included in analysis	4008	199	3240	160
Mean (SD) score	21.05 (6.02)	17.45 (7.62)	21.22 (6.03)	18.46 (7.31)
Adjusted *B* [95% CI]<br>*p*	Ref	-2.36 [-3.25; -1.47]<br><0.001	Ref	-0.15 [-0.89; 0.59]<br>0.691

OR, odds ratio. CI, confidence interval. SD, standard deviation.
ORs, *B*s and 95% CIs are adjusted for age, sex, ethnicity, wealth, and body mass index. Prospective results are additionally adjusted for status/score on the outcome variable at baseline (Wave 5).
Ref = reference category.

For peer review only

For peer review only - http://bmjopen.bmj.com/site/about/guidelines.xhtml

Table 3 Associations of perceived weight discrimination at Wave 5 with health behaviours and stress exposure at Wave 6 (path *a* on Figure 1)

	No perceived weight discrimination	Perceived weight discrimination
Smoking status		
n included in analysis	4131	210
% (*n*) current smoker	8.4 (347)	14.3 (30)
Adjusted OR [95% CI]<br>*p*	1.00 (ref)	1.43 [0.91-2.25]<br>0.120
Physical inactivity		
n included in analysis	4131	210
% (*n*) physically inactive	5.9 (242)	13.8 (29)
Adjusted OR [95% CI]<br>*p*	1.00 (ref)	1.90 [1.18; 3.05]<br>0.008
Alcohol consumption		
n included in analysis	3890	186
% (*n*) frequent alcohol consumption	20.6 (801)	12.9 (24)
Adjusted OR [95% CI]<br>*p*	1.00 (ref)	0.93 [0.59; 1.49]<br>0.774
Hair cortisol concentration		
n included in analysis	2456	128
Mean (SD) log pg/mg	0.90 (0.57)	1.09 (0.65)
Adjusted *B* [95% CI]<br>*p*	Ref	0.14 [0.03; 0.25]<br>0.012

OR, odds ratio. CI, confidence interval.
ORs and 95% CIs are adjusted for age, sex, ethnicity, wealth, and body mass index.

For peer review only

We assessed whether prospective associations observed between perceived weight discrimination and self-rated health and depressive symptoms were mediated by stress exposure and health-risk behaviours (Figure 1; as limiting long-standing illness, quality of life, and life satisfaction were not prospectively associated with discrimination these were not investigated). After adjustment for covariates, participants who reported weight discrimination in Wave 5 had significantly higher hair cortisol concentrations (indicative of stress exposure) and higher odds of physically inactivity in Wave 6 than those who did not (Table 3; path *a* Figure 1). No association with smoking or alcohol consumption was observed (Table 3). Physical inactivity in Wave 6 was significantly associated with higher odds of fair/poor self-rated health and depressive symptoms in Wave 7 (Table 4; path *b* Figure 1). While hair cortisol concentrations in Wave 6 were not significantly associated with depressive symptoms in Wave 7 (Table 4; path *b* Figure 1), each one-unit increase in hair cortisol concentration (log pg/mg) was associated with 17% higher odds of subsequently reporting fair/poor self-rated

For peer review only - http://bmjopen.bmj.com/site/about/guidelines.xhtml

health, which while not quite statistically significant (*p*=0.066) warranted further investigation as a potential mediator.

Table 4 Associations of level of physical activity and stress exposure at Wave 6 with health and wellbeing outcomes (prospectively associated with perceived weight discrimination) at Wave 7 (path *b* on Figure 1)

	Fair/poor self-rated health		Depressive symptoms	
	**No**	**Yes**	**No**	**Yes**
Physical inactivity				
n included in analysis	2821	1075	3404	456
% (*n*) physically inactive	3.4 (96)	12.4 (133)	5.1 (173)	11.4 (52)
Adjusted OR [95% CI]<br>*p*	1.00 (ref)	2.57 [1.92; 3.45]<br><0.001	1.00 (ref)	1.71 [1.21; 2.42]<br>0.002
Hair cortisol concentration				
n included in analysis	1699	623	2008	292
Mean (SD) log pg/mg	0.89 (0.57)	0.97 (0.58)	0.91 (0.57)	0.91 (0.57)
Adjusted OR [95% CI]<br>*p*	1.00 (ref)	1.17 [0.99; 1.38]<br>0.066	1.00 (ref)	0.93 [0.74; 1.17]<br>0.538

OR, odds ratio. CI, confidence interval.
ORs and 95% CIs are adjusted for age, sex, ethnicity, wealth, and body mass index.
Ref = reference category.

Results of mediation analyses are summarised in Table 5 (path *c*, path *c'*, and indirect effects Figure 1). There was no evidence of mediation, with effect ratios indicating that differences in physical inactivity explained approximately 4.2% of the association between perceived weight discrimination and self-rated health and 1.5% of the association between perceived weight discrimination and depressive symptoms, and differences in hair cortisol concentrations explained 1.5% of the association between perceived weight discrimination and self-rated health. None of these indirect effects were statistically significant.

For peer review only

Table 5 Models testing mediation of the associations between perceived weight discrimination and fair/poor self-rated health and depressive symptoms by level of physical activity and stress exposure (see Figure 1)

	Mediation by physical inactivity				
	**Coeff.**	**SE**	***p****	**Bootstrap 95% CI**	**Effect ratio**
**Fair/poor self-rated health**					
Total effect (path *c*)	0.081	0.030	0.007	-	-
Direct effect (path *c'*)	0.077	0.030	0.009	-	-
Indirect effect (via mediator)	0.003	0.002	0.062	-0.0002; 0.009	0.042
**Depressive symptoms**					
Total effect (path *c*)	0.113	0.023	<0.001	-	-
Direct effect (path *c'*)	0.111	0.023	<0.001	-	-
Indirect effect (via mediator)	0.002	0.001	0.169	-0.001; 0.005	0.015
	**Mediation by hair cortisol concentration**				
**Fair/poor self-rated health**					
Total effect (path *c*)	0.080	0.038	0.034	-	-
Direct effect (path *c'*)	0.079	0.038	0.037	-	-
Indirect effect (via mediator)	0.001	0.878	0.380	-0.001; 0.005	0.015

Analyses are adjusted for age, sex, ethnicity, wealth, body mass index, and status on the outcome variable at baseline (Wave 5).
Coeff. = coefficient; SE = standard error; CI = confidence interval; BMI = body mass index.
**p* values shown for indirect effects are derived from the Sobel test for consistency with total and direct effects, however bootstrap 95% confidence intervals provide a more robust indication of significant mediation (see Method for more details).

For peer review only

For peer review only - http://bmjopen.bmj.com/site/about/guidelines.xhtml

Sensitivity analyses

In the first sensitivity analysis, we expanded our sample to include participants who provided data on perceived weight discrimination but did not have a BMI in the overweight/obesity range (see Supplementary Table 1 for sample characteristics). We assessed whether the pattern of associations between weight discrimination and health and wellbeing outcomes was similar when including these participants (Supplementary Table 2). The results remained unchanged when including participants who did not have overweight/obesity.

In the second sensitivity analysis, we restricted the sample to participants with BMI ≥30m^2/kg (see Supplementary Table 3 for the characteristics of this restricted sample). We assessed whether the observed relationships between perceived weight discrimination and health and wellbeing outcomes were similar in these participants in the higher BMI ranges. As can be seen the results were similar when restricting the sample to those with ≥30m^2/kg (see Supplementary Table 4).

For our final sensitivity analysis, we changed the frequency weight discrimination was encountered from *at least once a year* (main analysis) to whether the participant *ever* experienced discrimination (*'never' vs all other options*). Ever encountering weight discrimination had a similar impact on cross-sectional and prospective health and wellbeing outcomes (Supplementary Table 5).

Discussion

In this large cohort of older adults, cross-sectionally, perceived weight discrimination was associated with higher odds of fair/poor self-rated health, limiting long-standing illness, and depressive symptoms, and lower quality of life and life satisfaction. Prospectively, perceived weight discrimination was associated with higher odds of fair/poor self-rated health and depressive symptoms after adjustment for baseline status. All associations were independent of age, sex, ethnicity, wealth, and BMI. However, while those who reported weight discrimination had higher odds of physically inactivity and higher hair cortisol concentrations than those who did not, there was no evidence that these variables mediated associations between perceived weight discrimination and poor health and wellbeing.

For peer review only - http://bmjopen.bmj.com/site/about/guidelines.xhtml

Our finding that perceived weight discrimination is associated with poorer health and wellbeing is consistent with previous studies (7–11). While our results provide prospective evidence linking perceived weight discrimination with self-rated health and depressive symptoms, as has been observed in other samples (7,8,11), we failed to replicate a prospective association with life satisfaction (7) or detect any prospective association with limiting long-standing illness or quality of life. It is unclear whether this is the result of differences in measurement (e.g., assessing limiting long-standing illness vs total disease burden), sample (e.g., age range, the lack of ethnic diversity in the current sample), setting (i.e., England versus the US), or covariates used (highlighting the possibility of uncontrolled confounding). Nonetheless, taking our findings in the context of the literature, it is evident that weight stigma is a social determinant of health and a driver of health inequalities (45). As such, it is not only a social justice issue, but also a public health issue. Further, we observed an inverse association between perceived weight discrimination and wealth, suggesting that those of lower socioeconomic position could be more vulnerable to the deleterious effects of weight discrimination. This inverse association is in line with previous reports in ELSA, but not other studies (46). Therefore, the role of socioeconomic position in the associations between weight discrimination, health and wellbeing, warrants further attention.

A key study aim was to explore the role of stress and health-risk behaviours as pathways underpinning associations between weight discrimination and health and wellbeing (47). In line with previous findings, perceived weight discrimination was associated with increased stress (higher hair cortisol concentrations) and physical inactivity (19,20), but these variables explained only a very small and non-significant proportion of the total associations of perceived weight discrimination with self-rated health and depressive symptoms. Associations with smoking and alcohol consumption were not statistically significant, so these variables were not considered plausible mediators. There are several potential explanations for these findings. The variables we assessed make only small contributions to the impact of weight discrimination on health and wellbeing, and there are other variables more important to this relationship that we were unable to account for in our modelling. We had no information on dietary factors or eating behaviours such as unsupervised and/or unsafe dieting practices, binge eating, disordered eating, and weight cycling. Therefore, we were unable to explore these as potential mediators. Given evidence that people who experience weight discrimination are at increased risk of overeating, disordered eating (48,49) and have a greater risk of binge eating disorder (25) than those who do not report discrimination these present important unexamined pathways from weight discrimination to poor health. It is plausible that the use of maladaptive dietary and eating behaviours as a means of coping with weight discrimination could lead to a vicious cycle of further distress, weight gain and

For peer review only - http://bmjopen.bmj.com/site/about/guidelines.xhtml

disproportionate burden of weight discrimination (14,25). This represents an important avenue of further research, particularly considering the lack of mediation by other factors in the current study. Future prospective studies should explicitly set out to measure the role of diet, dieting behaviour, weight cycling, and disordered eating (such as binge eating disorder) to understand how these factors are involved in the relationship between weight discrimination and health. This is of relevance in both community settings (as in this study) and in healthcare settings. There is evidence that healthcare providers can hold negative attitudes and believe stereotypes about people with overweight/obesity (50,51). This can have negative implications for both the quality of care received and can result in people avoiding healthcare settings for fear of encountering weight discrimination (51). This of relevance to the current study as we observed a cross-sectional association between perceived weight discrimination and reports of limiting longstanding illness. Indeed, people with obesity-related health conditions (such as diabetes or heart disease) have more frequent encounters with healthcare professionals than those without these conditions. The exploration of healthcare settings as a location where weight discrimination could be encountered represents an important area for future work.

Unmeasured variables including negative emotions such as psychological distress and feeling of anxiety which could be associated with weight discrimination. It is possible that these emotions could mediate the link between perceived weight discrimination and health, or these could be viewed as negative outcomes in their own right. Social isolation and loneliness play a role in weight-related conditions(52,53). Changes in social contact or relationships could occur as a result of perceiving weight discrimination. The relationship between weight discrimination, indicators of social connectedness and health remain to be teased out. We included self-rated health as an outcome in this study, as this is considered to better and more broadly capture 'health' than most other indicators (54). However, the association between perceived weight discrimination and specific health conditions (including weight-related conditions) represents an important area for future work. Another explanation for the non-significant mediation observed in the current study is that the variables we analysed as potential mediators were not adequately captured by the available measures. For example, hair cortisol was examined in 2cm long hair samples, and thus reflected approximately two months' exposure to circulating cortisol. While there are advantages in using hair cortisol over other more transient cortisol measures (e.g., in blood or saliva) two months is still a relatively short interval given that these data were collected on average two years after discrimination was assessed. In addition, research using hair cortisol is in its infancy and it is unclear whether 1 standard deviation increase in hair cortisol is clinically meaningful (55). Further, cortisol does not just reflect stress-related activation of the HPA axis. Cortisol parameters are

For peer review only - http://bmjopen.bmj.com/site/about/guidelines.xhtml

altered in weight-related conditions such as sub-clinical hypercortisolism and diabetes (56,57). Cortisol dynamics are complex, with heightened as well as blunted responses associated with weight-related ill-health (58,59). These complexities warrant attention in future work. It is also possible that other stress-related biological pathways (such as chronic inflammation) could play a role in the weight discrimination-health relationship. Physical activity was self-reported in response to three basic questions on the frequency of light, moderate or vigorous exercise. Comparisons of self-reported versus objective measures of physical activity have shown that there are often substantial differences between what people say and what they actually do (60). Our measures of smoking behaviour and alcohol consumption were crude, as both factors were self-reported using a single item measure. Further research should aim to replicate our analyses with different and more detailed measures of the constructs we have examined (stress, physical inactivity, smoking, and alcohol consumption) to establish whether the null results of our mediation analyses are the result of limitations in our measures (such as self-reported health risk behaviours) or an accurate representation of the causal pathways linking perceived weight discrimination with poorer health and wellbeing outcomes.

Strengths of the study include the large sample, prospective study design, and assessment of multiple mediators and outcomes. However, there were several limitations. Weight was not measured in the same data collection wave as discrimination, and participants may have changed weight status since experiencing discrimination. The prevalence of weight discrimination in the sample was low which limits the generalisability of our findings. Further not all participants who perceived weight discrimination provided hair cortisol samples meaning we may not have been able to detect small effects. We did not have complete data on weight, and it is possible that people most troubled by their weight were more likely to decline to be weighed. It is also possible that individuals most vulnerable to negative effects of weight discrimination opted not to complete the item on weight discrimination. The discrimination questions asked about five broad discriminatory situations and was not tailored for weight discrimination. This may have helped avoid bias or priming, as participants were able to attribute multiple reasons for their experience of discrimination(30–33,46). However, other measures with more specific items on experiences weight discrimination, for example having to pay more on public transport for occupying two passenger seats or being viewed unfavourably as a potential romantic partner, may have garnered different results. Our measure was not validated to assess weight discrimination. The use of tools such as the Stigmatizing Situations Inventory (61) or the Fat Phobia Scale (62) that including specific items on weight discrimination, weight bias internalization and experience of weight discrimination by close family, romantic partners or healthcare professionals may also have produced different findings. Further, weight discrimination was only measured on one occasion meaning we

For peer review only - http://bmjopen.bmj.com/site/about/guidelines.xhtml

had insufficient information of the dose or duration or this form of discrimination. This increases the risk of misclassification bias.

Finally, while the data were drawn from a representative sample, there was missing data and loss to follow-up, meaning the results may not be representative of the target population. In addition, as the sample comprised predominantly white middle-aged and older adults. The results may not generalise to other samples, in particular, younger samples who may be more likely to report weight discrimination (63). We did not have data on other negative emotional states such as psychological distress and anxiety which are likely relevant to the links between weight discrimination, health, and wellbeing.

Overall, these results provide further prospective evidence linking weight-based discrimination with poorer health and wellbeing. However, while perceived weight discrimination was associated with greater stress exposure and physical inactivity, these variables explained only a small part of the association between weight discrimination and poorer health and wellbeing. This leaves unanswered questions about the pathways through which weight discrimination adversely impacts health and wellbeing. Further research is required using large prospective-based studies that have been specifically designed to assess these questions and to test unmeasured factors (e.g., diet and eating behaviour). There is a great need to include more diverse samples in this future work to improve the generalisability of study findings and also to understand intersectional issues (e.g., how weight discrimination may interact with other forms of discrimination such as racism to influence health). Understanding of mechanisms of action is required if policies and interventions to mitigate the health burden of weight discrimination are to be developed. This is imperative as the literature and the findings of the current study underscore the need to tackle weight discrimination to improve population health.

For peer review only

For peer review only - http://bmjopen.bmj.com/site/about/guidelines.xhtml

Declarations

Contributor statement: RAH conducted statistical analyses, wrote, edited and reviewed the manuscript. SEJ conducted statistical analyses, wrote, edited and reviewed the manuscript. EC edited and reviewed the manuscript. AS, edited and reviewed the manuscript.

Competing interests: None

Funding: This research was supported by the Economic and Social Research Council (https://esrc.ukri.org/), grant number ES/R005990/1(awarded to AS and SEJ), and the Academy of Medical Sciences/the Wellcome Trust/the Government Department of Business, Energy and Industrial Strategy/the British Heart Foundation/Diabetes UK Springboard Award (SBF006\1036) (awarded to RH). The funders had no role in study design, data collection and analysis, decision to publish, or preparation of the manuscript.

Data sharing: Data from the English Longitudinal Study of Ageing are available to download from g2aging.org.

Ethics approval statement: Ethical approval for ELSA was obtained from the London Multicentre Research and Ethics Committee (MREC/01/02/91). All participants provided informed consent.

For peer review only

References

1. Adults' average BMI [Internet]. Health Survey for England. 2019 [cited 2020 Mar 19]. Available from: http://healthsurvey.hscic.gov.uk/data-visualisation/data-visualisation/explore-the-trends/weight/adult/bmi.aspx

2. NHS Digital. Statistics on Obesity, Physical Activity and Diet, England, 2019 [Internet]. 2019 [cited 2020 Mar 19]. Available from: https://digital.nhs.uk/data-and-information/publications/statistical/statistics-on-obesity-physical-activity-and-diet/statistics-on-obesity-physical-activity-and-diet-england-2019/part-3-adult-obesity

3. Wardle J, Boniface D. Changes in the distributions of body mass index and waist circumference in English adults, 1993/1994 to 2002/2003. Int J Obes. 2008;32(3):527–32.

4. Puhl RM, Heuer CA. The Stigma of Obesity: A Review and Update. Obesity. 2009 May 1;17(5):941–64.

5. Emmer C, Bosnjak M, Mata J. The association between weight stigma and mental health: A meta-analysis. Obes Rev. 2020;21(1):e12935.

6. Puhl R, Suh Y. Health Consequences of Weight Stigma: Implications for Obesity Prevention and Treatment. Curr Obes Rep. 2015 Apr 1;4(2):182–90.

7. Sutin AR, Stephan Y, Carretta H, Terracciano A. Perceived discrimination and physical, cognitive, and emotional health in older adulthood. Am J Geriatr Psychiatry Off J Am Assoc Geriatr Psychiatry. 2015 Feb;23(2):171–9.

8. Hatzenbuehler ML, Keyes KM, Hasin DS. Associations between perceived weight discrimination and the prevalence of psychiatric disorders in the general population. Obes Silver Spring Md. 2009 Nov;17(11):2033–9.

9. Hackman J, Maupin J, Brewis AA. Weight-related stigma is a significant psychosocial stressor in developing countries: Evidence from Guatemala. Soc Sci Med 1982. 2016;161:55–60.

10. Sutin AR, Stephan Y, Grzywacz JG, Robinson E, Daly M, Terracciano A. Perceived weight discrimination, changes in health, and daily stressors. Obesity. 2016 Oct 1;24(10):2202–9.

11. Schafer MH, Ferraro KF. The stigma of obesity: does perceived weight discrimination affect identity and physical health? Soc Psychol Q. 2011;74(1):76–97.

12. Sutin AR, Terracciano A. Sources of weight discrimination and health. Stigma Health. 2017;2(1):23–7.

13. Sutin AR, Stephan Y, Terracciano A. Weight Discrimination and Risk of Mortality. Psychol Sci. 2015 Nov 1;26(11):1803–11.

14. Tomiyama AJ. Weight stigma is stressful. A review of evidence for the Cyclic Obesity/Weight-Based Stigma model. Appetite. 2014 Nov;82:8–15.

For peer review only - http://bmjopen.bmj.com/site/about/guidelines.xhtml

15. Schvey NA, Puhl RM, Brownell KD. The stress of stigma: exploring the effect of weight stigma on cortisol reactivity. Psychosom Med. 2014 Feb;76(2):156–62.

16. Himmelstein MS, Incollingo Belsky AC, Tomiyama AJ. The weight of stigma: cortisol reactivity to manipulated weight stigma. Obes Silver Spring Md. 2015 Feb;23(2):368–74.

17. Puhl RM, Brownell KD. Confronting and coping with weight stigma: an investigation of overweight and obese adults. Obes Silver Spring Md. 2006 Oct;14(10):1802–15.

18. McEwen BS, Gianaros PJ. Central role of the brain in stress and adaptation: links to socioeconomic status, health, and disease. Ann N Y Acad Sci. 2010 Feb;1186:190–222.

19. Jackson SE, Kirschbaum C, Steptoe A. Perceived weight discrimination and chronic biochemical stress: A population-based study using cortisol in scalp hair. Obes Silver Spring Md. 2016 Dec;24(12):2515–21.

20. Tomiyama AJ, Epel ES, McClatchey TM, Poelke G, Kemeny ME, McCoy SK, et al. Associations of weight stigma with cortisol and oxidative stress independent of adiposity. Health Psychol Off J Div Health Psychol Am Psychol Assoc. 2014 Aug;33(8):862–7.

21. Born JM, Lemmens SGT, Rutters F, Nieuwenhuizen AG, Formisano E, Goebel R, et al. Acute stress and food-related reward activation in the brain during food choice during eating in the absence of hunger. Int J Obes. 2010;34(1):172–81.

22. Groesz LM, McCoy S, Carl J, Saslow L, Stewart J, Adler N, et al. What is eating you? Stress and the drive to eat. Appetite. 2012 Apr;58(2):717–21.

23. Kandiah J, Yake M, Jones J, Meyer M. Stress influences appetite and comfort food preferences in college women. Nutr Res. 2006;26(3):118–23.

24. Wu YK, Berry DC. Impact of weight stigma on physiological and psychological health outcomes for overweight and obese adults: A systematic review. J Adv Nurs. 2018;74(5):1030–42.

25. Salvia MG, Ritholz MD, Craigen KLE, Quatromoni PA. Women's perceptions of weight stigma and experiences of weight-neutral treatment for binge eating disorder: a qualitative study. eClinicalMedicine. 2023 Feb 1;56:101811.

26. Vucetic Z, Reyes TM. Central dopaminergic circuitry controlling food intake and reward: implications for the regulation of obesity. Wiley Interdiscip Rev Syst Biol Med. 2010 Sep 1;2(5):577–93.

27. Bethancourt HJ, Rosenberg DE, Beatty T, Arterburn DE. Barriers to and Facilitators of Physical Activity Program Use Among Older Adults. Clin Med Res. 2014 Jan 10;cmr.2013.1171.

28. Steptoe A, Breeze E, Banks J, Nazroo J. Cohort profile: the English Longitudinal Study of Ageing. Int J Epidemiol. 2013 Dec;42(6):1640–8.

29. Smith J, Ryan L, Sonnega A, Weir DR. Psychosocial and lifestyle questionnaire 2006–2016: Documentation report core section. Ann Arbor, MI, USA.: The HRS Psychosocial Working Group:; 2017.

For peer review only - http://bmjopen.bmj.com/site/about/guidelines.xhtml

30. Jackson SE, Hackett RA, Steptoe A. Associations between age discrimination and health and wellbeing: cross-sectional and prospective analysis of the English Longitudinal Study of Ageing. Lancet Public Health. 2019 Apr 1;4(4):e200–8.

31. Jackson SE, Hackett RA, Pardhan S, Smith L, Steptoe A. Association of Perceived Discrimination With Emotional Well-being in Older Adults With Visual Impairment. JAMA Ophthalmol. 2019 01;137(7):825–32.

32. Scott W, Jackson SE, Hackett RA. Perceived discrimination, health, and well-being among adults with and without pain: a prospective study. PAIN. 2022;163(2):258–66.

33. Jackson SE, Hackett RA, Grabovac I, Smith L, Steptoe A. Perceived discrimination, health and wellbeing among middle-aged and older lesbian, gay and bisexual people: A prospective study. PLOS ONE. 2019 May 10;14(5):e0216497.

34. Jackson SE, Beeken RJ, Wardle J. Obesity, perceived weight discrimination, and psychological well-being in older adults in England. Obesity. 2015 May 1;23(5):1105–11.

35. Jackson SE, Steptoe A, Beeken RJ, Croker H, Wardle J. Perceived weight discrimination in England: a population-based study of adults aged ⩾50 years. Int J Obes. 2015 May;39(5):858–64.

36. Jackson SE, Beeken RJ, Wardle J. Perceived weight discrimination and changes in weight, waist circumference, and weight status. Obesity. 2014 Dec 1;22(12):2485–8.

37. Jackson SE, Steptoe A. Association between perceived weight discrimination and physical activity: a population-based study among English middle-aged and older adults. BMJ Open. 2017 Mar 1;7(3):e014592.

38. Jackson SE, Steptoe A. Obesity, perceived weight discrimination, and hair cortisol: a population-based study. Psychoneuroendocrinology. 2018 Dec 1;98:67–73.

39. Steptoe A, Jackson SE. The Life Skills of Older Americans: Association with Economic, Psychological, Social, and Health Outcomes. Sci Rep. 2018 Jul 5;8(1):9669.

40. Radloff LS. The CES-D scale. Appl Psychol Meas. 1977;1(3):385–401.

41. Steffick DE. Documentation of affective functioning measures in the Health and Retirement Study. HRS Documentation Report DR-005; 2000.

42. Hyde M, Wiggins RD, Higgs P, Blane DB. A measure of quality of life in early old age: the theory, development and properties of a needs satisfaction model (CASP-19). Aging Ment Health. 2003;7(3):186–94.

43. Diener E, Emmons RA, Larsen RJ, Griffin S. The satisfaction with life scale. J Assess. 1985;49(1):71–5.

44. Jackson SE, Kirschbaum C, Steptoe A. Hair cortisol and adiposity in a population-based sample of 2,527 men and women aged 54 to 87 years. Obesity. 2017;25(3):539–44.

For peer review only - http://bmjopen.bmj.com/site/about/guidelines.xhtml

45. Hatzenbuehler ML, Phelan JC, Link BG. Stigma as a Fundamental Cause of Population Health Inequalities. Am J Public Health. 2013 May;103(5):813–21.

46. Amirova A, Rimes KA, Hackett RA. Perceived discrimination in middle-aged and older adults: Comparison between England and the United States. Front Public Health [Internet]. 2022 [cited 2023 Feb 21];10. Available from: https://www.frontiersin.org/articles/10.3389/fpubh.2022.975776

47. Pascoe EA, Smart Richman L. Perceived discrimination and health: A meta-analytic review. Psychol Bull. 2009;135(4):531–54.

48. Puhl RM, Moss-Racusin CA, Schwartz MB. Internalization of weight bias: Implications for binge eating and emotional well-being. Obes Silver Spring Md. 2007 Jan;15(1):19–23.

49. Sutin A, Robinson E, Daly M, Terracciano A. Weight discrimination and unhealthy eating-related behaviors. Appetite. 2016 01;102:83–9.

50. Sabin JA, Marini M, Nosek BA. Implicit and Explicit Anti-Fat Bias among a Large Sample of Medical Doctors by BMI, Race/Ethnicity and Gender. PLOS ONE. 2012 Nov 7;7(11):e48448.

51. Phelan SM, Burgess DJ, Yeazel MW, Hellerstedt WL, Griffin JM, van Ryn M. Impact of weight bias and stigma on quality of care and outcomes for patients with obesity. Obes Rev. 2015;16(4):319–26.

52. Hackett RA, Hudson JL, Chilcot J. Loneliness and type 2 diabetes incidence: findings from the English Longitudinal Study of Ageing. Diabetologia. 2020 Nov 1;63(11):2329–38.

53. Valtorta NK, Kanaan M, Gilbody S, Ronzi S, Hanratty B. Loneliness and social isolation as risk factors for coronary heart disease and stroke: systematic review and meta-analysis of longitudinal observational studies. Heart. 2016 Jul 1;102(13):1009–16.

54. Jylhä M. What is self-rated health and why does it predict mortality? Towards a unified conceptual model. Soc Sci Med 1982. 2009 Aug;69(3):307–16.

55. Iob E, Steptoe A. Cardiovascular Disease and Hair Cortisol: a Novel Biomarker of Chronic Stress. Curr Cardiol Rep. 2019 Aug 30;21(10):116.

56. Chiodini I. Diagnosis and Treatment of Subclinical Hypercortisolism. J Clin Endocrinol Metab. 2011 May 1;96(5):1223–36.

57. Hackett RA, Steptoe A, Kumari M. Association of Diurnal Patterns in Salivary Cortisol With Type 2 Diabetes in the Whitehall II Study. J Clin Endocrinol Metab. 2014 Dec 1;99(12):4625–31.

58. McEwen BS. Stress, adaptation, and disease. Allostasis and allostatic load. Ann N Y Acad Sci. 1998 May 1;840:33–44.

59. Steptoe A, Hackett RA, Lazzarino AI, Bostock S, Marca RL, Carvalho LA, et al. Disruption of multisystem responses to stress in type 2 diabetes: Investigating the dynamics of allostatic load. Proc Natl Acad Sci. 2014 Nov 4;111(44):15693–8.

For peer review only

For peer review only - http://bmjopen.bmj.com/site/about/guidelines.xhtml

60. Kapteyn A, Banks J, Hamer M, Smith JP, Steptoe A, Soest A van, et al. What they say and what they do: comparing physical activity across the USA, England and the Netherlands. J Epidemiol Community Health. 2018 Jun 1;72(6):471–6.

61. Myers A, Rosen JC. Obesity stigmatization and coping: relation to mental health symptoms, body image, and self-esteem. Int J Obes Relat Metab Disord J Int Assoc Study Obes. 1999 Mar;23(3):221–30.

62. Bacon JG, Scheltema KE, Robinson BE. Fat phobia scale revisited: the short form. Int J Obes Relat Metab Disord J Int Assoc Study Obes. 2001 Feb;25(2):252–7.

63. Puhl RM, Andreyeva T, Brownell KD. Perceptions of weight discrimination: prevalence and comparison to race and gender discrimination in America. Int J Obes. 2008 Jun;32(6):992–1000.

For peer review only

For peer review only - http://bmjopen.bmj.com/site/about/guidelines.xhtml

Figures legends

Figure 1. Mediation model of associations between perceived weight discrimination and health and wellbeing outcomes via health behaviours and stress exposure

For peer review only

For peer review only - http://bmjopen.bmj.com/site/about/guidelines.xhtml

Figures

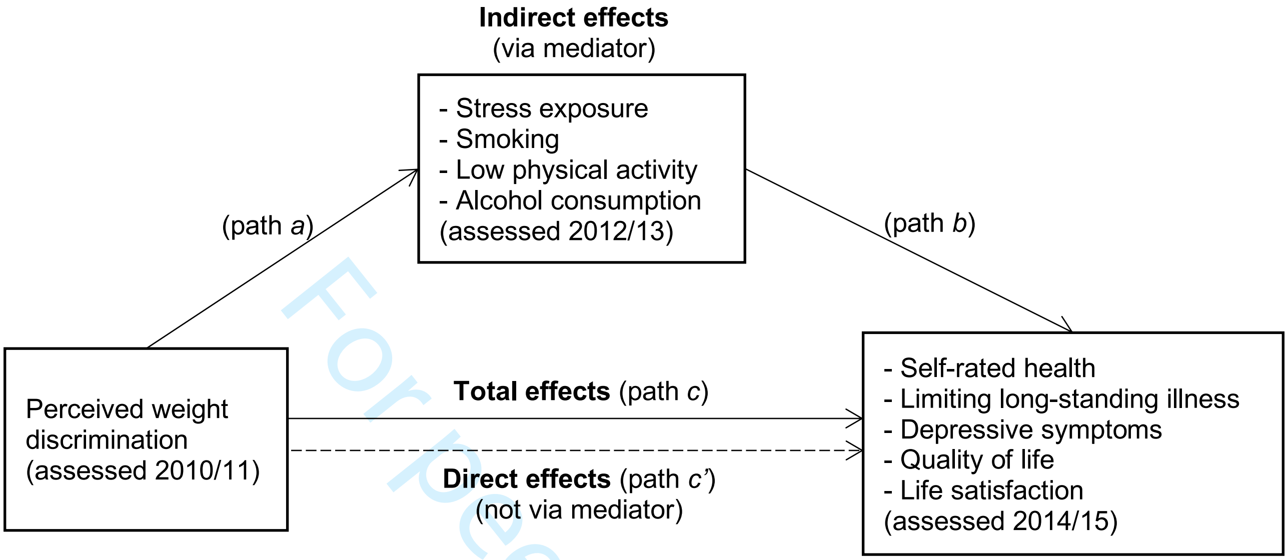

Figure 1. Mediation model of associations between perceived weight discrimination and health and wellbeing outcomes via health behaviours and stress exposure

For peer review only

Sensitivity analyses - Tables

**Supplementary Table 1** Sample characteristics at baseline (Wave 5) **all weight categories**					
		**Whole sample (*n*=5965)**	**Perceived weight discrimination (*n*=219)**	**No perceived weight discrimination (*n*=5746)**	***p***
Age (years), mean (SD)		66.56 (8.13)	62.16 (6.74)	66.72 (8.13)	<0.001
Sex, % (*n*)					
	Men	44.5 (2655)	35.2 (77)	44.9 (2578)	=0.005
	Women	55.5 (3310)	64.8 (142)	55.1 (3168)	-
Ethnicity, % (*n*)					
	White	97.9 (5841)	97.7 (214)	97.9 (5627)	0.829
	Other	2.1 (124)	2.3 (5)	2.1 (199)	-
Wealth quintile, % (*n*)					
	1 (poorest)	14.6 (868)	26.0 (57)	14.1 (811)	<0.001
	2	19.8 (1183)	30.6 (67)	19.4 (1116)	-
	3	19.9 (1190)	18.3 (40)	20.0 (1150)	-
	4	21.9 (1306)	15.5 (34)	22.1 (1272)	-
	5 (richest)	23.8 (1418)	9.6 (21)	24.3 (1397)	-
BMI (kg/m^2), mean (SD)		28.27 (5.22)	36.05 (6.69)	27.98 (4.92)	<0.001
BMI, body mass index.					

Supplementary Table 2 Cross-sectional and prospective associations of perceived weight discrimination with health and wellbeing outcomes across **all weight categories**

	Cross-sectional (Wave 5)		Prospective (Wave 7)	
	**No perceived weight discrimination**	**Perceived weight discrimination**	**No perceived weight discrimination**	**Perceived weight discrimination**
Fair/poor self-rated health				
n included in analysis	5754	217	5122	197
% (*n*) reporting outcome	20.8 (1197)	48.4 (105)	24.4 (1251)	52.8 (104)
Adjusted OR [95% CI]<br>*p*	1.00 (ref)	2.32 [1.71; 3.16]<br><0.001	1.00 (ref)	1.66 [1.15; 2.42]<br>0.008
Limiting long-standing illness				
n included in analysis	5743	218	5172	199
% (*n*) reporting outcome	30.3 (1741)	55.0 (120)	33.9 (1755)	56.3 (112)
Adjusted OR [95% CI]<br>*p*	1.00 (ref)	1.91 [1.42; 2.58]<br><0.001	1.00 (ref)	1.32 [0.92; 1.88]<br>0.130
Depressive symptoms				
n included in analysis	5695	219	5027	197
% (*n*) reporting outcome	11.7 (668)	27.9 (61)	10.4 (525)	30.5 (60)
Adjusted OR [95% CI]<br>*p*	1.00 (ref)	2.05 [1.46; 2.89]<br><0.001	1.00 (ref)	2.74 [1.84; 4.09]<br><0.001
Quality of life (range 0-57)				
n included in analysis	5497	212	4318	162
Mean (SD) score	41.96 (8.34)	34.26 (9.55)	42.54 (8.38)	36.60 (9.40)
Adjusted *B* [95% CI]<br>*p*	Ref	-6.03 [-7.18; -4.88]<br><0.001	Ref	-0.21 [-1.12; 0.70]<br>0.651
Life satisfaction (range 0-30)				
n included in analysis	5577	207	4490	167
Mean (SD) score	21.07 (6.08)	17.49 (7.60)	21.28 (6.01)	18.32 (7.32)
Adjusted *B* [95% CI]<br>*p*	Ref	-2.50 [-3.37; -1.63]<br><0.001	Ref	-0.45 [-1.16; 0.26]<br>0.213

OR, odds ratio. CI, confidence interval. SD, standard deviation.
ORs, *B*s and 95% CIs are adjusted for age, sex, ethnicity, wealth, and body mass index. Prospective results are additionally adjusted for status/score on the outcome variable at baseline (Wave 5).
Ref = reference category.

For peer review only

**Supplementary Table 3** Sample characteristics at baseline (Wave 5) **in obese participants**					
		**Whole sample (*n*=1853)**	**Perceived weight discrimination (*n*=184)**	**No perceived weight discrimination (*n*=1669)**	***p***
Age (years), mean (SD)		65.81 (7.76)	62.03 (6.66)	66.23 (7.76)	<0.001
Sex, % (*n*)					
	Men	40.5 (751)	33.2 (61)	41.3 (690)	=0.032
	Women	59.5 (1102)	66.8 (123)	58.7 (979)	-
Ethnicity, % (*n*)					
	White	97.8 (1812)	97.3 (179)	97.8 (1633)	0.624
	Other	2.2 (41)	2.7 (5)	2.2 (36)	-
Wealth quintile, % (*n*)					
	1 (poorest)	19.9 (368)	27.7 (51)	19.0 (317)	<0.001
	2	22.9 (424)	29.3 (54)	22.2 (370)	-
	3	21.3 (395)	19.6 (36)	21.5 (359)	-
	4	19.3 (358)	15.8 (29)	19.7 (329)	-
	5 (richest)	16.6 (308)	7.6 (14)	17.6 (294)	-
BMI (kg/m^2), mean (SD)		34.31 (4.13)	37.80 (5.68)	33.92 (3.73)	<0.001
BMI, body mass index.					

For peer review only

Supplementary Table 4 Cross-sectional and prospective associations of perceived weight discrimination with health and wellbeing outcomes across **in obese participants**

		**Cross-sectional (Wave 5)**			**Prospective (Wave 7)**	
		**No perceived weight discrimination**	**Perceived weight discrimination**		**No perceived weight discrimination**	**Perceived weight discrimination**
Fair/poor self-rated health						
	n included in analysis	1669	182		1478	167
	% (*n*) reporting outcome	28.4 (474)	47.3 (86)		32.3 (478)	53.9 (90)
	Adjusted OR [95% CI]<br>*p*	1.00 (ref)	1.74 [1.23; 2.46]<br>=0.002		1.00 (ref)	1.56 [1.02; 2.39]<br>0.040
Limiting long-standing illness						
	n included in analysis	1668	183		1493	169
	% (*n*) reporting outcome	39 (651)	53.6 (98)		43.4 (648)	56.2 (95)
	Adjusted OR [95% CI]<br>*p*	1.00 (ref)	1.47 [1.05; 2.01]<br>=0.027		1.00 (ref)	1.01 [0.70; 1.56]<br>0.832
Depressive symptoms						
	n included in analysis	1654	184		1451	167
	% (*n*) reporting outcome	14.1 (234)	28.8 (53)		11.9 (172)	29.9 (50)
	Adjusted OR [95% CI]<br>*p*	1.00 (ref)	1.97 [1.34; 2.88]<br><0.001		1.00 (ref)	2.15 [1.36; 3.41]<br><0.001
Quality of life (range 0-57)						
	n included in analysis	1581	178		1225	137
	Mean (SD) score	40.85 (8.44)	34.44 (9.50)		41.22 (8.61)	36.61 (9.16)
	Adjusted *B* [95% CI]<br>*p*	Ref	-5.24 [-6.57; -3.92]<br><0.001		Ref	0.08 [-1.01; 1.17]<br>0.888
Life satisfaction (range 0-30)						
	n included in analysis	1604	173		1273	139
	Mean (SD) score	20.57 (6.28)	17.51 (7.67)		20.66 (6.23)	18.60 (7.17)
	Adjusted *B* [95% CI]<br>*p*	Ref	-2.00 [-3.03; -0.98]<br><0.001		Ref	0.09 [-0.75; 0.94]<br>0.820

OR, odds ratio. CI, confidence interval. SD, standard deviation.
ORs, *B*s and 95% CIs are adjusted for age, sex, ethnicity, wealth, and body mass index. Prospective results are additionally adjusted for status/score on the outcome variable at baseline (Wave 5).
Ref = reference category.

For peer review only

Supplementary Table 5 Cross-sectional and prospective associations of perceived weight discrimination in **any domain (ever vs never)** with health and wellbeing outcomes **in overweight and obese participants**

	Cross-sectional (Wave 5)		Prospective (Wave 7)	
	**No perceived weight discrimination**	**Perceived weight discrimination**	**No perceived weight discrimination**	**Perceived weight discrimination**
Fair/poor self-rated health				
n included in analysis	4090	248	3670	223
% (*n*) reporting outcome	22.5 (919)	44.0 (109)	26.2 (960)	51.1 (114)
Adjusted OR [95% CI]<br>*p*	1.00 (ref)	1.74 [1.29; 2.35]<br><0.001	1.00 (ref)	1.68 [1.17; 2.41]<br>0.005
Limiting long-standing illness				
n included in analysis	4089	249	3705	225
% (*n*) reporting outcome	32.0 (1309)	54.6 (136)	36.0 (1333)	57.3 (129)
Adjusted OR [95% CI]<br>*p*	1.00 (ref)	1.79 [1.34; 2.39]<br><0.001	1.00 (ref)	1.33 [0.94; 1.89]<br>0.105
Depressive symptoms				
n included in analysis	4058	250	3609	222
% (*n*) reporting outcome	11.9 (483)	25.6 (64)	10.7 (385)	28.4 (63)
Adjusted OR [95% CI]<br>*p*	1.00 (ref)	1.75 [1.24; 2.45]<br><0.001	1.00 (ref)	2.54 [1.71; 3.77]<br><0.001
Quality of life (range 0-57)				
n included in analysis	3902	243	3080	180
Mean (SD) score	41.76 (8.35)	34.85 (9.50)	42.20 (8.47)	37.17 (9.39)
Adjusted *B* [95% CI]<br>*p*	Ref	-5.53 [-6.64; -4.42]<br><0.001	Ref	0.24 [-0.67; 1.15]<br>0.608
Life satisfaction (range 0-30)				
n included in analysis	3968	239	3210	190
Mean (SD) score	21.05 (6.02)	18.05 (7.41)	21.22 (6.04)	18.89 (7.09)
Adjusted *B* [95% CI]<br>*p*	Ref	-1.87 [-2.70; -1.04]<br><0.001	Ref	-0.07 [-0.76; 0.62]<br>0.844

OR, odds ratio. CI, confidence interval. SD, standard deviation.
ORs, *B*s and 95% CIs are adjusted for age, sex, ethnicity, wealth, and body mass index. Prospective results are additionally adjusted for status/score on the outcome variable at baseline (Wave 5).
Ref = reference category.

For peer review only

Supplementary materials: Questionnaire measures

Everyday discrimination

In your day-to-day life, how often have any of the following things happened to you:

	Never (0)	Less than once a year (1)	Few times a year (2)	Few times a month (3)	At least once a week(4)	Almost everyday (5)
You are treated with less respect or courtesy						
You receive poorer service than other people in restaurants and stores						
People act as if they think you are not clever						
You are threatened or harassed						
You receive poorer service or treatment than other people from doctors or hospitals						

If you answered yes to any of the above, what do you attribute this experience to

Gender
Race
Age
Weight
Physical disability
Physical appearance
Sexual orientation
Financial status
Other reason

Self-rated health

"Would you say your health is… poor/fair/good/very good/excellent?"

Limiting long-standing illness

1) "Do you have any long-standing illness, disability, or infirmity? By long-standing I mean anything that has troubled you over a period of time or that is likely to affect you over a period of time."
2) If yes, "Does this illness or disability limit your activities in any way?"

Satisfaction With Life Scale

(Diener et al., The satisfaction with life scale. J Assess. 1985;49(1):71–5).

How much of the time during the past week…
1) In most ways my life is close to my ideal.
2) The conditions of my life are excellent.
3) I am satisfied with my life.
4) So far, I have gotten the important things I want in life.
5) If I could live my life over, I would change almost nothing.
Answers range from 0 (Strongly Disagree) to 6 (Strongly agree)

For peer review only

Center for Epidemiologic Studies Depression (CESD) scale
(Radloff LS. The CES-D scale. Appl Psychol Meas. 1977;1(3):385–401).

In the past month	Yes	No
1) ... did you feel depressed?		
2) ... did you feel everything you did was an effort?		
3) ... was your sleep restless?		
4) ... were you happy?		
5) ... did you feel lonely?		
6) ... did you enjoy life?		
7) ... did you feel sad?		
8) ... were you unable to get going?		

CASP-19
(Hyde M., et al., A measure of quality of life in early old age: the theory, development and properties of a needs satisfaction model (CASP-19). Aging Ment Health. 2003;7(3):186–94).

	Often (0)	Sometimes (1)	Not often (2)	Never (3)
**1** My age prevents me from doing the things I would like to				
**2** I feel that what happens to me is out of my control				
**3** I feel free to plan for the future				
**4** I feel left out of things				
**5** I can do the things I want to do				
**6** Family responsibilities prevent me from doing what I want to do				
**7** I feel that I can please myself what I do				
**8** My health stops me from doing things I want to				
**9** Shortage of money stops me from doing the things I want to do				
**10** I look forward to each day				
**11** I feel that my life has meaning				
**12** I enjoy the things that I do				
**13** I enjoy being in the company of others				
**14** On balance, I look back on my life with a sense of happiness				
**15** I feel full of energy these days				
**16** I choose to do things that I have never done before				
**17** I am satisfied with the way my life has turned out				
**18** I feel that life is full of opportunities				
**19** I feel that the future looks good for me				

For peer review only

STROBE 2007 (v4) Statement—Checklist of items that should be included in reports of *cohort studies*

Section/Topic	Item #	Recommendation	Reported on page #
**Title and abstract**	1	(*a*) Indicate the study's design with a commonly used term in the title or the abstract	1,2
		(*b*) Provide in the abstract an informative and balanced summary of what was done and what was found	2
**Introduction**			
Background/rationale	2	Explain the scientific background and rationale for the investigation being reported	3-4
Objectives	3	State specific objectives, including any prespecified hypotheses	5
**Methods**			
Study design	4	Present key elements of study design early in the paper	5
Setting	5	Describe the setting, locations, and relevant dates, including periods of recruitment, exposure, follow-up, and data collection	5
Participants	6	(*a*) Give the eligibility criteria, and the sources and methods of selection of participants. Describe methods of follow-up	5
		(*b*) For matched studies, give matching criteria and number of exposed and unexposed	
Variables	7	Clearly define all outcomes, exposures, predictors, potential confounders, and effect modifiers. Give diagnostic criteria, if applicable	5-8
Data sources/ measurement	8*	For each variable of interest, give sources of data and details of methods of assessment (measurement). Describe comparability of assessment methods if there is more than one group	5-8
Bias	9	Describe any efforts to address potential sources of bias	
Study size	10	Explain how the study size was arrived at	5 & 9
Quantitative variables	11	Explain how quantitative variables were handled in the analyses. If applicable, describe which groupings were chosen and why	8
Statistical methods	12	(*a*) Describe all statistical methods, including those used to control for confounding	8
		(*b*) Describe any methods used to examine subgroups and interactions	
		(*c*) Explain how missing data were addressed	9
		(*d*) If applicable, explain how loss to follow-up was addressed	
		(*e*) Describe any sensitivity analyses	8-9
**Results**			

For peer review only

Participants	13*	(a) Report numbers of individuals at each stage of study—eg numbers potentially eligible, examined for eligibility, confirmed eligible, included in the study, completing follow-up, and analysed	9
		(b) Give reasons for non-participation at each stage	9
		(c) Consider use of a flow diagram	
Descriptive data	14*	(a) Give characteristics of study participants (eg demographic, clinical, social) and information on exposures and potential confounders	9
		(b) Indicate number of participants with missing data for each variable of interest	9
		(c) Summarise follow-up time (eg, average and total amount)	
Outcome data	15*	Report numbers of outcome events or summary measures over time	10 and Table 1
Main results	16	(*a*) Give unadjusted estimates and, if applicable, confounder-adjusted estimates and their precision (eg, 95% confidence interval). Make clear which confounders were adjusted for and why they were included	10 and Tables 2-5
		(*b*) Report category boundaries when continuous variables were categorized	6-7
		(*c*) If relevant, consider translating estimates of relative risk into absolute risk for a meaningful time period	
Other analyses	17	Report other analyses done—eg analyses of subgroups and interactions, and sensitivity analyses	10
**Discussion**			
Key results	18	Summarise key results with reference to study objectives	10
**Limitations**			
Interpretation	20	Give a cautious overall interpretation of results considering objectives, limitations, multiplicity of analyses, results from similar studies, and other relevant evidence	11-12
Generalisability	21	Discuss the generalisability (external validity) of the study results	12
**Other information**			
Funding	22	Give the source of funding and the role of the funders for the present study and, if applicable, for the original study on which the present article is based	17

*Give information separately for cases and controls in case-control studies and, if applicable, for exposed and unexposed groups in cohort and cross-sectional studies.

Note: An Explanation and Elaboration article discusses each checklist item and gives methodological background and published examples of transparent reporting. The STROBE checklist is best used in conjunction with this article (freely available on the Web sites of PLoS Medicine at http://www.plosmedicine.org/, Annals of Internal Medicine at http://www.annals.org/, and Epidemiology at http://www.epidem.com/). Information on the STROBE Initiative is available at www.strobe-statement.org.

For peer review only

BMJ Open

The role of stress and health behaviour in linking weight discrimination and health: a secondary data analysis in England

Journal:	*BMJ Open*
Manuscript ID	bmjopen-2023-072043.R2
Article Type:	Original research
Date Submitted by the Author:	25-Aug-2023
Complete List of Authors:	Hackett, Ruth; King's College London, Department of Psychology<br>Jackson, Sarah ; University College London, Epidemiology and Public Health<br>Corker, Elizabeth; University College London, Clinical; University of Sheffield, Department of Psychology<br>Steptoe, Andrew; University College London, Department of Behavioural Science and Health
**Primary Subject Heading**:	Epidemiology
Secondary Subject Heading:	Public health
Keywords:	EPIDEMIOLOGY, PUBLIC HEALTH, Depression & mood disorders < PSYCHIATRY

SCHOLARONE™
Manuscripts

I, the Submitting Author has the right to grant and does grant on behalf of all authors of the Work (as defined in the below author licence), an exclusive licence and/or a non-exclusive licence for contributions from authors who are: i) UK Crown employees; ii) where BMJ has agreed a CC-BY licence shall apply, and/or iii) in accordance with the terms applicable for US Federal Government officers or employees acting as part of their official duties; on a worldwide, perpetual, irrevocable, royalty-free basis to BMJ Publishing Group Ltd ("BMJ") its licensees and where the relevant Journal is co-owned by BMJ to the co-owners of the Journal, to publish the Work in this journal and any other BMJ products and to exploit all rights, as set out in our licence.

The Submitting Author accepts and understands that any supply made under these terms is made by BMJ to the Submitting Author unless you are acting as an employee on behalf of your employer or a postgraduate student of an affiliated institution which is paying any applicable article publishing charge ("APC") for Open Access articles. Where the Submitting Author wishes to make the Work available on an Open Access basis (and intends to pay the relevant APC), the terms of reuse of such Open Access shall be governed by a Creative Commons licence – details of these licences and which Creative Commons licence will apply to this Work are set out in our licence referred to above.

Other than as permitted in any relevant BMJ Author's Self Archiving Policies, I confirm this Work has not been accepted for publication elsewhere, is not being considered for publication elsewhere and does not duplicate material already published. I confirm all authors consent to publication of this Work and authorise the granting of this licence.

For peer review only

The role of stress and health behaviour in linking weight discrimination and health: a secondary data analysis in England

Ruth A. Hackett[1,2], Sarah E. Jackson[2], Elizabeth Corker[3,4], Andrew Steptoe[2]

[1] Health Psychology Section, Department of Psychology, Institute of Psychiatry, Psychology and Neuroscience, King's College London, London, UK

[2] Department of Behavioural Science and Health, University College London, London, UK

[3] Clinical, Educational and Health Psychology, University College London, London, UK

[4] Clinical and Applied Psychology Unit, Department of Psychology, University of Sheffield, Sheffield, UK

Running head: Weight discrimination, health and wellbeing

Corresponding author: Dr Ruth A Hackett, Health Psychology Section, Department of Psychology, Institute of Psychiatry, Psychology and Neuroscience, King's College London, Great Maze Pond, London SE1 9RT, UK. ruth.hackett@kcl.ac.uk +44 (0) 207 188 5410

Competing interests: None

Word count: 4,396

For peer review only - http://bmjopen.bmj.com/site/about/guidelines.xhtml

Abstract

Objective: To examine the role of stress and health-risk behaviours in relationships between weight discrimination and health and wellbeing.

Design: Secondary data analysis of an observational cohort study

Setting: The English Longitudinal Study of Ageing

Participants: Data were from 4,341 adults (≥50y) with overweight/obesity.

Primary Outcome Measures: We tested associations between perceived weight discrimination at baseline (2010/11) and self-rated health, limiting long-standing illness, depressive symptoms, quality of life, and life satisfaction over four-year follow-up (2010/11; 2014/15). Potential mediation by stress exposure (hair cortisol) and health-risk behaviours (smoking, physical inactivity, alcohol consumption) was assessed.

Results: Cross-sectionally, perceived weight discrimination was associated with higher odds of fair/poor self-rated health (OR=2.05[95%CI 1.49;2.82]), limiting long-standing illness (OR=1.76[1.29;2.41]) and depressive symptoms (OR=2.01[1.41;2.85]), and lower quality of life (*B*=-5.82[95%CI -7.01;-4.62]) and life satisfaction (*B*=-2.36[-3.25;-1.47]). Prospectively, weight discrimination was associated with higher odds of fair/poor self-rated health (OR=1.63[1.10;2.40]) and depressive symptoms (OR=2.37[95%CI 1.57;3.60]) adjusting for baseline status. Those who reported discrimination had higher hair cortisol concentrations (*B*=0.14[0.03;0.25]) and higher odds of physical inactivity (OR=1.90[1.18;3.05]). These variables did not significantly mediate associations between discrimination and health outcomes.

Conclusions: Weight discrimination is associated with poor health and wellbeing. While this discrimination is associated with stress exposure and physical inactivity, these variables explain little of the association between discrimination and poorer outcomes.

Key words: weight discrimination; stigma; obesity; physical activity; smoking; health; wellbeing

For peer review only - http://bmjopen.bmj.com/site/about/guidelines.xhtml

Strengths and limitations

- Secondary data analysis of a large prospective observational study.
- Assessment of multiple health and wellbeing outcomes.
- The prevalence of weight discrimination in the sample was low (which limits generalisability) and it was only assessed on one occasion increasing the risk of misclassification bias.
- Measures of health behaviour were self-reported increasing the risk of measurement error and there was no data on diet (pattens or quality) or eating behaviour (e.g., dieting, disordered eating)
- The sample was compromised of mostly white middle aged and older adults which limits generalisability.

For peer review only

Introduction

The average person in the United Kingdom (UK) carries excess weight (1,2). Recent decades have seen an increase in the proportion of adults with a body mass index (BMI) in the overweight (25-29.9kg/m^2) or obesity (≥30kg/m^2) range, and a shift in the distribution of cases such that a greater proportion have 'morbid' obesity (BMI ≥40kg/m^2) (3). Despite the increasingly normative nature of obesity, it is widely stigmatised with those with obesity characterised as lazy, unintelligent, and lacking in self-discipline (4). These stereotypes translate into weight discrimination across contexts and interactions, including the workplace, education, healthcare settings, and interpersonal relationships (4).

Mounting evidence suggests that weight discrimination can impact health and wellbeing (5,6). Studies have linked perceived weight discrimination to adverse outcomes, including poorer self-rated health, greater disease burden, functional disability, psychiatric comorbidity, psychosocial stress, loneliness, poorer life satisfaction, and increased risk of premature death (7–13). Evidence is suggestive of a prospective relationship (i.e. discrimination preceding a decline in health) which persists after adjustment for BMI (7,9). It is of interest to analyse these issues in middle-aged and older people, among whom disease and disability risk is elevated.

Several mechanisms may underpin the relationship between perceived weight discrimination and poorer health and wellbeing. Prominent theories focus on the role of (i) stress responses to discrimination and (ii) health-risk behaviours as a means of coping with or avoiding discrimination (14). Experiencing weight discrimination can be stressful, both physiologically, in the form of cortisol responses (15,16), and psychologically, in the form of increased negative emotion (e.g. psychological distress, depressive symptoms, and fear, among others) (8). If an individual perceives discrimination regularly, as is common with weight stigma (17), stress responses will be activated frequently, potentially leading to chronically elevated cortisol and a negative emotional state (e.g., depression, anxiety, ongoing feelings of stress etc.) . This in turn lead to ill health under allostatic load theory (18). Indeed, observational studies have associated elevated cortisol levels and weight discrimination (19,20).

Health-risk behaviours may also link weight discrimination to poorer health and wellbeing. These may emerge as a biological response to discrimination, for example, hypothalamic-pituitary-adrenal (HPA) axis activation upregulating appetite and the drive to consume energy-dense 'comfort' foods (21–23). Indeed, disordered eating and binge eating disorder (eating large amounts accompanied by feelings of distress) is common at higher weight ranges (24) and has been associated with weight discrimination (25). In this way, weight stigma

For peer review only - http://bmjopen.bmj.com/site/about/guidelines.xhtml

may contribute to a vicious cycle of stress, weight gain and further discrimination (14). Unhealthy behaviours may also provide a coping mechanism: food, nicotine, and alcohol activate dopaminergic reward pathways in the brain (26) and may help to alleviate the negative psychological impact of discrimination. Avoiding activities for fear of discrimination (e.g. exercising in public places) may also act as a barrier to a healthy lifestyle (27). To our knowledge, no studies have investigated the extent to which these variables mediate associations between weight-based discrimination and health and wellbeing outcomes.

The present study used data from English Longitudinal Study of Ageing (ELSA) to examine (i) cross-sectional and prospective associations between perceived weight discrimination and health and wellbeing (self-rated health, limiting long-standing illness, depressive symptoms, quality of life, and life satisfaction) and (ii) whether these associations were mediated by stress exposure and health-risk behaviours (smoking, physical inactivity, and alcohol consumption).

For peer review only

Methods

Study population

ELSA is a representative longitudinal study of adults aged ≥50 living in England (28). Established in 2002, ELSA collects data via computer-assisted personal interviews and self-completion questionnaires biennially, with nurse visits in alternate waves to collect objective health measures, including BMI.

We used data on perceived weight discrimination from Wave 5 (2010/11; the only wave in which discrimination was assessed); hair cortisol, health behaviours, and BMI from Wave 6 (2012/13), and health and wellbeing from Waves 5 and 7 (2014/15). We restricted our sample to those with a BMI in the overweight or obese range (≥25kg/m^2).

Ethical approval for ELSA was obtained from the London Multicentre Research and Ethics Committee (MREC/01/02/91). All participants provided informed consent. Data are freely available to download from the g2aging.com (29).

Patient and public involvement

For peer review only - http://bmjopen.bmj.com/site/about/guidelines.xhtml

No patient involved.

Measures

All questionnaire measures used in the study can be found in Supplementary material.

Exposure: perceived weight discrimination

Our measure of perceived discrimination was based on items developed and used in other prospective cohort studies (e.g. Health and Retirement Study; Midlife Development in the United States) (30). Participants were asked how often they encounter five discriminatory situations: *"In your day-to-day life, how often have any of the following things happened to you: 1) you are treated with less respect or courtesy; 2) you receive poorer service than other people in restaurants and stores; 3) people act as if they think you are not clever; 4) you are threatened or harassed; and 5) you receive poorer service or treatment than other people from doctors or hospitals."*. Response options were on a 5-point scale ranging from "*never*" to "*almost every day*." Because data were skewed, with most participants reporting no discrimination, we dichotomised responses to indicate whether or not participants had experienced discrimination in the past year (a few times or more a year vs. less than once a year or never), with the exception of the fifth item which was dichotomised to indicate whether or not respondents had ever experienced discrimination from doctors or hospitals (never vs. all other options) as most participants never reported discrimination in this setting (31–34).

A follow-up question asked participants who reported discrimination to indicate the reason(s) they attributed to their experience (e.g., weight, age, sex, race, sexual orientation, disability etc.,) (31–34). Those who attributed any experience of discrimination to their weight are treated as cases of perceived weight discrimination as in other studies (19,35–39).

Outcomes: health and wellbeing

Self-rated health was assessed using a single item: *"Would you say your health is… poor/fair/good/very good/excellent?"* We analysed the proportion of individuals rating their health as fair/poor, as in other investigations (31,40). As a sensitivity check, analyses were also conducted using the continuous range of scores.

Limiting long-standing illness was assessed with two questions: 1) *"Do you have any long-standing illness, disability, or infirmity? By long-standing I mean anything that has troubled you over a period of time or that*

For peer review only - http://bmjopen.bmj.com/site/about/guidelines.xhtml

is likely to affect you over a period of time." Those who responded yes were asked: 2) "*Does this illness or disability limit your activities in any way?*" Those who responded yes to both items were classed as having a limiting long-standing illness.

Depressive symptoms were assessed with the eight-item Center for Epidemiologic Studies Depression Scale (41). Respondents were asked if they had experienced depressive symptoms (e.g. restless sleep and being unhappy) over the past month using a binary (yes/no) response. Total scores ranged from 0 to 8. Data were dichotomised using an established cut-off (scores ≥4) (42). As a sensitivity check, analyses were also conducted using the continuous range of scores.

Quality of life was assessed with the CASP-19 (43). Items covered four domains of quality of life; control (e.g., '*I feel that what happens to me is out of my control*'), autonomy (e.g., '*My health stops me from doing things I want to do*'), self-realisation (e.g., '*I feel that life is full of opportunities*'), and pleasure (e.g., '*I enjoy being in the company of others*'). Respondents were asked how often each statement applies to them (often=0, sometimes=1, not often=2, never=3). Positively-worded items were reverse scored so that a higher total score indicated higher quality of life (range: 0–57).

Life satisfaction was assessed with the 5-item Satisfaction With Life Scale (44), which asks the extent to which participants agree with statements including '*In most ways my life is close to my ideal*'; 'and '*I am satisfied with my life*';. Responses ranged from 0 (strongly disagree) to 6 (strongly agree) and were summed to produce a total score of 0-30.

Mediators: stress exposure and health-risk behaviours

The hair cortisol assessment procedure has been described in detail elsewhere (45). Briefly, a scalp-proximal hair sample at least 2cm long and weighing at least 10mg was taken from the posterior vertex, cut as close to the scalp as possible. The wash procedure and steroid extraction were undertaken using high performance liquid chromatography–mass spectrometry. Assuming an average hair growth of 1cm per month the 2cm hair segment represents average cortisol accumulated two months prior to sampling.

Smoking status was assessed with the question "*Do you smoke cigarettes at all nowadays?*" (yes/no).

Physical activity was self-reported in response to three questions on the frequency of participation in light, moderate and vigorous activities (more than once a week/once a week/one to three times a month/hardly

For peer review only - http://bmjopen.bmj.com/site/about/guidelines.xhtml

ever or never). We compared participants who were physically inactive (defined as no activity on a weekly basis) with those who reported engaging in light, moderate, or vigorous activity at least once a week.

Alcohol consumption was assessed with the question "*On how many days out of the last seven did you have an alcoholic drink?*". Those who responded 'five', 'six' or 'seven' days were classified as frequent alcohol drinkers. We compared frequent alcohol drinkers with all other categories combined.

Covariates

Participants self-reported their age, sex, and ethnicity (white, other). Socioeconomic status was indexed with a measure of household non-pension wealth (28)), divided into quintiles across the entire Wave 5 sample. BMI (m^2/kg) was based on objective height and weight measurements.

Statistical analysis

Associations between perceived weight discrimination and covariates were examined using one-way independent analysis of variance (ANOVA) for continuous variables and χ^2 tests for categorical variables.

For our primary analyses, we used multivariable regression models (logistic for categorical outcomes, linear for continuous outcomes) to explore cross-sectional and prospective associations between perceived weight discrimination and health and wellbeing. All models were adjusted for age, sex, ethnicity, wealth, and BMI. Prospective models were additionally adjusted for baseline status/score on the outcome variable.

Where perceived weight discrimination was significantly associated with an outcome in prospective analyses, we tested for mediation by stress exposure and health-risk behaviours (Figure 1). Hair cortisol data was log transformed to correct skewness. Establishing mediation requires the mediator to be associated with the exposure (path *a*) and the outcome (path *b*), so we first tested associations of (i) perceived weight discrimination with (a) stress exposure (hair cortisol concentration) using linear regression and (b) health-risk behaviours (smoking, physical inactivity, and alcohol consumption) using logistic regression; and (ii) stress exposure and health-risk behaviours with health and wellbeing outcomes using logistic or linear regression, as appropriate. Where associations were significant, we used the *sgmediation* command in STATA, which calculates total (path *c*), direct (path *c*′), and indirect (path $a \times b$) effects, and tests the significance of the indirect effect using the Sobel test. We used bootstrapping with 5,000 sampling replications to estimate the 95% confidence interval and calculated effect ratios reflecting the proportion of the total effect of the

For peer review only - http://bmjopen.bmj.com/site/about/guidelines.xhtml

independent variable on the dependent variable that is explained by the mediator. Mediation models were adjusted for age, sex, ethnicity, wealth, BMI, and status/score on the outcome variable at baseline.

We conducted three sensitivity analyses. In the first we expanded our analytic sample to include participants who provided data on perceived weight discrimination but did not have a BMI ≥25m²/kg. We assessed whether the pattern of associations between perceived weight discrimination and health and wellbeing outcomes was similar when including these participants. For our second sensitivity analysis we restricted our analytic sample to participants with BMI ≥30m²/kg and assessed whether the associations between perceived weight discrimination and health and wellbeing outcomes remained for those participants in the higher BMI ranges. For the final sensitivity analysis, we changed the frequency weight discrimination was encountered from *'in the past year vs less than once a year/never'* (main analysis) to whether the participant *ever* experienced discrimination (*'never' vs 'almost every day; at least once a week; a few times a month; a few times a year; less than once a year'*). We assessed whether our results changed when using this different definition of weight discrimination. Analyses were performed on complete cases using SPSS v.25, with the exception of the mediation models which were run in STATA v.13.

Results

In Wave 5, 9,090 participants (77.5% of those eligible) took part in the face-to-face interview, of whom 8,107 (93%) returned the self-completion questionnaire. We excluded 2,142 participants with missing data on weight discrimination or covariates (including BMI) and 1,624 with BMI <25kg/m². Our final sample for cross-sectional analysis comprised of 4,341 people, aged 52-89 years with a BMI 25.0-59.4kg/m².

Perceived weight discrimination was reported by 210 (4.8%) respondents, who were on average younger, heavier, more socioeconomically disadvantaged and more likely to be female than those who did not report weight discrimination (Table 1). Of the participants who perceived weight discrimination, 44 (21%) also reported sex discrimination, 40 (19%) reported disability discrimination, 11 (5.2%) reported race discrimination and 6 (2.9%) reported sexuality discrimination.

For peer review only - http://bmjopen.bmj.com/site/about/guidelines.xhtml

**Table 1** Sample characteristics at baseline (Wave 5)					
		**Whole sample (*n*=4341)**	**Perceived weight discrimination (*n*=210)**	**No perceived weight discrimination (*n*=4131)**	***p***
Age (years), mean (SD)		66.62 (7.96)	62.04 (6.58)	66.85 (7.95)	<0.001
Sex, % (*n*)					
	Men	47.2 (2051)	34.8 (73)	47.9 (1978)	<0.001
	Women	52.8 (2290)	65.2 (137)	52.1 (2153)	-
Ethnicity, % (*n*)					
	White	97.9 (4250)	97.6 (205)	97.9 (4045)	0.768
	Other	2.1 (91)	2.4 (5)	2.1 (86)	-
Wealth quintile, % (*n*)					
	1 (poorest)	15.5 (672)	26.7 (56)	14.9 (616)	<0.001
	2	20.6 (896)	29.0 (61)	20.2 (835)	-
	3	21.0 (913)	18.6 (39)	21.2 (874)	-
	4	21.8 (946)	16.2 (34)	22.1 (912)	-
	5 (richest)	21.1 (914)	9.5 (20)	21.6 (894)	-
BMI (kg/m^2), mean (SD)		30.37 (4.47)	36.63 (6.17)	30.05 (4.12)	<0.001
BMI, body mass index.					

Cross-sectionally, perceived weight discrimination was associated with significantly higher odds of fair/poor self-rated health, limiting long-standing illness, and depressive symptoms, and lower mean ratings of quality of life and life satisfaction, after adjustment for age, sex, ethnicity, wealth, and BMI (Table 2). Prospectively, perceived weight discrimination was associated with significantly higher odds of future fair/poor self-rated health and depressive symptoms, but differences in limiting long-standing illness, quality of life, and life satisfaction were not statistically significant after adjustment for covariates including baseline status/score on the outcome variable (Table 2). Analysing depression and self-rated health as continuous variables did not change the pattern of results (Table 2).

For peer review only - http://bmjopen.bmj.com/site/about/guidelines.xhtml

Table 2 Cross-sectional and prospective associations of perceived weight discrimination with health and wellbeing outcomes (path *c* on Figure 1)

	Cross-sectional (Wave 5)		Prospective (Wave 7)	
	No perceived weight discrimination	Perceived weight discrimination	No perceived weight discrimination	Perceived weight discrimination
Fair/poor self-rated health				
n included in analysis	4130	208	3705	188
% (*n*) reporting outcome	22.5 (928)	48.1 (100)	26.3 (973)	53.7 (101)
Adjusted OR [95% CI]<br>*p*	1.00 (ref)	2.05 [1.49; 2.82]<br><0.001	1.00 (ref)	1.63 [1.10; 2.40]<br>0.014
Self-rated health (range 1-5)				
n included in analysis	4130	208	3705	188
Mean (SD) score	2.73 (1.04)	3.47 (1.06)	2.85 (1.05)	3.56 (0.99)
Adjusted *B* [95% CI]<br>*p*	Ref	0.44 [0.29; 0.58]<br><0.001	Ref	0.14 [0.02; 0.27]<br>0.026
Limiting long-standing illness				
n included in analysis	4129	209	3740	190
% (*n*) reporting outcome	32.2 (1330)	55.0 (115)	36.2 (1354)	56.8 (108)
Adjusted OR [95% CI]<br>*p*	1.00 (ref)	1.76 [1.29; 2.41]<br><0.001	1.00 (ref)	1.24 [0.85; 1.79]<br>0.268
Depressive symptoms				
n included in analysis	4098	210	3643	188
% (*n*) reporting outcome	11.9 (487)	28.6 (60)	10.8 (392)	29.8 (56)
Adjusted OR [95% CI]<br>*p*	1.00 (ref)	2.01 [1.41; 2.85]<br><0.001	1.00 (ref)	2.37 [1.57; 3.60]<br><0.001
Depressive symptoms (range 0-8)				
n included in analysis	4098	210	3643	188
Mean (SD) score	1.30 (1.78)	2.60 (2.42)	1.24 (1.73)	2.31 (2.12)
Adjusted *B* [95% CI]<br>*P*	Ref	0.94 [0.68; 1.20]<br><0.001	Ref	0.38 [0.15; 0.62]<br><0.001
Quality of life (range 0-57)				
n included in analysis	3942	203	3106	154
Mean (SD) score	41.72 (8.36)	34.24 (9.57)	42.19 (8.47)	36.56 (9.34)
Adjusted *B* [95% CI]<br>*p*	Ref	-5.82 [-7.01; -4.62]<br><0.001	Ref	0.01 [-0.96; 0.97]<br>0.992
Life satisfaction (range 0-30)				
n included in analysis	4008	199	3240	160
Mean (SD) score	21.05 (6.02)	17.45 (7.62)	21.22 (6.03)	18.46 (7.31)
Adjusted *B* [95% CI]	Ref	-2.36 [-3.25; -1.47]	Ref	-0.15 [-0.89; 0.59]

For peer review only - http://bmjopen.bmj.com/site/about/guidelines.xhtml

	p		<0.001			0.691

OR, odds ratio. CI, confidence interval. SD, standard deviation.
ORs, *B*s and 95% CIs are adjusted for age, sex, ethnicity, wealth, and body mass index. Prospective results are additionally adjusted for status/score on the outcome variable at baseline (Wave 5).
Ref = reference category.

Table 3 Associations of perceived weight discrimination at Wave 5 with health behaviours and stress exposure at Wave 6 (path *a* on Figure 1)

	**No perceived weight discrimination**	**Perceived weight discrimination**
Smoking status		
n included in analysis	4131	210
% (*n*) current smoker	8.4 (347)	14.3 (30)
Adjusted OR [95% CI]	1.00 (ref)	1.43 [0.91-2.25]
p		0.120
Physical inactivity		
n included in analysis	4131	210
% (*n*) physically inactive	5.9 (242)	13.8 (29)
Adjusted OR [95% CI]	1.00 (ref)	1.90 [1.18; 3.05]
p		0.008
Alcohol consumption		
n included in analysis	3890	186
% (*n*) frequent alcohol consumption	20.6 (801)	12.9 (24)
Adjusted OR [95% CI]	1.00 (ref)	0.93 [0.59; 1.49]
p		0.774
Hair cortisol concentration		
n included in analysis	2456	128
Mean (SD) log pg/mg	0.90 (0.57)	1.09 (0.65)
Adjusted *B* [95% CI]	Ref	0.14 [0.03; 0.25]
p		0.012

OR, odds ratio. CI, confidence interval.
ORs and 95% CIs are adjusted for age, sex, ethnicity, wealth, and body mass index.

We assessed whether prospective associations observed between perceived weight discrimination and self-rated health and depressive symptoms were mediated by stress exposure and health-risk behaviours (Figure 1; as limiting long-standing illness, quality of life, and life satisfaction were not prospectively associated with discrimination these were not investigated). After adjustment for covariates, participants who reported weight discrimination in Wave 5 had significantly higher hair cortisol concentrations (indicative of stress exposure) and higher odds of physically inactivity in Wave 6 than those who did not (Table 3; path *a* Figure

For peer review only - http://bmjopen.bmj.com/site/about/guidelines.xhtml

1). No association with smoking or alcohol consumption was observed (Table 3). Physical inactivity in Wave 6 was significantly associated with higher odds of fair/poor self-rated health and depressive symptoms in Wave 7 (Table 4; path *b* Figure 1). While hair cortisol concentrations in Wave 6 were not significantly associated with depressive symptoms in Wave 7 (Table 4; path *b* Figure 1), each one-unit increase in hair cortisol concentration (log pg/mg) was associated with 17% higher odds of subsequently reporting fair/poor self-rated health, which while not quite statistically significant (p=0.066) warranted further investigation as a potential mediator.

Table 4 Associations of level of physical activity and stress exposure at Wave 6 with health and wellbeing outcomes (prospectively associated with perceived weight discrimination) at Wave 7 (path *b* on Figure 1)

	Fair/poor self-rated health			Depressive symptoms	
	**No**	**Yes**		**No**	**Yes**
Physical inactivity					
n included in analysis	2821	1075		3404	456
% (*n*) physically inactive	3.4 (96)	12.4 (133)		5.1 (173)	11.4 (52)
Adjusted OR [95% CI]<br>*p*	1.00 (ref)	2.57 [1.92; 3.45]<br><0.001		1.00 (ref)	1.71 [1.21; 2.42]<br>0.002
Hair cortisol concentration					
n included in analysis	1699	623		2008	292
Mean (SD) log pg/mg	0.89 (0.57)	0.97 (0.58)		0.91 (0.57)	0.91 (0.57)
Adjusted OR [95% CI]<br>*p*	1.00 (ref)	1.17 [0.99; 1.38]<br>0.066		1.00 (ref)	0.93 [0.74; 1.17]<br>0.538

OR, odds ratio. CI, confidence interval.
ORs and 95% CIs are adjusted for age, sex, ethnicity, wealth, and body mass index.
Ref = reference category.

Results of mediation analyses are summarised in Table 5 (path *c*, path *c'*, and indirect effects Figure 1). There was no evidence of mediation, with effect ratios indicating that differences in physical inactivity explained approximately 4.2% of the association between perceived weight discrimination and self-rated health and 1.5% of the association between perceived weight discrimination and depressive symptoms, and differences in hair cortisol concentrations explained 1.5% of the association between perceived weight discrimination and self-rated health. None of these indirect effects were statistically significant.

For peer review only - http://bmjopen.bmj.com/site/about/guidelines.xhtml

Table 5 Models testing mediation of the associations between perceived weight discrimination and fair/poor self-rated health and depressive symptoms by level of physical activity and stress exposure (see Figure 1)

	Mediation by physical inactivity				
	**Coeff.**	**SE**	***p****	**Bootstrap 95% CI**	**Effect ratio**
**Fair/poor self-rated health**					
Total effect (path *c*)	0.081	0.030	0.007	-	-
Direct effect (path *c'*)	0.077	0.030	0.009	-	-
Indirect effect (via mediator)	0.003	0.002	0.062	-0.0002; 0.009	0.042
**Depressive symptoms**					
Total effect (path *c*)	0.113	0.023	<0.001	-	-
Direct effect (path *c'*)	0.111	0.023	<0.001	-	-
Indirect effect (via mediator)	0.002	0.001	0.169	-0.001; 0.005	0.015
	**Mediation by hair cortisol concentration**				
**Fair/poor self-rated health**					
Total effect (path *c*)	0.080	0.038	0.034	-	-
Direct effect (path *c'*)	0.079	0.038	0.037	-	-
Indirect effect (via mediator)	0.001	0.878	0.380	-0.001; 0.005	0.015

Analyses are adjusted for age, sex, ethnicity, wealth, body mass index, and status on the outcome variable at baseline (Wave 5).
Coeff. = coefficient; SE = standard error; CI = confidence interval; BMI = body mass index.
**p* values shown for indirect effects are derived from the Sobel test for consistency with total and direct effects, however bootstrap 95% confidence intervals provide a more robust indication of significant mediation (see Method for more details).

For peer review only

Sensitivity analyses

In the first sensitivity analysis, we expanded our sample to include participants who provided data on perceived weight discrimination but did not have a BMI in the overweight/obesity range (see Supplementary Table 1 for sample characteristics). We assessed whether the pattern of associations between weight discrimination and health and wellbeing outcomes was similar when including these participants (Supplementary Table 2). The results remained unchanged when including participants who did not have overweight/obesity.

In the second sensitivity analysis, we restricted the sample to participants with BMI ≥30m^2/kg (see Supplementary Table 3 for the characteristics of this restricted sample). We assessed whether the observed relationships between perceived weight discrimination and health and wellbeing outcomes were similar in these participants in the higher BMI ranges. As can be seen the results were similar when restricting the sample to those with ≥30m^2/kg (see Supplementary Table 4).

For our final sensitivity analysis, we changed the frequency weight discrimination was encountered from *at least once a year* (main analysis) to whether the participant *ever* experienced discrimination (*'never' vs all other options*). Ever encountering weight discrimination had a similar impact on cross-sectional and prospective health and wellbeing outcomes (Supplementary Table 5).

Discussion

In this large cohort of older adults, cross-sectionally, perceived weight discrimination was associated with higher odds of fair/poor self-rated health, limiting long-standing illness, and depressive symptoms, and lower quality of life and life satisfaction. Prospectively, perceived weight discrimination was associated with higher odds of fair/poor self-rated health and depressive symptoms after adjustment for baseline status. All associations were independent of age, sex, ethnicity, wealth, and BMI. However, while those who reported weight discrimination had higher odds of physically inactivity and higher hair cortisol concentrations than those who did not, there was no evidence that these variables mediated associations between perceived weight discrimination and poor health and wellbeing.

For peer review only - http://bmjopen.bmj.com/site/about/guidelines.xhtml

Our finding that perceived weight discrimination is associated with poorer health and wellbeing is consistent with previous studies (7–11). While our results provide prospective evidence linking perceived weight discrimination with self-rated health and depressive symptoms, as has been observed in other samples (7,8,11), we failed to replicate a prospective association with life satisfaction (7) or detect any prospective association with limiting long-standing illness or quality of life. It is unclear whether this is the result of differences in measurement (e.g., assessing limiting long-standing illness vs total disease burden), sample (e.g., age range, the lack of ethnic diversity in the current sample), setting (i.e., England versus the US), or covariates used (highlighting the possibility of uncontrolled confounding). Nonetheless, taking our findings in the context of the literature, it is evident that weight stigma is a social determinant of health and a driver of health inequalities (46). As such, it is not only a social justice issue, but also a public health issue. Further, we observed an inverse association between perceived weight discrimination and wealth, suggesting that those of lower socioeconomic position could be more vulnerable to the deleterious effects of weight discrimination. This inverse association is in line with previous reports in ELSA, but not other studies (47). Therefore, the role of socioeconomic position in the associations between weight discrimination, health and wellbeing, warrants further attention.

A key study aim was to explore the role of stress and health-risk behaviours as pathways underpinning associations between weight discrimination and health and wellbeing (48). In line with previous findings, perceived weight discrimination was associated with increased stress (higher hair cortisol concentrations) and physical inactivity (19,20), but these variables explained only a very small and non-significant proportion of the total associations of perceived weight discrimination with self-rated health and depressive symptoms. Associations with smoking and alcohol consumption were not statistically significant, so these variables were not considered plausible mediators. There are several potential explanations for these findings. The variables we assessed make only small contributions to the impact of weight discrimination on health and wellbeing, and there are other variables more important to this relationship that we were unable to account for in our modelling. We had no information on dietary factors or eating behaviours such as unsupervised and/or unsafe dieting practices, binge eating, disordered eating, and weight cycling. Therefore, we were unable to explore these as potential mediators. Given evidence that people who experience weight discrimination are at increased risk of overeating, disordered eating (49,50) and have a greater risk of binge eating disorder (25) than those who do not report discrimination these present important unexamined pathways from weight discrimination to poor health. It is plausible that the use of maladaptive dietary and eating behaviours as a means of coping with weight discrimination could lead to a vicious cycle of further distress, weight gain and

For peer review only - http://bmjopen.bmj.com/site/about/guidelines.xhtml

disproportionate burden of weight discrimination (14,25). This represents an important avenue of further research, particularly considering the lack of mediation by other factors in the current study. Future prospective studies should explicitly set out to measure the role of diet, dieting behaviour, weight cycling, and disordered eating (such as binge eating disorder) to understand how these factors are involved in the relationship between weight discrimination and health. This is of relevance in both community settings (as in this study) and in healthcare settings. There is evidence that healthcare providers can hold negative attitudes and believe stereotypes about people with overweight/obesity (51,52). This can have negative implications for both the quality of care received and can result in people avoiding healthcare settings for fear of encountering weight discrimination (52). This is of relevance to the current study as we observed a cross-sectional association between perceived weight discrimination and reports of limiting longstanding illness. Indeed, people with obesity-related health conditions (such as diabetes or heart disease) have more frequent encounters with healthcare professionals than those without these conditions. The exploration of healthcare settings as a location where weight discrimination could be encountered represents an important area for future work.

Unmeasured variables include negative emotions such as psychological distress and feelings of anxiety which could be associated with weight discrimination. It is possible that these emotions could mediate the link between perceived weight discrimination and health, or these could be viewed as negative outcomes in their own right. Social isolation and loneliness play a role in weight-related conditions(53,54). Changes in social contact or relationships could occur as a result of perceiving weight discrimination. The relationship between weight discrimination, indicators of social connectedness and health remain to be teased out. We included self-rated health as an outcome in this study, as this is considered to better and more broadly capture 'health' than most other indicators (55). However, the association between perceived weight discrimination and specific health conditions (including weight-related conditions) represents an important area for future work. Another explanation for the non-significant mediation observed in the current study is that the variables we analysed as potential mediators were not adequately captured by the available measures. For example, hair cortisol was examined in 2cm long hair samples, and thus reflected approximately two months' exposure to circulating cortisol. While there are advantages in using hair cortisol over other more transient cortisol measures (e.g., in blood or saliva) two months is still a relatively short interval given that these data were collected on average two years after discrimination was assessed. In addition, research using hair cortisol is in its infancy and it is unclear whether 1 standard deviation increase in hair cortisol is clinically meaningful (56). Further, cortisol does not just reflect stress-related activation of the HPA axis. Cortisol parameters are

For peer review only - http://bmjopen.bmj.com/site/about/guidelines.xhtml

altered in weight-related conditions such as sub-clinical hypercortisolism and diabetes (57,58). Cortisol dynamics are complex, with heightened as well as blunted responses associated with weight-related ill-health (59,60). These complexities warrant attention in future work. It is also possible that other stress-related biological pathways (such as chronic inflammation) could play a role in the weight discrimination-health relationship. Physical activity was self-reported in response to three basic questions on the frequency of light, moderate or vigorous exercise. Comparisons of self-reported versus objective measures of physical activity have shown that there are often substantial differences between what people say and what they actually do (61). Our measures of smoking behaviour and alcohol consumption were crude, as both factors were self-reported using a single item measure. Further research should aim to replicate our analyses with different and more detailed measures of the constructs we have examined (stress, physical inactivity, smoking, and alcohol consumption) to establish whether the null results of our mediation analyses are the result of limitations in our measures (such as self-reported health risk behaviours) or an accurate representation of the causal pathways linking perceived weight discrimination with poorer health and wellbeing outcomes.

Strengths of the study include the large sample, prospective study design, and assessment of multiple mediators and outcomes. However, there were several limitations. Weight was not measured in the same data collection wave as discrimination, and participants may have changed weight status since experiencing discrimination. The prevalence of weight discrimination in the sample was low which limits the generalisability of our findings. Further not all participants who perceived weight discrimination provided hair cortisol samples meaning we may not have been able to detect small effects. We did not have complete data on weight, and it is possible that people most troubled by their weight were more likely to decline to be weighed. It is also possible that individuals most vulnerable to negative effects of weight discrimination opted not to complete the item on weight discrimination. The discrimination questions asked about five broad discriminatory situations and was not tailored for weight discrimination. This may have helped avoid bias or priming, as participants were able to attribute multiple reasons for their experience of discrimination(31–34,47). However, other measures with more specific items on experiences weight discrimination, for example having to pay more on public transport for occupying two passenger seats or being viewed unfavourably as a potential romantic partner, may have garnered different results. Our measure was not validated to assess weight discrimination. The use of tools such as the Stigmatizing Situations Inventory (62) or the Fat Phobia Scale (63) that including specific items on weight discrimination, weight bias internalization and experience of weight discrimination by close family, romantic partners or healthcare professionals may also have produced different findings. Further, weight discrimination was only measured on one occasion meaning we

For peer review only - http://bmjopen.bmj.com/site/about/guidelines.xhtml

had insufficient information of the dose or duration or this form of discrimination. This increases the risk of misclassification bias.

Finally, while the data were drawn from a representative sample, there was missing data and loss to follow-up, meaning the results may not be representative of the target population. In addition, as the sample comprised predominantly white middle-aged and older adults. The results may not generalise to other samples, in particular, younger samples who may be more likely to report weight discrimination (64). We did not have data on other negative emotional states such as psychological distress and anxiety which are likely relevant to the links between weight discrimination, health, and wellbeing.

Overall, these results provide further prospective evidence linking weight-based discrimination with poorer health and wellbeing. However, while perceived weight discrimination was associated with greater stress exposure and physical inactivity, these variables explained only a small part of the association between weight discrimination and poorer health and wellbeing. This leaves unanswered questions about the pathways through which weight discrimination adversely impacts health and wellbeing. Further research is required using large prospective-based studies that have been specifically designed to assess these questions and to test unmeasured factors (e.g., diet and eating behaviour). There is a great need to include more diverse samples in this future work to improve the generalisability of study findings and also to understand intersectional issues (e.g., how weight discrimination may interact with other forms of discrimination such as racism to influence health). Understanding of mechanisms of action is required if policies and interventions to mitigate the health burden of weight discrimination are to be developed. This is imperative as the literature and the findings of the current study underscore the need to tackle weight discrimination to improve population health.

For peer review only

For peer review only - http://bmjopen.bmj.com/site/about/guidelines.xhtml

Declarations

Contributor statement: RAH conducted statistical analyses, wrote, edited and reviewed the manuscript. SEJ conducted statistical analyses, wrote, edited and reviewed the manuscript. EC edited and reviewed the manuscript. AS, edited and reviewed the manuscript.

Competing interests: None

Funding: This research was supported by the Economic and Social Research Council (https://esrc.ukri.org/), grant number ES/R005990/1(awarded to AS and SEJ), and the Academy of Medical Sciences/the Wellcome Trust/the Government Department of Business, Energy and Industrial Strategy/the British Heart Foundation/Diabetes UK Springboard Award (SBF006\1036) (awarded to RH). The funders had no role in study design, data collection and analysis, decision to publish, or preparation of the manuscript.

Data sharing: Data from the English Longitudinal Study of Ageing are available to download from g2aging.org.

Ethics approval statement: Ethical approval for ELSA was obtained from the London Multicentre Research and Ethics Committee (MREC/01/02/91). All participants provided informed consent.

For peer review only

For peer review only - http://bmjopen.bmj.com/site/about/guidelines.xhtml

References

1. Health Survey for England [Internet]. 2019 [cited 2020 Mar 19]. Adults' average BMI. Available from: http://healthsurvey.hscic.gov.uk/data-visualisation/data-visualisation/explore-the-trends/weight/adult/bmi.aspx

2. NHS Digital. Statistics on Obesity, Physical Activity and Diet, England, 2019 [Internet]. 2019 [cited 2020 Mar 19]. Available from: https://digital.nhs.uk/data-and-information/publications/statistical/statistics-on-obesity-physical-activity-and-diet/statistics-on-obesity-physical-activity-and-diet-england-2019/part-3-adult-obesity

3. Wardle J, Boniface D. Changes in the distributions of body mass index and waist circumference in English adults, 1993/1994 to 2002/2003. Int J Obes. 2008;32(3):527–32.

4. Puhl RM, Heuer CA. The Stigma of Obesity: A Review and Update. Obesity. 2009 May 1;17(5):941–64.

5. Emmer C, Bosnjak M, Mata J. The association between weight stigma and mental health: A meta-analysis. Obes Rev. 2020;21(1):e12935.

6. Puhl R, Suh Y. Health Consequences of Weight Stigma: Implications for Obesity Prevention and Treatment. Curr Obes Rep. 2015 Apr 1;4(2):182–90.

7. Sutin AR, Stephan Y, Carretta H, Terracciano A. Perceived discrimination and physical, cognitive, and emotional health in older adulthood. Am J Geriatr Psychiatry Off J Am Assoc Geriatr Psychiatry. 2015 Feb;23(2):171–9.

8. Hatzenbuehler ML, Keyes KM, Hasin DS. Associations between perceived weight discrimination and the prevalence of psychiatric disorders in the general population. Obes Silver Spring Md. 2009 Nov;17(11):2033–9.

9. Hackman J, Maupin J, Brewis AA. Weight-related stigma is a significant psychosocial stressor in developing countries: Evidence from Guatemala. Soc Sci Med 1982. 2016;161:55–60.

10. Sutin AR, Stephan Y, Grzywacz JG, Robinson E, Daly M, Terracciano A. Perceived weight discrimination, changes in health, and daily stressors. Obesity. 2016 Oct 1;24(10):2202–9.

11. Schafer MH, Ferraro KF. The stigma of obesity: does perceived weight discrimination affect identity and physical health? Soc Psychol Q. 2011;74(1):76–97.

12. Sutin AR, Terracciano A. Sources of weight discrimination and health. Stigma Health. 2017;2(1):23–7.

13. Sutin AR, Stephan Y, Terracciano A. Weight Discrimination and Risk of Mortality. Psychol Sci. 2015 Nov 1;26(11):1803–11.

14. Tomiyama AJ. Weight stigma is stressful. A review of evidence for the Cyclic Obesity/Weight-Based Stigma model. Appetite. 2014 Nov;82:8–15.

For peer review only - http://bmjopen.bmj.com/site/about/guidelines.xhtml

15. Schvey NA, Puhl RM, Brownell KD. The stress of stigma: exploring the effect of weight stigma on cortisol reactivity. Psychosom Med. 2014 Feb;76(2):156–62.

16. Himmelstein MS, Incollingo Belsky AC, Tomiyama AJ. The weight of stigma: cortisol reactivity to manipulated weight stigma. Obes Silver Spring Md. 2015 Feb;23(2):368–74.

17. Puhl RM, Brownell KD. Confronting and coping with weight stigma: an investigation of overweight and obese adults. Obes Silver Spring Md. 2006 Oct;14(10):1802–15.

18. McEwen BS, Gianaros PJ. Central role of the brain in stress and adaptation: links to socioeconomic status, health, and disease. Ann N Y Acad Sci. 2010 Feb;1186:190–222.

19. Jackson SE, Kirschbaum C, Steptoe A. Perceived weight discrimination and chronic biochemical stress: A population-based study using cortisol in scalp hair. Obes Silver Spring Md. 2016 Dec;24(12):2515–21.

20. Tomiyama AJ, Epel ES, McClatchey TM, Poelke G, Kemeny ME, McCoy SK, et al. Associations of weight stigma with cortisol and oxidative stress independent of adiposity. Health Psychol Off J Div Health Psychol Am Psychol Assoc. 2014 Aug;33(8):862–7.

21. Born JM, Lemmens SGT, Rutters F, Nieuwenhuizen AG, Formisano E, Goebel R, et al. Acute stress and food-related reward activation in the brain during food choice during eating in the absence of hunger. Int J Obes. 2010;34(1):172–81.

22. Groesz LM, McCoy S, Carl J, Saslow L, Stewart J, Adler N, et al. What is eating you? Stress and the drive to eat. Appetite. 2012 Apr;58(2):717–21.

23. Kandiah J, Yake M, Jones J, Meyer M. Stress influences appetite and comfort food preferences in college women. Nutr Res. 2006;26(3):118–23.

24. Wu YK, Berry DC. Impact of weight stigma on physiological and psychological health outcomes for overweight and obese adults: A systematic review. J Adv Nurs. 2018;74(5):1030–42.

25. Salvia MG, Ritholz MD, Craigen KLE, Quatromoni PA. Women's perceptions of weight stigma and experiences of weight-neutral treatment for binge eating disorder: a qualitative study. eClinicalMedicine. 2023 Feb 1;56:101811.

26. Vucetic Z, Reyes TM. Central dopaminergic circuitry controlling food intake and reward: implications for the regulation of obesity. Wiley Interdiscip Rev Syst Biol Med. 2010 Sep 1;2(5):577–93.

27. Bethancourt HJ, Rosenberg DE, Beatty T, Arterburn DE. Barriers to and Facilitators of Physical Activity Program Use Among Older Adults. Clin Med Res. 2014 Jan 10;cmr.2013.1171.

28. Steptoe A, Breeze E, Banks J, Nazroo J. Cohort profile: the English Longitudinal Study of Ageing. Int J Epidemiol. 2013 Dec;42(6):1640–8.

[dataset] 29. Banks J, Batty GD, Breedvelt J, Coughlin K, Marmot M, Nazroo J, et al. English Longitudinal Study of Ageing: Waves 0-9, 1998-2019. [data collection]. [Internet]. 37th Edition. SN; 2021. (5050). Available from: DOI: 10.5255/UKDA-SN-5050-24

For peer review only

For peer review only - http://bmjopen.bmj.com/site/about/guidelines.xhtml

30. Smith J, Ryan L, Sonnega A, Weir DR. Psychosocial and lifestyle questionnaire 2006–2016: Documentation report core section. Ann Arbor, MI, USA.: The HRS Psychosocial Working Group:; 2017.

31. Jackson SE, Hackett RA, Steptoe A. Associations between age discrimination and health and wellbeing: cross-sectional and prospective analysis of the English Longitudinal Study of Ageing. Lancet Public Health. 2019 Apr 1;4(4):e200–8.

32. Jackson SE, Hackett RA, Pardhan S, Smith L, Steptoe A. Association of Perceived Discrimination With Emotional Well-being in Older Adults With Visual Impairment. JAMA Ophthalmol. 2019 01;137(7):825–32.

33. Scott W, Jackson SE, Hackett RA. Perceived discrimination, health, and well-being among adults with and without pain: a prospective study. PAIN. 2022;163(2):258–66.

34. Jackson SE, Hackett RA, Grabovac I, Smith L, Steptoe A. Perceived discrimination, health and wellbeing among middle-aged and older lesbian, gay and bisexual people: A prospective study. PLOS ONE. 2019 May 10;14(5):e0216497.

35. Jackson SE, Beeken RJ, Wardle J. Obesity, perceived weight discrimination, and psychological well-being in older adults in England. Obesity. 2015 May 1;23(5):1105–11.

36. Jackson SE, Steptoe A, Beeken RJ, Croker H, Wardle J. Perceived weight discrimination in England: a population-based study of adults aged ⩾50 years. Int J Obes. 2015 May;39(5):858–64.

37. Jackson SE, Beeken RJ, Wardle J. Perceived weight discrimination and changes in weight, waist circumference, and weight status. Obesity. 2014 Dec 1;22(12):2485–8.

38. Jackson SE, Steptoe A. Association between perceived weight discrimination and physical activity: a population-based study among English middle-aged and older adults. BMJ Open. 2017 Mar 1;7(3):e014592.

39. Jackson SE, Steptoe A. Obesity, perceived weight discrimination, and hair cortisol: a population-based study. Psychoneuroendocrinology. 2018 Dec 1;98:67–73.

40. Steptoe A, Jackson SE. The Life Skills of Older Americans: Association with Economic, Psychological, Social, and Health Outcomes. Sci Rep. 2018 Jul 5;8(1):9669.

41. Radloff LS. The CES-D scale. Appl Psychol Meas. 1977;1(3):385–401.

42. Steffick DE. Documentation of affective functioning measures in the Health and Retirement Study. HRS Documentation Report DR-005; 2000.

43. Hyde M, Wiggins RD, Higgs P, Blane DB. A measure of quality of life in early old age: the theory, development and properties of a needs satisfaction model (CASP-19). Aging Ment Health. 2003;7(3):186–94.

44. Diener E, Emmons RA, Larsen RJ, Griffin S. The satisfaction with life scale. J Assess. 1985;49(1):71–5.

For peer review only - http://bmjopen.bmj.com/site/about/guidelines.xhtml

45. Jackson SE, Kirschbaum C, Steptoe A. Hair cortisol and adiposity in a population-based sample of 2,527 men and women aged 54 to 87 years. Obesity. 2017;25(3):539–44.

46. Hatzenbuehler ML, Phelan JC, Link BG. Stigma as a Fundamental Cause of Population Health Inequalities. Am J Public Health. 2013 May;103(5):813–21.

47. Amirova A, Rimes KA, Hackett RA. Perceived discrimination in middle-aged and older adults: Comparison between England and the United States. Front Public Health [Internet]. 2022 [cited 2023 Feb 21];10. Available from: https://www.frontiersin.org/articles/10.3389/fpubh.2022.975776

48. Pascoe EA, Smart Richman L. Perceived discrimination and health: A meta-analytic review. Psychol Bull. 2009;135(4):531–54.

49. Puhl RM, Moss-Racusin CA, Schwartz MB. Internalization of weight bias: Implications for binge eating and emotional well-being. Obes Silver Spring Md. 2007 Jan;15(1):19–23.

50. Sutin A, Robinson E, Daly M, Terracciano A. Weight discrimination and unhealthy eating-related behaviors. Appetite. 2016 01;102:83–9.

51. Sabin JA, Marini M, Nosek BA. Implicit and Explicit Anti-Fat Bias among a Large Sample of Medical Doctors by BMI, Race/Ethnicity and Gender. PLOS ONE. 2012 Nov 7;7(11):e48448.

52. Phelan SM, Burgess DJ, Yeazel MW, Hellerstedt WL, Griffin JM, van Ryn M. Impact of weight bias and stigma on quality of care and outcomes for patients with obesity. Obes Rev. 2015;16(4):319–26.

53. Hackett RA, Hudson JL, Chilcot J. Loneliness and type 2 diabetes incidence: findings from the English Longitudinal Study of Ageing. Diabetologia. 2020 Nov 1;63(11):2329–38.

54. Valtorta NK, Kanaan M, Gilbody S, Ronzi S, Hanratty B. Loneliness and social isolation as risk factors for coronary heart disease and stroke: systematic review and meta-analysis of longitudinal observational studies. Heart. 2016 Jul 1;102(13):1009–16.

55. Jylhä M. What is self-rated health and why does it predict mortality? Towards a unified conceptual model. Soc Sci Med 1982. 2009 Aug;69(3):307–16.

56. Iob E, Steptoe A. Cardiovascular Disease and Hair Cortisol: a Novel Biomarker of Chronic Stress. Curr Cardiol Rep. 2019 Aug 30;21(10):116.

57. Chiodini I. Diagnosis and Treatment of Subclinical Hypercortisolism. J Clin Endocrinol Metab. 2011 May 1;96(5):1223–36.

58. Hackett RA, Steptoe A, Kumari M. Association of Diurnal Patterns in Salivary Cortisol With Type 2 Diabetes in the Whitehall II Study. J Clin Endocrinol Metab. 2014 Dec 1;99(12):4625–31.

59. McEwen BS. Stress, adaptation, and disease. Allostasis and allostatic load. Ann N Y Acad Sci. 1998 May 1;840:33–44.

For peer review only

For peer review only - http://bmjopen.bmj.com/site/about/guidelines.xhtml

60. Steptoe A, Hackett RA, Lazzarino AI, Bostock S, Marca RL, Carvalho LA, et al. Disruption of multisystem responses to stress in type 2 diabetes: Investigating the dynamics of allostatic load. Proc Natl Acad Sci. 2014 Nov 4;111(44):15693–8.

61. Kapteyn A, Banks J, Hamer M, Smith JP, Steptoe A, Soest A van, et al. What they say and what they do: comparing physical activity across the USA, England and the Netherlands. J Epidemiol Community Health. 2018 Jun 1;72(6):471–6.

62. Myers A, Rosen JC. Obesity stigmatization and coping: relation to mental health symptoms, body image, and self-esteem. Int J Obes Relat Metab Disord J Int Assoc Study Obes. 1999 Mar;23(3):221–30.

63. Bacon JG, Scheltema KE, Robinson BE. Fat phobia scale revisited: the short form. Int J Obes Relat Metab Disord J Int Assoc Study Obes. 2001 Feb;25(2):252–7.

64. Puhl RM, Andreyeva T, Brownell KD. Perceptions of weight discrimination: prevalence and comparison to race and gender discrimination in America. Int J Obes. 2008 Jun;32(6):992–1000.

For peer review only

For peer review only - http://bmjopen.bmj.com/site/about/guidelines.xhtml

Figures legends

Figure 1. Mediation model of associations between perceived weight discrimination and health and wellbeing outcomes via health behaviours and stress exposure

For peer review only

For peer review only - http://bmjopen.bmj.com/site/about/guidelines.xhtml

Figures

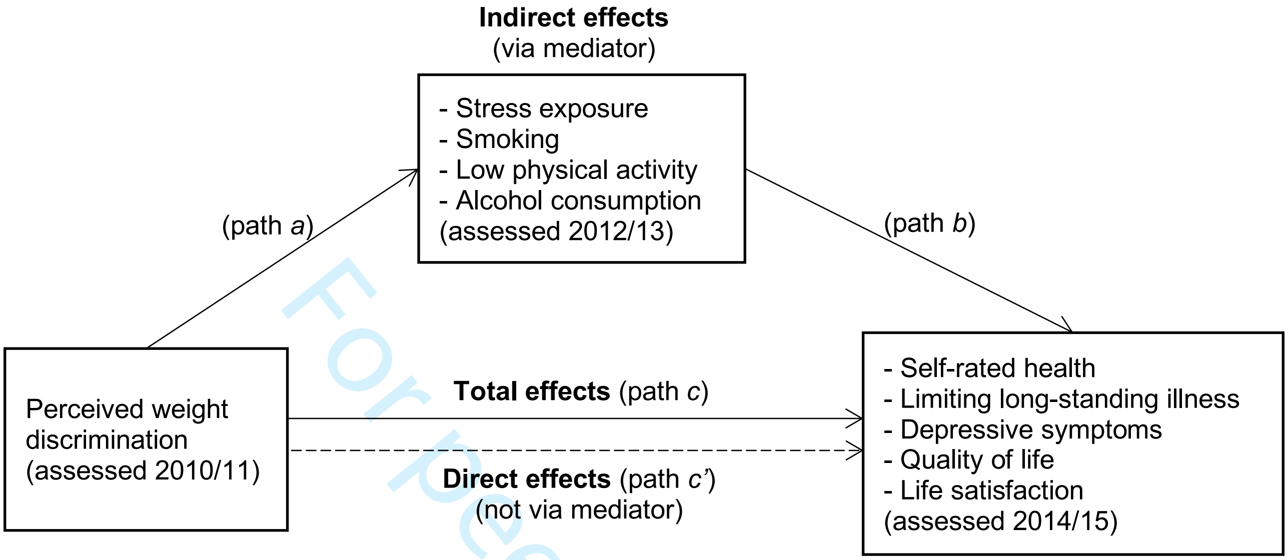

Figure 1. Mediation model of associations between perceived weight discrimination and health and wellbeing outcomes via health behaviours and stress exposure

For peer review only

Sensitivity analyses - Tables

**Supplementary Table 1** Sample characteristics at baseline (Wave 5) **all weight categories**					
		**Whole sample (*n*=5965)**	**Perceived weight discrimination (*n*=219)**	**No perceived weight discrimination (*n*=5746)**	***p***
Age (years), mean (SD)		66.56 (8.13)	62.16 (6.74)	66.72 (8.13)	<0.001
Sex, % (*n*)					
	Men	44.5 (2655)	35.2 (77)	44.9 (2578)	=0.005
	Women	55.5 (3310)	64.8 (142)	55.1 (3168)	-
Ethnicity, % (*n*)					
	White	97.9 (5841)	97.7 (214)	97.9 (5627)	0.829
	Other	2.1 (124)	2.3 (5)	2.1 (199)	-
Wealth quintile, % (*n*)					
	1 (poorest)	14.6 (868)	26.0 (57)	14.1 (811)	<0.001
	2	19.8 (1183)	30.6 (67)	19.4 (1116)	-
	3	19.9 (1190)	18.3 (40)	20.0 (1150)	-
	4	21.9 (1306)	15.5 (34)	22.1 (1272)	-
	5 (richest)	23.8 (1418)	9.6 (21)	24.3 (1397)	-
BMI (kg/m^2), mean (SD)		28.27 (5.22)	36.05 (6.69)	27.98 (4.92)	<0.001
BMI, body mass index.					

For peer review only

Supplementary Table 2 Cross-sectional and prospective associations of perceived weight discrimination with health and wellbeing outcomes across **all weight categories**

	Cross-sectional (Wave 5)		Prospective (Wave 7)	
	**No perceived weight discrimination**	**Perceived weight discrimination**	**No perceived weight discrimination**	**Perceived weight discrimination**
Fair/poor self-rated health				
n included in analysis	5754	217	5122	197
% (*n*) reporting outcome	20.8 (1197)	48.4 (105)	24.4 (1251)	52.8 (104)
Adjusted OR [95% CI]	1.00 (ref)	2.32 [1.71; 3.16]	1.00 (ref)	1.66 [1.15; 2.42]
p		<0.001		0.008
Limiting long-standing illness				
n included in analysis	5743	218	5172	199
% (*n*) reporting outcome	30.3 (1741)	55.0 (120)	33.9 (1755)	56.3 (112)
Adjusted OR [95% CI]	1.00 (ref)	1.91 [1.42; 2.58]	1.00 (ref)	1.32 [0.92; 1.88]
p		<0.001		0.130
Depressive symptoms				
n included in analysis	5695	219	5027	197
% (*n*) reporting outcome	11.7 (668)	27.9 (61)	10.4 (525)	30.5 (60)
Adjusted OR [95% CI]	1.00 (ref)	2.05 [1.46; 2.89]	1.00 (ref)	2.74 [1.84; 4.09]
p		<0.001		<0.001
Quality of life (range 0-57)				
n included in analysis	5497	212	4318	162
Mean (SD) score	41.96 (8.34)	34.26 (9.55)	42.54 (8.38)	36.60 (9.40)
Adjusted *B* [95% CI]	Ref	-6.03 [-7.18; -4.88]	Ref	-0.21 [-1.12; 0.70]
p		<0.001		0.651
Life satisfaction (range 0-30)				
n included in analysis	5577	207	4490	167
Mean (SD) score	21.07 (6.08)	17.49 (7.60)	21.28 (6.01)	18.32 (7.32)
Adjusted *B* [95% CI]	Ref	-2.50 [-3.37; -1.63]	Ref	-0.45 [-1.16; 0.26]
p		<0.001		0.213

OR, odds ratio. CI, confidence interval. SD, standard deviation.
ORs, *B*s and 95% CIs are adjusted for age, sex, ethnicity, wealth, and body mass index. Prospective results are additionally adjusted for status/score on the outcome variable at baseline (Wave 5).
Ref = reference category.

For peer review only

**Supplementary Table 3** Sample characteristics at baseline (Wave 5) **in obese participants**					
		**Whole sample (*n*=1853)**	**Perceived weight discrimination (*n*=184)**	**No perceived weight discrimination (*n*=1669)**	***p***
Age (years), mean (SD)		65.81 (7.76)	62.03 (6.66)	66.23 (7.76)	<0.001
Sex, % (*n*)					
	Men	40.5 (751)	33.2 (61)	41.3 (690)	=0.032
	Women	59.5 (1102)	66.8 (123)	58.7 (979)	-
Ethnicity, % (*n*)					
	White	97.8 (1812)	97.3 (179)	97.8 (1633)	0.624
	Other	2.2 (41)	2.7 (5)	2.2 (36)	-
Wealth quintile, % (*n*)					
	1 (poorest)	19.9 (368)	27.7 (51)	19.0 (317)	<0.001
	2	22.9 (424)	29.3 (54)	22.2 (370)	-
	3	21.3 (395)	19.6 (36)	21.5 (359)	-
	4	19.3 (358)	15.8 (29)	19.7 (329)	-
	5 (richest)	16.6 (308)	7.6 (14)	17.6 (294)	-
BMI (kg/m^2), mean (SD)		34.31 (4.13)	37.80 (5.68)	33.92 (3.73)	<0.001
BMI, body mass index.					

For peer review only

Supplementary Table 4 Cross-sectional and prospective associations of perceived weight discrimination with health and wellbeing outcomes across **in obese participants**

	Cross-sectional (Wave 5)		Prospective (Wave 7)	
	**No perceived weight discrimination**	**Perceived weight discrimination**	**No perceived weight discrimination**	**Perceived weight discrimination**
Fair/poor self-rated health				
n included in analysis	1669	182	1478	167
% (*n*) reporting outcome	28.4 (474)	47.3 (86)	32.3 (478)	53.9 (90)
Adjusted OR [95% CI]<br>*p*	1.00 (ref)	1.74 [1.23; 2.46]<br>=0.002	1.00 (ref)	1.56 [1.02; 2.39]<br>0.040
Limiting long-standing illness				
n included in analysis	1668	183	1493	169
% (*n*) reporting outcome	39 (651)	53.6 (98)	43.4 (648)	56.2 (95)
Adjusted OR [95% CI]<br>*p*	1.00 (ref)	1.47 [1.05; 2.01]<br>=0.027	1.00 (ref)	1.01 [0.70; 1.56]<br>0.832
Depressive symptoms				
n included in analysis	1654	184	1451	167
% (*n*) reporting outcome	14.1 (234)	28.8 (53)	11.9 (172)	29.9 (50)
Adjusted OR [95% CI]<br>*p*	1.00 (ref)	1.97 [1.34; 2.88]<br><0.001	1.00 (ref)	2.15 [1.36; 3.41]<br><0.001
Quality of life (range 0-57)				
n included in analysis	1581	178	1225	137
Mean (SD) score	40.85 (8.44)	34.44 (9.50)	41.22 (8.61)	36.61 (9.16)
Adjusted *B* [95% CI]<br>*p*	Ref	-5.24 [-6.57; -3.92]<br><0.001	Ref	0.08 [-1.01; 1.17]<br>0.888
Life satisfaction (range 0-30)				
n included in analysis	1604	173	1273	139
Mean (SD) score	20.57 (6.28)	17.51 (7.67)	20.66 (6.23)	18.60 (7.17)
Adjusted *B* [95% CI]<br>*p*	Ref	-2.00 [-3.03; -0.98]<br><0.001	Ref	0.09 [-0.75; 0.94]<br>0.820

OR, odds ratio. CI, confidence interval. SD, standard deviation.
ORs, *B*s and 95% CIs are adjusted for age, sex, ethnicity, wealth, and body mass index. Prospective results are additionally adjusted for status/score on the outcome variable at baseline (Wave 5).
Ref = reference category.

For peer review only

Supplementary Table 5 Cross-sectional and prospective associations of perceived weight discrimination in **any domain (ever vs never)** with health and wellbeing outcomes **in overweight and obese participants**

		Cross-sectional (Wave 5)		Prospective (Wave 7)	
		**No perceived weight discrimination**	**Perceived weight discrimination**	**No perceived weight discrimination**	**Perceived weight discrimination**
Fair/poor self-rated health					
	n included in analysis	4090	248	3670	223
	% (*n*) reporting outcome	22.5 (919)	44.0 (109)	26.2 (960)	51.1 (114)
	Adjusted OR [95% CI]<br>*p*	1.00 (ref)	1.74 [1.29; 2.35]<br><0.001	1.00 (ref)	1.68 [1.17; 2.41]<br>0.005
Limiting long-standing illness					
	n included in analysis	4089	249	3705	225
	% (*n*) reporting outcome	32.0 (1309)	54.6 (136)	36.0 (1333)	57.3 (129)
	Adjusted OR [95% CI]<br>*p*	1.00 (ref)	1.79 [1.34; 2.39]<br><0.001	1.00 (ref)	1.33 [0.94; 1.89]<br>0.105
Depressive symptoms					
	n included in analysis	4058	250	3609	222
	% (*n*) reporting outcome	11.9 (483)	25.6 (64)	10.7 (385)	28.4 (63)
	Adjusted OR [95% CI]<br>*p*	1.00 (ref)	1.75 [1.24; 2.45]<br><0.001	1.00 (ref)	2.54 [1.71; 3.77]<br><0.001
Quality of life (range 0-57)					
	n included in analysis	3902	243	3080	180
	Mean (SD) score	41.76 (8.35)	34.85 (9.50)	42.20 (8.47)	37.17 (9.39)
	Adjusted *B* [95% CI]<br>*p*	Ref	-5.53 [-6.64; -4.42]<br><0.001	Ref	0.24 [-0.67; 1.15]<br>0.608
Life satisfaction (range 0-30)					
	n included in analysis	3968	239	3210	190
	Mean (SD) score	21.05 (6.02)	18.05 (7.41)	21.22 (6.04)	18.89 (7.09)
	Adjusted *B* [95% CI]<br>*p*	Ref	-1.87 [-2.70; -1.04]<br><0.001	Ref	-0.07 [-0.76; 0.62]<br>0.844

OR, odds ratio. CI, confidence interval. SD, standard deviation.
ORs, *B*s and 95% CIs are adjusted for age, sex, ethnicity, wealth, and body mass index. Prospective results are additionally adjusted for status/score on the outcome variable at baseline (Wave 5).
Ref = reference category.

For peer review only

Supplementary materials: Questionnaire measures

Everyday discrimination

In your day-to-day life, how often have any of the following things happened to you:

	Never (0)	Less than once a year (1)	Few times a year (2)	Few times a month (3)	At least once a week(4)	Almost everyday (5)
You are treated with less respect or courtesy						
You receive poorer service than other people in restaurants and stores						
People act as if they think you are not clever						
You are threatened or harassed						
You receive poorer service or treatment than other people from doctors or hospitals						

If you answered yes to any of the above, what do you attribute this experience to

Gender
Race
Age
Weight
Physical disability
Physical appearance
Sexual orientation
Financial status
Other reason

Self-rated health

"Would you say your health is… poor/fair/good/very good/excellent?"

Limiting long-standing illness

1) "Do you have any long-standing illness, disability, or infirmity? By long-standing I mean anything that has troubled you over a period of time or that is likely to affect you over a period of time."
2) If yes, "Does this illness or disability limit your activities in any way?"

Satisfaction With Life Scale

(Diener et al., The satisfaction with life scale. J Assess. 1985;49(1):71–5).

How much of the time during the past week…
1) In most ways my life is close to my ideal.
2) The conditions of my life are excellent.
3) I am satisfied with my life.
4) So far, I have gotten the important things I want in life.
5) If I could live my life over, I would change almost nothing.
Answers range from 0 (Strongly Disagree) to 6 (Strongly agree)

For peer review only

Center for Epidemiologic Studies Depression (CESD) scale

(Radloff LS. The CES-D scale. Appl Psychol Meas. 1977;1(3):385–401).

In the past month	Yes	No
1) ... did you feel depressed?		
2) ... did you feel everything you did was an effort?		
3) ... was your sleep restless?		
4) ... were you happy?		
5) ... did you feel lonely?		
6) ... did you enjoy life?		
7) ... did you feel sad?		
8) ... were you unable to get going?		

CASP-19

(Hyde M., et al., A measure of quality of life in early old age: the theory, development and properties of a needs satisfaction model (CASP-19). Aging Ment Health. 2003;7(3):186–94).

	Often (0)	Sometimes (1)	Not often (2)	Never (3)
**1** My age prevents me from doing the things I would like to				
**2** I feel that what happens to me is out of my control				
**3** I feel free to plan for the future				
**4** I feel left out of things				
**5** I can do the things I want to do				
**6** Family responsibilities prevent me from doing what I want to do				
**7** I feel that I can please myself what I do				
**8** My health stops me from doing things I want to				
**9** Shortage of money stops me from doing the things I want to do				
**10** I look forward to each day				
**11** I feel that my life has meaning				
**12** I enjoy the things that I do				
**13** I enjoy being in the company of others				
**14** On balance, I look back on my life with a sense of happiness				
**15** I feel full of energy these days				
**16** I choose to do things that I have never done before				
**17** I am satisfied with the way my life has turned out				
**18** I feel that life is full of opportunities				
**19** I feel that the future looks good for me				

For peer review only

STROBE 2007 (v4) Statement—Checklist of items that should be included in reports of *cohort studies*

Section/Topic	Item #	Recommendation	Reported on page #
**Title and abstract**	1	(*a*) Indicate the study's design with a commonly used term in the title or the abstract	1,2
		(*b*) Provide in the abstract an informative and balanced summary of what was done and what was found	2
**Introduction**			
Background/rationale	2	Explain the scientific background and rationale for the investigation being reported	3-4
Objectives	3	State specific objectives, including any prespecified hypotheses	5
**Methods**			
Study design	4	Present key elements of study design early in the paper	5
Setting	5	Describe the setting, locations, and relevant dates, including periods of recruitment, exposure, follow-up, and data collection	5
Participants	6	(*a*) Give the eligibility criteria, and the sources and methods of selection of participants. Describe methods of follow-up	5
		(*b*) For matched studies, give matching criteria and number of exposed and unexposed	
Variables	7	Clearly define all outcomes, exposures, predictors, potential confounders, and effect modifiers. Give diagnostic criteria, if applicable	5-8
Data sources/ measurement	8*	For each variable of interest, give sources of data and details of methods of assessment (measurement). Describe comparability of assessment methods if there is more than one group	5-8
Bias	9	Describe any efforts to address potential sources of bias	
Study size	10	Explain how the study size was arrived at	5 & 9
Quantitative variables	11	Explain how quantitative variables were handled in the analyses. If applicable, describe which groupings were chosen and why	8
Statistical methods	12	(*a*) Describe all statistical methods, including those used to control for confounding	8
		(*b*) Describe any methods used to examine subgroups and interactions	
		(*c*) Explain how missing data were addressed	9
		(*d*) If applicable, explain how loss to follow-up was addressed	
		(*e*) Describe any sensitivity analyses	8-9
**Results**			

For peer review only

Participants	13*	(a) Report numbers of individuals at each stage of study—eg numbers potentially eligible, examined for eligibility, confirmed eligible, included in the study, completing follow-up, and analysed	9
		(b) Give reasons for non-participation at each stage	9
		(c) Consider use of a flow diagram	
Descriptive data	14*	(a) Give characteristics of study participants (eg demographic, clinical, social) and information on exposures and potential confounders	9
		(b) Indicate number of participants with missing data for each variable of interest	9
		(c) Summarise follow-up time (eg, average and total amount)	
Outcome data	15*	Report numbers of outcome events or summary measures over time	10 and Table 1
Main results	16	(*a*) Give unadjusted estimates and, if applicable, confounder-adjusted estimates and their precision (eg, 95% confidence interval). Make clear which confounders were adjusted for and why they were included	10 and Tables 2-5
		(*b*) Report category boundaries when continuous variables were categorized	6-7
		(*c*) If relevant, consider translating estimates of relative risk into absolute risk for a meaningful time period	
Other analyses	17	Report other analyses done—eg analyses of subgroups and interactions, and sensitivity analyses	10
**Discussion**			
Key results	18	Summarise key results with reference to study objectives	10
**Limitations**			
Interpretation	20	Give a cautious overall interpretation of results considering objectives, limitations, multiplicity of analyses, results from similar studies, and other relevant evidence	11-12
Generalisability	21	Discuss the generalisability (external validity) of the study results	12
**Other information**			
Funding	22	Give the source of funding and the role of the funders for the present study and, if applicable, for the original study on which the present article is based	17

*Give information separately for cases and controls in case-control studies and, if applicable, for exposed and unexposed groups in cohort and cross-sectional studies.

Note: An Explanation and Elaboration article discusses each checklist item and gives methodological background and published examples of transparent reporting. The STROBE checklist is best used in conjunction with this article (freely available on the Web sites of PLoS Medicine at http://www.plosmedicine.org/, Annals of Internal Medicine at http://www.annals.org/, and Epidemiology at http://www.epidem.com/). Information on the STROBE Initiative is available at www.strobe-statement.org.

For peer review only
